# Tailored ozone activation on geometrical-site-dependent cobalt with selective coordination

**DOI:** 10.1038/s41467-025-61181-7

**Published:** 2025-07-01

**Authors:** Shenning Liu, Yuxian Wang, Ya Liu, Peihan Chen, Tao Kong, Xiaoguang Duan, Chunmao Chen, Hongqi Sun, Shaobin Wang

**Affiliations:** 1 State Key Laboratory of Heavy Oil Processing, China University of Petroleum-Beijing, Beijing, China; 2 School of Chemical Engineering, The University of Adelaide, Adelaide, SA Australia; 3 School of Molecular Sciences, The University of Western Australia, Perth, WA Australia

**Keywords:** Heterogeneous catalysis, Chemical engineering

## Abstract

Cobalt-containing spinel oxides are promising platforms to fine-tune the intrinsic activity/selectivity of their geometric sites in catalysis. However, the role of tetrahedrally occupied Co^2+^ (Co^2+^_Td_) and Co^3+^ in an octahedral site (Co^3+^_Oh_) in controlling the catalytic activity remains controversial. Herein, we investigated a geometrical-site-dependent catalytic activation of ozone respectively on the Co^2+^_Td_ and Co^3+^_Oh_ sites. The same exposure of [111] crystal facet is achieved by substituting those undesired sites with catalytically inactive cations. The highly spin-polarized Co^2+^_Td_ sites invoke strong orbital interactions and intensive electron transfer with the adsorbed O_3_ and become the active sites for selectively producing surface-bound hydroxyl radicals (^•^OH) and avoiding the formation of unfavorable singlet oxygen (^1^O_2_), resulting in a 17.6-fold increase in turnover frequency (TOF). This work enlightens the spin-polarized electronic states into regulating the reaction thermodynamics in transition metal oxide-induced catalysis and envisages the practical application potentials of geometric site engineered spinel oxides.

## Abstract (web summary)

Geometrical-site-dependent catalytic ozone activation is demonstrated on the Co^2+^_Td_ and Co^3+^_Oh_ sites in spinel oxides, where the spin-polarized Co^2+^_Td_ outperforms Co^3+^_Oh_ site in •OH generation, achieving a 17.6-fold increase in turnover frequency.

## Introduction

Earth-abundant transition metal (TM) oxides have been demonstrated to be catalytically active in oxygen-involved catalysis, for example, oxygen reduction reaction and Fenton catalysis for chemical conversions and environmental remediations^1^. The multi-valence states of TM centers guarantee a swift redox cycling process for a durable catalytic activity and facilitate the electron transfer from their partially occupied *3d* orbitals to the *2p* orbitals of oxygen^2^. Among TM oxides, spinel Co_3_O_4_ and its substitutional variants have attracted intensive research interests because of their tunable crystal structures and active sites, in which the tetrahedrally occupied Co^2+^ (Co^2+^_Td_) and octahedrally occupied Co^3+^ (Co^3+^_Oh_) within the structure provide a versatile geometrical substitution platform^3,4^. Thus, a variety of substituted spinel cobaltite (MCo_2_O_4_) and CoM_2_O_4_ (M for substituted metals) have been synthesized with distinctive activity^5,6^. Previous studies usually ascribed the activity variations to the substitution-induced changes in the physicochemical properties, which resulted in the deviation of the electron transfer ability^7,8^. Nevertheless, the intrinsic origin of the substitutional effects in cobalt catalysis remained ambiguous.

Recently, geometrical-site-dependent activity has been disclosed in cobalt-containing TM oxides^9,10^. Substituting the Co^2+^_Td_ or Co^3+^_Oh_ sites by cations with different reactivities rendered distinct activity deviations, which are intrinsically controlled by the altered local coordination environment^11^. The covalency competition between the M_Td_–O (metal–oxygen bond in a tetrahedral unit) and M_Oh_–O (metal–oxygen bond in an octahedral unit) can break the M_Td_–O–M_Oh_ backbones in spinels, resulting in the exposure of the non-oxygen-bonding metal^12^. And the high covalency competition enhances the electronic hybridization between the non-bonded metal *3d* and O *2p* orbitals, thereby promoting the catalytic activity. Additionally, the d-band positions of TM and the band overlap between the TM *3d* orbitals and the O *2p* orbitals^13,14^, as well as the filling degree of the *e*_*g*_ orbitals, have been proposed as descriptors to correlate the structure and activity of Co-containing spinel oxides with tetrahedral and octahedral coordination^15^.

These fundamental findings enlighten the excavation of the intricate mechanisms underlying cobalt catalysis, yet the dominant role of Co^2+^_Td_ and Co^3+^_Oh_ sites in modulating catalytic activity still remains controversial, even in the same catalytic system^2,16^. Most of the studies failed to regulate the exposed crystal facets of the spinel oxides by making them share the same facet, despite significant variations in reaction kinetics and thermodynamics across different exposed crystal facets^17^. Additionally, the spin polarization effects brought by spin-state variations in the substituted TM oxides are often overlooked in deciphering orbital hybridization states^18,19^. The asymmetric distributions of electrons in spin-up and spin-down channels demonstrate different electronic states, which significantly affect the reaction thermodynamics by modulating the positions of molecular orbitals and electronic communications with the reactants^20,21^.

Heterogeneous catalytic ozonation, as one of the prevailing advanced oxidation processes, is appealing for its high efficiency in abating aqueous recalcitrant pollutants^22^. Herein, to well elucidate the origin of the activity of the cobalt-based spinels for selective ozone activation, we synthesize Co_3_O_4_ and its geometrical-site substitutes (ZnCo_2_O_4_ and CoGa_2_O_4_) with the same [111] crystal facet via a facile sol-gel method. X-ray absorption spectroscopy (XAS) validates the successful substitution of the Co^2+^_Td_ and Co^3+^_Oh_ sites in Co_3_O_4_ by catalytically inactive Zn^2+^ and Ga^3+^ with fully occupied *3d* orbitals, respectively. By establishing the structural-activity relationship, the unsubstituted host Co cation sites are identified as the key factor governing O_3_ activation, with Co^2+^_Td_ sites exhibiting much higher catalytic activity than Co^3+^_Oh_ sites. The improved O_3_ utilization efficiency and enhanced selective ^•^OH generation by avoiding the formation of unfavorable ^1^O_2_ account for the higher activity of Co^2+^_Td_ sites compared to Co^3+^_Oh_ sites. Theoretical investigations decipher that the highly spin-polarized Co^2+^_Td_ sites fine-tune the position of the anti-bonding molecular orbitals formed by the spin-down channels of the Co^2+^_Td_-*3d* orbitals and O_3_ *2p* orbitals close to the Fermi level, favoring the O_3_ adsorption and activation. The massively generated surface-bound ^•^OH at Co^2+^_Td_ sites results in a high chemical oxygen demand (COD) removal efficiency as well as the broadened band removal ability of the organics, as indicated by three-dimensional excitation-emission-matrix (3D-EEM) spectra for treatment of real refinery wastewater in a fixed bed continuous-flow reactor, envisaging the practical application potential of the cobalt-based spinels.

## Results

### Characterization of the catalysts

**Fig. 1:**
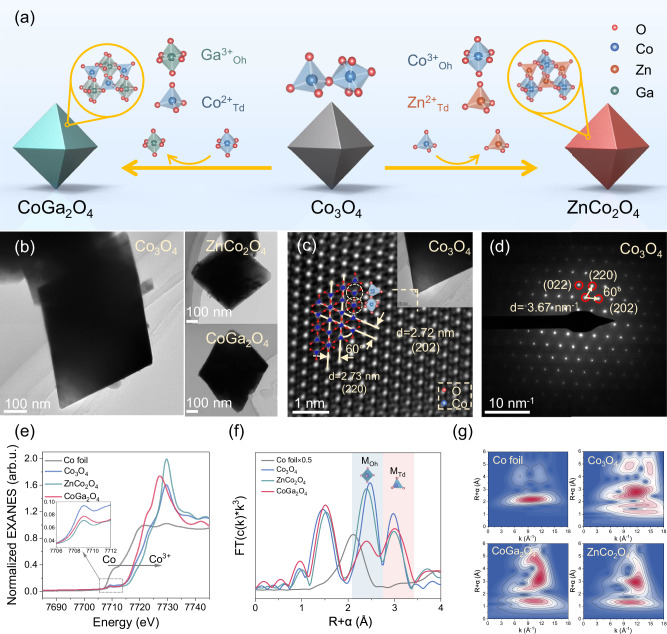
Synthesis and structures of the catalysts. **a** Schematic illustration of the synthetic process of the Co-based octahedral spinel oxides with Co^2+^ and Co^3+^ substitutions by Zn^2+^ and Ga^3+^, respectively. **b** Transmission electron microscopy (TEM) images of Co_3_O_4_, ZnCo_2_O_4_ and CoGa_2_O_4_ in [111] orientation. Atomic-resolved high-resolution TEM (HRTEM) image (**c**) and the corresponding selected area electron diffraction (SAED) image (**d**) of Co_3_O_4_. **e** Normalized Co K-edge X-ray absorption near-edge structure (XANES) spectra of the as-synthesized Co-based spinel oxides. Inset: enlarged features of the pre-edge peaks. **f** The corresponding Fourier transformed EXAFS (FT-EXAFS) spectra at R space. **g** EXAFS wavelet transforms of Co K-edge for Co foil and the as-synthesized Co-based spinels. Source data are provided as a Source Data file.

Substituting the host Co cations by Zn^2+^ and Ga^3+^ with fully occupied *3d* orbitals makes octahedral Co^3+^ (Co^3+^_Oh_) and tetrahedral Co^2+^ (Co^2+^_Td_) as the exclusive Co sites in ZnCo_2_O_4_ and CoGa_2_O_4_, respectively (Fig. 1a). The synthesis routes of pristine Co_3_O_4_ and its geometrical-site substitutions are schematically illustrated in Supplementary Fig. 1. Powder X-ray diffraction (XRD) patterns suggest that replacing Co^2^ by Zn^2+^ and Co^3+^ by Ga^3+^ in ZnCo_2_O_4_ and CoGa_2_O_4_ accordingly successfully substitute the Co^2+^_Td_/Co^3+^_Oh_ sites in the crystal structure of pristine Co_3_O_4_ while preserving the normal spinel crystal phase (Fig. 1b; Supplementary Fig. 2a). The significantly larger octahedral site preference energy (OSPE) of Co^3+^ compared to those of Co^2+^ and Zn^2+^ enables Co^3+^ to preferentially occupy the octahedral sites while resides the Co^2+^ and Zn^2+^ in tetrahedral sites^23^. The fully occupied 3 d orbitals in Ga^3+^ exclude the coordination geometry competition in CoGa_2_O_4_, making Co^2+^_Td_ as the dominant active sites. Additionally, the *A*_*1g*_ symmetry and *F*_*2g*_^*1*^ symmetry in CoGa_2_O_4_ and ZnCo_2_O_4_ shifted slightly compared to the Co_3_O_4_, respectively. This observation reinforces the normal spinel structures of the as-synthesized Co-based spinels and the minor presence of inversed Co^2+^_Oh_/Co^3+^_Td_ sites^24^ (Supplementary Fig. 3).

The atomic ratios of Co/Zn in ZnCo_2_O_4_ and Ga/Co in CoGa_2_O_4_ were determined using inductively coupled plasma optical emission spectrometry as 2.03 and 1.94, respectively, which are well matched to their theoretical stoichiometries. Moreover, the energy-dispersive X-ray spectroscopy (EDX) elemental mappings (Supplementary Figs. 4–6) indicate a uniform distribution of both the host cobalt and the substituted metal cations within these spinel oxides. Zn^2+^/Ga^3+^ with larger ionic radii than Co^2+^/Co^3+^ (0.58 vs. 0.60 Å, 0.61 vs. 0.62 Å) slightly altered the interplanar crystal spacing within the spinel structure (Supplementary Fig. 2b; Supplementary Table 1). This variation in interplanar crystal spacing induces covalency competition between M_Td_–O (metal–oxygen bond in tetrahedral unit) and M_Oh_–O (meta–-oxygen bond in octahedral unit) in the M_Td_–O–M_Oh_ backbone of the spinel crystal structure, affecting the exposed active sites and the electronic properties that govern the catalytic activity^25^.

Scanning electron microscopy (SEM) observations reveal that these spinel oxides demonstrate a similar octahedral morphology and centered particle size distributions with average sizes ranging from 327 to 466 nm (Fig. 1b; Supplementary Fig. 7a–c). This centric particle size range offers a solid basis for evaluating the intrinsic reactivity differences arising from geometrical sites (Supplementary Fig. 8). We also prepared MgCo_2_O_4_ and CoAl_2_O_4_ via the same synthesis protocol. However, these two spinel oxides display different morphologies compared to the others (Supplementary Fig. 7d, e), which might be attributed to the large deviations in ionic radius between Co and the substituted Mg/Al cations^26^.

The exposed crystal facets can act as the decisive factor in the activity of the metal-based catalysts by affecting both the reaction kinetics and pathways^27^. A rigorous investigation on the activity of the Co^2+^_Td_/Co^3+^_Oh_ geometrical sites requires pristine Co_3_O_4_ and its substitutes to have the same exposed crystal facet. In this study, the relative spatial positions of the lattice planes in reciprocal space were measured to determine their main exposed crystal facets by identifying the angles and spacings between the spots in selected area electron diffraction (SAED) images^28^ (Supplementary Fig. 9). For the as-synthesized Co_3_O_4_, ZnCo_2_O_4_, and CoGa_2_O_4_, the fixed angle of 60° with the crystal spacing of 3.67 nm^−1^ observed between the (220), (422), and (202) planes in their SAED patterns suggests that the [111] facet is the common exposed facet on their octahedrons^29^ (Fig. 1d, e; Supplementary Fig. 10a, b). Additionally, atom-resolved surface structure characterizations and the lattice spacing measurements further confirm the common exposure of [111] facet for these spinel oxides^29^ (Fig. 1c; Supplementary Fig. 10c, d).

Co sites symmetry and the valence states of pristine Co_3_O_4_ and its substitutes were investigated by X-ray absorption near-edge structure (XANES) (Fig. 1f). The intensity and position of the pre-edge peak (7709.1 eV) of Co K-edge spectrum provide clear insight into the symmetry of tetrahedral and octahedral sites within the spinel oxides because the highly non-centrosymmetry of the tetrahedral sites facilitates the *p* to *d* orbital transitions of the electrons^30^. Therefore, a narrower and more intense peak is expected in spinel oxides with a higher composition of tetrahedral sites than octahedral sites^31^. The inset in Fig. 1f shows that the pre-edge peak intensity for CoGa_2_O_4_ is greater than that for ZnCo_2_O_4_, suggesting that Ga^3+^ substitution results in more Co occupation in tetrahedral sites, while replacing Co^2+^ by Zn^2+^ favors the exposure of the octahedral sites. Moreover, the transformation from tetrahedral to octahedral site symmetry also shifts the pre-edge peak to a slightly increased position (∼0.3 eV).

Geometrical-site substitution also leads to variations of the main adsorption edges by affecting the valence states. Ga^3+^ substitution shifts the main adsorption edge (7721.3 eV) towards a lower energy, while incorporating Zn^2+^ raises the edge to a higher energy. This phenomenon suggests that the presence of Zn increases the oxidation state of Co while Ga drives Co to a reduced state^32^. The average oxidation states (AOS) of Co within Co_3_O_4_ and its substitutes were further calculated by establishing a linear dependence between the half of the normalized absorbance level in the XANES spectrum (E_1/2_) and Co oxidation states^25^ (Supplementary Fig. 11). The calculated AOS values of Co species in Co_3_O_4_, ZnCo_2_O_4_, CoGa_2_O_4_ and CoAl_2_O_4_ are 2.50, 2.92, 2.07, and 2.11, respectively. These results verify the successful substitution of the Co^2+^_Td_ and Co^3+^_Oh_ in pristine Co_3_O_4_ by Zn^2+^ and Ga^3+^/Al^3+^, respectively. X-ray photoelectron spectroscopy (XPS) analysis also corroborates these findings by examining the surface cobalt oxidation states (Supplementary Table 2; Supplementary Fig. 12).

Local geometric distributions of Co and the substitution cations in tetrahedral/octahedral sites of pristine Co_3_O_4_ and its substitutes were further deciphered by their Fourier-transform extended X-ray absorption fine structure (FT-EXAFS) (Fig. 1g) and wavelet transform analyses (Fig. 1h). For pristine Co_3_O_4_ containing both Co^2+^_Td_ and Co^3+^_Oh_ sites, three main peaks located in-between 1 and 2, at ~2.5, and at ~3.0 Å are observed, corresponding to the distances of Co–O shells, octahedrally coordinated cations to its closest neighboring metal ions in octahedral sites (Co_Oh_–Co_Oh_), and the closest neighboring Co cations in octahedral and tetrahedral sites (Co_Oh_–Co_Td_), respectively. It is evident that the octahedrally coordinated cations exhibit two bonding modes with the surrounding octahedral (~2.5 Å) and tetrahedral sites (~3.0 Å). The tetrahedrally coordinated cations have only one bonding mode with the neighboring octahedral sites (~3.0 Å). Therefore, the identification of the peaks at ~2.5 and 3.0 Å in ZnCo_2_O_4_ suggests that the Co^3+^ species are occupied in the octahedral sites. For CoGa_2_O_4_, despite that both peaks at ~2.5 and 3.0 Å are observed, the peak intensity at 2.5 Å is significantly lower than that in Co_3_O_4_ and ZnCo_2_O_4_, suggesting Co^2+^_Td_ still exists as the governing sites. In addition, the significant variations in peak intensity ratios of Co_Oh_–Co_Oh_ path to Co_Oh_–Co_Td_ path for CoGa_2_O_4_ and ZnCo_2_O_4_ compared to that of Co_3_O_4_ suggested that the inversed Co^2+^_Oh_/Co^3+^_Td_ sites act as the minor coordination-geometry-competitors against the dominant Co^2+^_Td_ and Co^3+^_Oh_ sites (Supplementary Fig. 13). The acquired coordination numbers of Co cations by fitting EXAFS spectra (Supplementary Fig. 14; Supplementary Table 3) further reveal that Co^3+^_Oh_ and Co^2+^_Td_ are the prevailing geometric sites in ZnCo_2_O_4_ and CoGa_2_O_4_, respectively.

The coordination environments surrounding the substituted Zn and Ga were also investigated. The fully occupied *3d* orbitals of Zn and Ga restrict their valence states to +2 and +3 accordingly when incorporated into the Co_3_O_4_ crystal scaffolds, as revealed by their high-resolution XPS spectra (Supplementary Fig. 15). Additionally, the acquired Ga K-edge XANES spectrum and the corresponding fitted EXAFS results indicate that Ga^3+^ substituted the Co^3+^ position and exhibited both octahedral and tetrahedral bonding modes (Supplementary Fig. 16; Supplementary Table 4), similar to those of Co^3+^ in ZnCo_2_O_4_. These results suggest that Zn^2+^ and Ga^3+^ substitution did not alter the spinel scaffolds and that Co^2+^_Td_ and Co^3+^_Oh_ were the only forms of the Co^2+^ and Co^3+^ in CoGa_2_O_4_ and ZnCo_2_O_4_, respectively.

### Catalytic ozone activation is dependent on the physicochemical properties

Catalytic ozonation efficiencies of pristine Co_3_O_4_ and its geometric site substitutes were evaluated using oxalic acid (OA) as the target probe (Supplementary Table 5). OA is highly recalcitrant to the attack by O_3_ (${k}_{{{\rm{o}}}_{3}}\,$= 0.04 M^−1^ s^−1^) and those ROSs with a weak oxidation capacity, such as superoxide radical (O_2_^•–^) and singlet oxygen (^1^O_2_), but can be readily destructed by ^•^OH (*k*_•OH_ = 1.4 × 10^6 ^M^−1^ s^−1^)^33,34^. The adsorption abilities of OA by the as-synthesized spinel oxides were investigated prior to measuring their catalytic ozonation activity (Supplementary Fig. 17). Marginal OA adsorption (<3%) is observed for all the spinel oxides. Chemical drift measurements reveal that the spinel oxides exhibit a similar point of zero charges (pH_pzc_) of 7 (Supplementary Fig. 18), suggesting that the catalysts are protonated at solution pH 3. Although an electrostatic attraction between the surface of spinel oxides and the negatively charged OA molecules (pK_a1_ = 1.2) is expected, the low specific surface areas (SSAs) of the spinel oxides hinder further adsorption (Supplementary Table 6; Supplementary Fig. 19). This electrostatic attraction, however, facilitates the interactions between O_3_ and OA molecules on the catalyst surface in catalytic reactions.

**Fig. 2:**
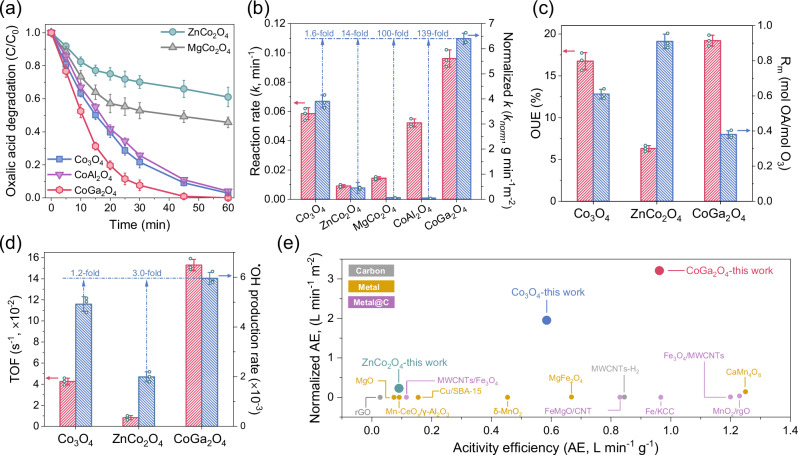
Heterogeneous catalytic ozonation activities of the catalysts. **a** Catalytic ozonation activities of the as-synthesized Co-based spinel oxides for oxalic acid (OA) degradation. Reaction condition: [catalyst] = 0.1 g L^−1^; temperature: 25 °C; initial pH = 3, [OA]_0_: 50 mg L^−1^. **b** Comparison of the reaction rates (*k*s) and BET surface area normalized reaction rates (*k*_*norm*_s) of different spinels/O_3_ systems. **c** Comparison of ozone utilization efficiency (OUE) and R_m_ values of different spinels/O_3_ systems. **d** Comparison of turnover frequency (TOF) and ^⦁^OH production rates of different spinels/O_3_ systems. **e** Comparison of the catalytic ozonation performance of our spinel oxide catalysts with values from previous reports. Catalysts include δ-MnO_2_^62^, CaMn_4_O_8_^63^, MWCNTs^64^, Fe_3_O_4_/MWCNTs^65^, 2% Cu/SBA-15^66^, Mn-CeO_x_/γ-Al_2_O_3_^67^, MgO[111]^68^, MgFe_2_O_4_^69^, MnO_2_/rGO^70^, MWCNTs/Fe_3_O_4_^71^, FeMgO/CNT^72^, Fe/KCC^73^, and rGO^74^. Error bars represent the standard deviations from three replicate measurements. Source data are provided as a Source Data file.

In the catalytic O_3_ activation, geometrical-site-dependent activity is observed. CoGa_2_O_4_ with substituted Co^3+^_Oh_ sites displays a higher performance to the pristine Co_3_O_4_ in OA destruction, achieving over 95% destruction of the initial OA (50 mg L^−1^) within 45 min (Fig. 2a, details on optimizing the catalytic loading/O_3_ dosage and influence of initial solution pH are provided in Supplementary Figs. 20 and 21, respectively). Contrarily, replacing the Co^2+^_Td_ sites by Mg^2+^ and Zn^2+^ drastically decreases the OA degradation efficiency, with ~46 and 61% of OA remaining after 60 min. By normalizing the pseudo-first reaction kinetics (*k*, min^−1^) of the spinel oxides with their SSAs, a tremendous increase (12.4–81.3-fold) in the apparent reaction kinetics (*k*_*norm*_, g min^−1^ m^−2^) is observed for Co^2+^_Td_-dominated CoGa_2_O_4_ compared to those of ZnCo_2_O_4_ and MgCo_2_O_4_ with Co^3+^_Oh_ as the prevailing sites (Fig. 2b; Supplementary Table 7). This suggests that Co^2+^_Td_ sites exhibit a higher activity in O_3_ activation than Co^3+^_Oh_ (Supplementary Fig. 22). Furthermore, the trivial metal leaching of these as-synthesized spinels from inductively coupled plasma mass spectrometry results indicates the robust crystal structures against the highly-oxidative reaction environment created by O_3_ and the produced ROS (Supplementary Table 8). Noted that the high SSA by severe agglomerations of CoAl_2_O_4_ nanoparticles results in a much lower *k*_*norm*_ than that of CoGa_2_O_4_. Additionally, the morphological differences between CoAl_2_O_4_/MgCo_2_O_4_ and other spinels also lead to the exposure of facets with varying reactivities. Therefore, the subsequent investigations primarily focus on Co_3_O_4_ and its geometrical-site substitutes with comparable SSAs and identical exposure of [111] facet (i.e., CoGa_2_O_4_ and ZnCo_2_O_4_).

Considering the low SSAs of the as-synthesized catalysts (~0.3 m^2^ g^−1^) which might not facilitate the exposure of the active sites, we subsequently synthesized three-dimensional ordered macroporous Co_3_O_4_ (3DOM-Co_3_O_4_), plate Co_3_O_4_, and spherical Co_3_O_4_, achieving higher SSAs of 29.1, 17.5, and 78.4 m^2^ g^−1^ accordingly (Supplementary Fig. 23) to investigate the effect of SSAs on catalytic activity. Plate Co_3_O_4_ and spherical Co_3_O_4_ display lower activity than that of CoGa_2_O_4_ (Supplementary Fig. 24), alongside significantly higher cobalt leaching (0.58, 0.66, and 0.09 mg L^−1^ for plate Co_3_O_4_, spherical Co_3_O_4_, and CoGa_2_O_4_, respectively). 3DOM-Co_3_O_4_ obtains a similar activity to CoGa_2_O_4_, yet simply increasing exposure amount of the active sites did not enhance their intrinsic activity. Both the *k*_*norm*_ and the electrochemical surface area (ECSA) normalized reaction kinetics (*k*_*ECSA*_) of 3DOM-Co_3_O_4_ are significantly lower than those of CoGa_2_O_4_ (59-fold and 8.7-fold, respectively) (Supplementary Fig. 25). Similarly, the intrinsic activity (*k*_*ECSA*_s) of Co sites in plate Co_3_O_4_ and spherical Co_3_O_4_ are 13 and 39-fold less than that of CoGa_2_O_4_. These suggest the great significance of regulating coordination geometry in Co-based spinel outperforms SSA engineering in determining the intrinsic activity of the active sites.

The high activity of CoGa_2_O_4_ is accompanied by a 1.15-fold improvement in O_3_ utilization efficiencies (OUE) compared to the pristine Co_3_O_4_ (Fig. 2c; Supplementary Fig. 26). In contrast, substituting the active Co^2+^ with catalytically inactive Zn^2+^ dramatically reduced the OUE. This trend is also discovered in the variations in molar ratios of the degraded OA to the consumed O_3_ (R_m_). The lowest R_m_ value of CoGa_2_O_4_ among the as-synthesized spinel oxides indicates that accelerated conversion kinetics of O_3_ into ROS on Co^2+^_Td_ sites. As a result, the calculated turnover frequency (TOF) (Fig. 2d; Supplementary Table. 9) for CoGa_2_O_4_ with the high exposure of Co^2+^_Td_ sites is 3.5- and 17.6-fold greater than that of Co_3_O_4_ with mixed Co^2+^_Td_/ Co^3+^_Oh_ sites and ZnCo_2_O_4_ with Co^3+^_Oh_-dominant sites (0.15, 0.043, and 0.0082 s^−1^), respectively. In addition, CoGa_2_O_4_ synthesized in this study, despite with small SSAs, are among the highest reported (Fig. 2e; Supplementary Data 1). This high intrinsic activity of CoGa_2_O_4_ can be attributed to the selective exposure of the active Co^2+^_Td_ sites via coordinating environment regulation. Co^2+^_Td_ sites with a greater tendency to donate the frontier electrons facilitate the electrophilic activation of O_3_ molecules than Co^3+^_Oh_ sites^16,21^.

Influence of the exposed crystal facet on catalytic activity was also investigated by synthesizing cubic-shaped Co_3_O_4_ exposing the [100] crystal facet as the reference catalyst (Supplementary Fig. 27). Results suggest that octahedral-shaped Co_3_O_4_ with the [111] crystal facet exhibits a higher intrinsic activity than the cubic-shaped Co_3_O_4_ with the [100] crystal facet. Although the sparse distribution of Co atoms in the [100] facet results in a greater surface defective structure than the [111] facet, the larger number of the active Co^2+^_Td_ sites on the [111] facet governs the catalytic activity (Supplementary Fig. 28; Supplementary Table 10)^35,36^. This further discloses the decisive role of geometric coordination sites in catalytic activity. Furthermore, the inversed Co^2+^_Oh_ sites as the minor coordination-geometry-competitors against the dominant Co^2+^_Td_ sites demonstrate a markedly lower intrinsic catalytic activity than the Co^2+^_Td_ sites, which can be ascribed to the inefficient electron transfer ability of octahedrally coordinated O atoms Co^2+^_Oh_ sites compared to tetrahedrally coordinated O atoms in Co^2+^_Td_ sites (Supplementary Fig. 29)^21^.

Apart from the geometrical sites, surface chemical features and electronic properties of TM oxides might affect O_3_ adsorption and its subsequent activation^37,38^. To disclose the effects of these properties on catalytic ozonation activity, structural-activity relationships are established based on the obtained physicochemical properties from the temperature programmed desorption (TPD), cryo-electron paramagnetic resonance (EPR), and electrochemical impedance spectroscopy (EIS) tests (Supplementary Fig. 30), which were summarized in a heatmap in Supplementary Fig. 31. Although geometric site substitutions alter the surface chemical properties compared to those of pristine Co_3_O_4_, these variations in physicochemical properties do not act as pivotal factors in determining the catalytic activities of the spinel oxides. These results further reinforce the fundamental roles of the unsubstituted host Co cation sites in determining the intrinsic activity for catalytic ozonation.

### Selective formation of ROSs in the catalytic ozonation processes by spinel oxides

**Fig. 3:**
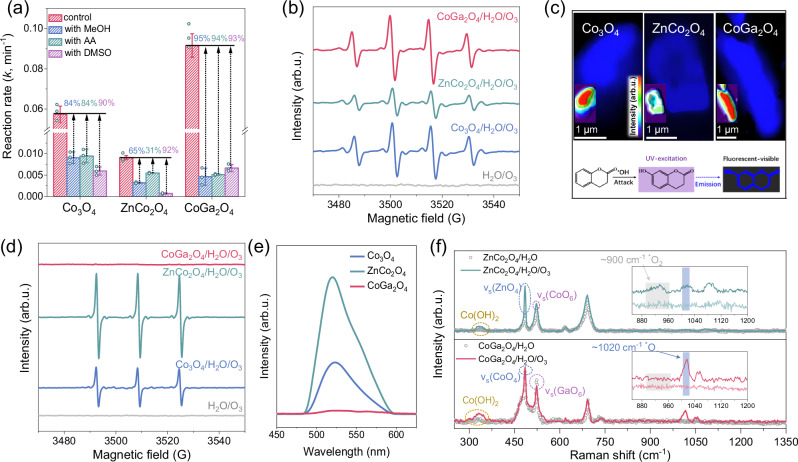
Selective ROSs generation on different Co sites. **a** Comparison of rate constants of quenching tests in different spinels/O_3_ systems. Reaction conditions: [catalyst]: 0.1 g L^−1^; temperature: 25 °C; initial pH = 3, [OA]_0_: 50 mg L^−1^, [methanol, MeOH]_0_: 10 mM, [acetic acid, AA]_0_: 10 mM, [dimethyl sulfone, DMSO]_0_: 1 mM. **b** Electron paramagnetic resonance (EPR) spectra with 5,5-dimethyl-1-pyrroline (DMPO) as the spin trapping agent for ^•^OH detection. **c** Visualization of the generated ^•^OH at the surface of spinel oxides using coumarin as the ^•^OH fluorescence probe by inverted fluorescence microscopy (IFM). **d** EPR spectra with 2,2,6,6-tetramethyl-4-piperidinol (TEMP) as the spin trapping agent for ^1^O_2_ detection. **e** Photoluminescence spectra of singlet oxygen sensor green (SOSG) as the ^1^O_2_ probe. **f** In situ Raman spectra of ZnCo_2_O_4_ and CoGa_2_O_4_. Error bars represent the standard deviations from three replicate measurements. Source data are provided as a Source Data file.

The type of ROSs produced by Co_3_O_4_ and its geometrical-site substitutes were then distinguished by quenching tests and probe-based in situ spectroscopies. The high accuracy and selectivity of these techniques for ROS detection have been verified using the classic homogeneous systems designed to produce single type of ROS (Supplementary Fig. 32). Methanol (MeOH) and acetic acid (AA) which exhibit high reaction kinetics for ^•^OH (*k*_•OH_ = 9.7 × 10^8^ and 8.5 × 10^7 ^M^−1^ s^−1^, respectively)^34^ but are recalcitrant for O_3_ oxidation were employed as the ^•^OH scavengers (Supplementary Figs. 33–35). The presence of these scavengers significantly hindered OA degradation kinetics for Co_3_O_4_, CoGa_2_O_4_, and ZnCo_2_O_4_ (Fig. 3a), suggesting that OA destruction was dominantly driven by the produced ^•^OH. In situ EPR tests proved the generation of ^•^OH by identifying the DMPO–^•^OH signals with an intensity ratio of 1:2:2:1 (Fig. 3b). Furthermore, the intensity order of the DMPO–^•^OH signal for pristine Co_3_O_4_ and its substitutes closely correlated with their activities, solidifying that Co^2+^_Td_ sites stimulated the O_3_ activation to produce a higher amount of ^•^OH than Co^3+^_Oh_ sites. The production of ^•^OH on the surface of spinel oxides was then visualized by employing coumarin as the probe^39^, characterized by fluorescent blue color accumulation on/near the catalysts on inverted fluorescence microscopy (IFM) images. Among the spinels, CoGa_2_O_4_ displayed the highest fluorescence intensity with faint blue halos around its surface (Fig. 3c)^40^. This suggests that the abundant ^•^OH on CoGa_2_O_4_ surface can activate the ambient water molecules into ^•^OH because of its high oxidation capability, which is confirmed by the higher open-circuit potential (OCP) than those of Co_3_O_4_ and ZnCo_2_O_4_ from in situ electrochemical analysis (Supplementary Fig. 36).

The surface interactions of O_3_ with spinel oxides were examined by using dimethyl sulfoxide (DMSO) as the quenching agent. For CoGa_2_O_4_ with exposed Co^2+^_Td_ sites, DMSO addition results in a similar quenching effect to those of ^•^OH scavengers, suggesting that the surface-adsorbed O_3_ on CoGa_2_O_4_ exhibits fast reaction kinetics for ^•^OH evolution and subsequent release into the bulk solution. Adding DMSO to the ZnCo_2_O_4_/O_3_ system resulted in greater inhibition effect than those of ^•^OH scavengers. The weak interactions between O_3_ and the Co^3+^_Oh_ sites on ZnCo_2_O_4_ hampered the swift O_3_ conversion into ^•^OH and might initiate the production of singlet oxygen (^1^O_2_) as the byproducts. The inferior oxidation potential (0.81 V_NHE_) of ¹O_2_ results in sluggish reactions with OA^41^. Singlet oxygen sensor green (SOSG) was employed as a selective probe for ^1^O_2_ detection (Fig. 3d). For ZnCo_2_O_4_, a strong fluorescence signal at 525 nm was observed, suggesting the formation of a large amount of ^1^O_2_^42^. This fluorescent intensity decreased for pristine Co_3_O_4_ and vanished for CoGa_2_O_4_ with exposed Co^2+^_Td_ sites, indicating that negligible or no ^1^O_2_ was formed on the spinels with exposed Co^2+^_Td_ sites. Results of the in situ EPR tests with 2,2,6,6-tetramethyl-4-piperidinol (TEMP) as the spin trapping agent displayed a similar trend to that of the SOSG tests (Fig. 3e), with ZnCo_2_O_4_ displaying a high signal intensity compared to the negligible intensity observed for CoGa_2_O_4_. The above results suggested that Co^2+^_Td_ sites exhibit a strong selectivity for ^•^OH generation while Co^3+^_Oh_ sites can induce both ^•^OH and ^1^O_2_ production.

In situ Raman tests reveal a strong signal at ~1020 cm^−1^ in the CoGa_2_O_4_/O_3_ system, which can be ascribed to the produced surface-adsorbed single O atoms (*O) stemming from the catalytic dissociation of the O_3_ on the Co^2+^_Td_ sites (Fig. 3f). Moreover, the CoGa_2_O_4_/O_3_ system obtains a higher signal intensity at ~300 cm^−1^ than the pristine CoGa_2_O_4_ without a reaction^43^, suggesting that a large amount of surface-bound ^•^OH were formed during the catalytic ozonation process. In contrast, the lower Raman signals of 1020 and 300 cm^−1^ observed in the ZnCo_2_O_4_/O_3_ system than those in the CoGa_2_O_4_/O_3_ system reveal that less *O and surface-bound ^•^OH are produced because of the weak interaction between the Co^3+^_Oh_ sites and O_3_. Additionally, a peak corresponding to the surface-adsorbed O_2_ species (*O_2_) at ~900 cm^−1^ emerges in the ZnCo_2_O_4_/O_3_ system, indicating that side reactions occur on the Co^3+^_Oh_ sites. We further evaluate the variations of Raman peaks for the stretching vibrations of the Co^2+^_Td_ (~486 cm^−1^) and Co^3+^_Oh_ (~525 cm^−1^) sites after ozone introduction, which were originated from the stretching of the Co atoms by complexing with O_3_ and the subsequent geometrical-site-involved lattice interactions. Clearly, introducing O_3_ to CoGa_2_O_4_ induced less variations in the geometrical sites-involved stretching vibrations than those of ZnCo_2_O_4_. This suggests a robust bonding behavior within the lattice frameworks and a minimal change in the surface chemical environment after Ga substitution.

The selectivity of the Co^2+^_Td_ and Co^3+^_Oh_ sites on ^•^OH production was assessed by the ^•^OH/O_3_ conversion rates. Compared to the catalytically inactive ZnCo_2_O_4_, the highly active CoGa_2_O_4_ obtained a 3.0-fold increase in ^•^OH/O_3_ conversion rate (Fig. 2d), confirming the high selectivity of Co^2+^_Td_ sites towards ^•^OH production. The production of ^1^O_2_ as the byproduct ROS might stem from the distinct O_3_ adsorption and intermediate evolution routes on Co^3+^_Oh_ sites, which require higher electron consumption and result in a decreased O_3_ utilization efficiency.

### Origin of the ozone-metal interaction and the pathways of ROSs formation and evolution

**Fig. 4:**
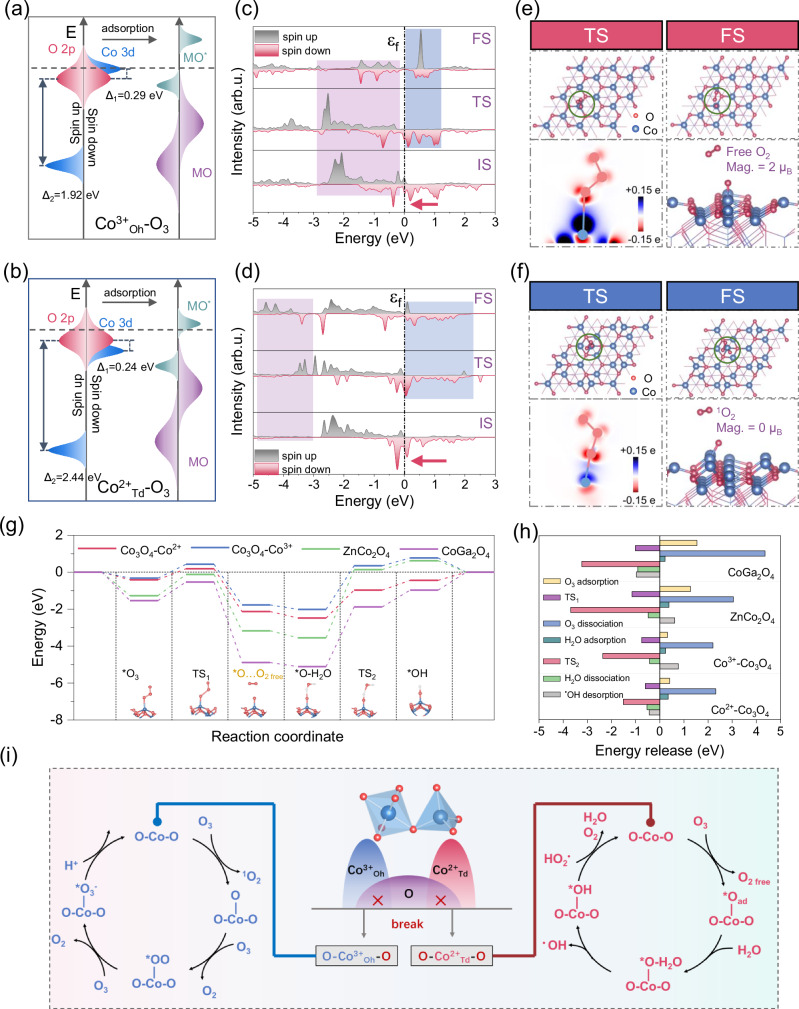
Mechanism investigation for activity and selectivity differences. Schematic illustration of molecular orbital formation between Co^2+^_Td_ (**a**)/Co^3+^_Oh_ (**b**) sites and O_3_ molecules based on the projected electronic density of states (PDOS) profiles on Co *3d* orbitals and the corresponding *d*-band center analysis. PDOS profiles of O_3_ adsorption at the Co_3_O_4_-Co^2+^_Td_ site (**c**) and the Co_3_O_4_-Co^3+^_Oh_ site (**d**) of initial state (IS), transition state (TS), final state (FS). O_3_ adsorption configurations at TS and FS and the corresponding charge density difference (CDD) plots at the Co_3_O_4_-Co^2+^_Td_ site (**e**) and the Co_3_O_4_-Co^3+^_Oh_ site (**f**). Relative energy profiles and the simplified surface structures of the various reaction species for reaction pathway over spinel oxides (**g**) and the corresponding computed energy releases between different transition states (**h**). **i** Proposed reaction process for ^•^OH generation on different Co sites. At Co^2+^_Td_ sites, decomposition of O_3_ generates O_2_ and surface-adsorbed O atom (^*^O_ad_) with a high oxidation potential, which oxidizes water to generate ^•^OH. In contrast, Co^3+^_Oh_ sites preferentially produce ^1^O_2_, while the remaining ^*^O_ad_ with a low oxidation potential fails to further react with water to generate ^•^OH. Source data are provided as a Source Data file.

To illustrate the intrinsic electronic states of the spinel oxides and hybridization strength between Co *3d* orbitals and O *2p* orbitals from O_3_ molecules and their influences on activity, we constructed Co_3_O_4_, CoGa_2_O_4_, and ZnCo_2_O_4_ models with the same exposure of [111] facet, based on the deciphered results from XAS and superconducting quantum interference device magnetization analysis (Supplementary Figs. 37 and 38; Supplementary Table 11), for use in the density functional theory (DFT) calculations. As revealed in the crystal orbital Hamilton population (COHP) analysis, the strong covalency competition arising from the substitution of Ga^3+^ or Zn^2+^ into crystal structure crystal favors the exposure of the unsubstituted cations Co^2+^_Td_ and Co^3+^_Oh_ as the preferentially exposed active sites in CoGa_2_O_4_ and ZnCo_2_O_4_^12^ (Supplementary Fig. 39). The calculation outcomes well match the XAS analysis results, supporting the validity of the constructed models. Given the electron-rich nature of O_3_ molecules, all molecular orbitals (MOs) and partial of anti-bonding molecular orbitals (MO*s) can be occupied when O_3_ hybridizes on either Co^2+^_Td_ or Co^3+^_Oh_ sites (Supplementary Fig. 40), making the occupancy of MO*s crucial to determine the electron transfer resistance and hybridization strength^44^. Projected electronic density of states (PDOS) profiles on Co *3d* orbitals and the corresponding *d*-band center analysis reveal that the high spin polarization resulting from the covalency competition positions the band centers of spin-down channels (*ɛ*_*d*↓_) of the spinel oxides at higher energy levels than those of spin-up channels (*ɛ*_*d*↑_), endowing spin-down channels a decisive role in communicating with the O_3_ *2p* orbitals for MO*s formation (Supplementary Fig. 41). Based on the orbital analysis results, orbital overlapping diagrams are plotted (Fig. 4a, b). MO*s formed by hybridization of spin-down channel of Co^2+^_Td_ sites and O_3_ *2p* orbitals (MO*s- Co^2+^_Td_) are situated closer to the Fermi level than those formed by Co^3+^_Oh_ sites. This facilitates the electron transition at Co^2+^_Td_ sites and decreases the energy barriers for O_3_ dissociation, which promotes the production of reactive intermediates^45^.

The interaction of O_3_ molecules with the exposed Co^2+^_Td_ or Co^3+^_Oh_ sites during the ozone activation process results in distinctly different electronic states. O_3_ adsorption (transition state, TS) and dissociation (final state, FS) on Co^2+^_Td_ sites result in the movement a larger portion of electrons across the Fermi level (*ɛ*_*f*_) than those on Co^3+^_Oh_ sites (Fig. 4c, d), confirming the higher occupancy of MO*s- Co^2+^_Td_ than those formed by Co^3+^_Oh_ orbitals. Charge density difference (CDD) calculations reveal that the terminal O atom in O_3_ is more favorable to abstracting electrons from the Co^2+^_Td_ site than the Co^3+^_Oh_ site (Fig. 4e), which aligns well with the results of Bader charge analyses (Supplementary Figs. 42–45). Consequently, a stronger chemisorption between Co^2+^_Td_ and O_3_ than that of Co^3+^_Oh_ is achieved, providing the adsorption energies of 0.41 and 0.32 eV, respectively. The higher bond order between Co^2+^_Td_ *3d* orbital and O *2p* orbital than that of Co^3+^_Oh_ (2 vs. 1.5) (Supplementary Fig. 46) further verifies the greater hybridization strength at the Co^2+^_Td_ sites, which promotes the electronic communications. The magnetic moments for O_2 free_ generated on Co^2+^_Td_ and Co^3+^_Oh_ sites are calculated as 2 and 0 μ_B_ accordingly, corresponding to the normal O_2_ molecule and the ^1^O_2_ molecule, respectively. This agrees with the results of ROS identification, suggesting the selectivity of the geometrical sites in ROS production. Bader charge analysis reveals that the O_3_ dissociation into ^1^O_2_ on Co^3+^_Oh_ sites requires a greater number of electrons than on Co^2+^_Td_ sites, which explains the low OUE and ^•^OH production rate on Co^3+^_Oh_ sites.

O_3_ activation for ^•^OH evolution on spinel oxides mainly involves four elementary reactions^46,47^. (1) O_3_ adsorption on the active site to form surface-adsorbed O_3_ (*O_3_); (2) catalytic dissociation of *O_3_ to form a surface-adsorbed O atom (*O_ad_) and a free O_2_ molecule (O_2 free_); (3) interaction of *O_ad_ with the ambient H_2_O molecule (*O_ad_^…^H_2_O); and (4) cleavage of the adsorbed H_2_O molecule for ^•^OH production (*OH^… •^OH). In both TSs for *O_ad_ formation (TS_1_) and surface-confined ^•^OH production (TS_2_), which govern the tendency for O_3_ dissociation and ^•^OH evolution accordingly, Co^2+^_Td_ sites obtain lower energy barriers than Co^3+^_Oh_ sites (Fig. 4f, g). Additionally, the energy released during both O_3_ and H_2_O dissociation steps is greater on Co^2+^_Td_ sites than Co^3+^_Oh_ sites. These further solidify that MO*s-Co^2+^_Td_ sites near the Fermi level facilitate the electron injection, enhancing the reaction thermodynamics for catalytic O_3_ dissociation and ^•^OH formation. Previous studies have reported that the *O_ad_ attached to active sites after O_3_ dissociation can also bond with another O_3_ for ^•^OH generation^48^ (Fig. 4h). However, this ^•^OH evolution route is complicated and requires higher O_3_ and electron consumptions, making it thermodynamically unfavorable compared to the direct H_2_O oxidation (Supplementary Fig. 47).

### Practical application potentials of the spinel-induced catalytic ozonation process

**Fig. 5:**
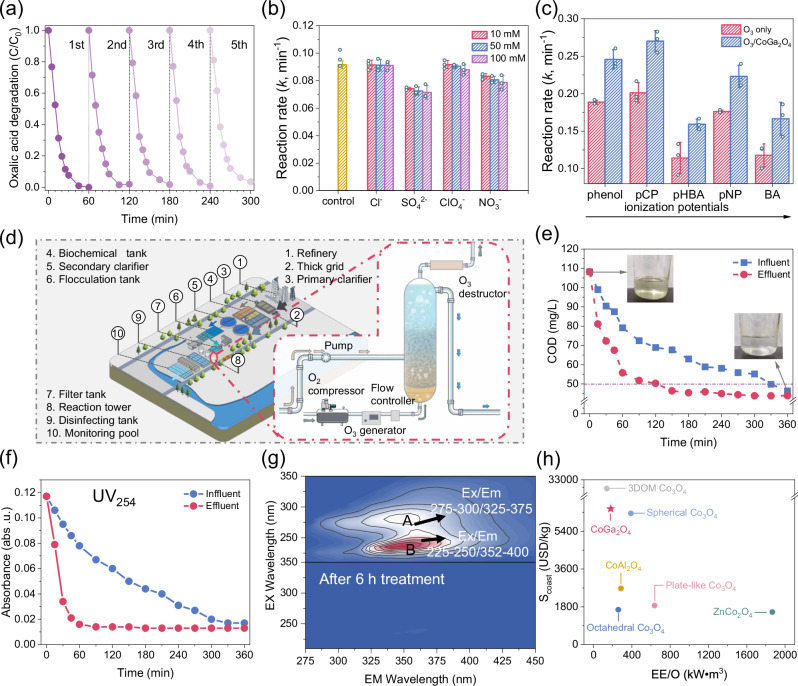
Practical application potentials. **a** Cyclic experiments for the CoGa_2_O_4_/O_3_ system. **b** Influences of inorganic anions on the catalytic performance for the CoGa_2_O_4_/O_3_ system. **c** Comparisons of reaction rate constants for different phenolics. Pollutants include phenol, para-chlorophenol (pCP), para-nitrophenol (pNP), para-hydroxyl benzoic acid (pHBA), benzoic acid (BA). **d** Schematic diagram of the proposed catalytic ozonation system using the continuous-flow reactor. O_3_ generated by the pressurized O_2_ was introduced into the fixed-bed reactor (with catalyst immobilized on the polyurethane foam) from the bottom diffusor with its flow rate regulated by a mass-flow rate controller. Simultaneously, the wastewater to be treated was fed above the O_3_ diffuser. After the reaction, the remaining O_3_ gas exiting from the top of the reaction column went through an ozone decomposer before being discharged, while the treated reaction solution was collected directly. **e** Chemical oxygen demand (COD) removal profiles for treatment of real refinery wastewater in the continuous-flow reactor. **f** UV_254_ variation profiles for the treatment of real refinery wastewater. **g** 3D-Excitation-emission-matrix (EEM) spectra raw and treated wastewater. Ex excitation wavelength, Em emission wavelength. Reaction condition: ozone flow rate 100 mL min^−1^, ozone concentration: 25 mg L^−1^, catalyst loading: 1 g L^−1^, wastewater flow rate: 48 mL min^−1^_._ **h** Comparison of electrical energy per order (EE/O) and synthesis costs for different catalytic ozonation systems. Error bars represent the standard deviations from three replicate measurements. Source data are provided as a Source Data file.

CoGa_2_O_4_ with the highest catalytic ozonation activity owing to the maximized exposure of Co^2+^_Td_ was employed to evaluate the application potentials. Cyclic experiments reveal that CoGa_2_O_4_ exhibits a high reusability, maintaining over 95% of its initial catalytic activity after 5 cycles (Fig. 5a; Supplementary Fig. 48). In contrast, both Co_3_O_4_ and ZnCo_2_O_4_ were partially deactivated after multiple runs. XPS deconvolution results on O 1s spectra suggest that ZnCo_2_O_4_ demonstrated a more significant change in the peaks related to -OH/OV for the fresh/used samples than that of the CoGa_2_O_4_ (Supplementary Fig. 49). In addition, O_2_-TPD profiles further reveal that the area of the OV peak at 200–300 °C decreases remarkably for the used ZnCo_2_O_4_, yet minor changes are observed for the fresh/used CoGa_2_O_4_. Therefore, the high surface rigidity with marginal metal leaching (0.3 wt.%) accounts for the superior stability of CoGa_2_O_4_.

Influences of inorganic anions on catalytic performance were then evaluated. The presence of inorganic anions with the concentrations between 10 and 100 mM induces minor inhibitions in activities (Fig. 5b; Supplementary Fig. 50). It is suggested that surface-bounded ^•^OH produced by the strong hybridization between Co^2+^_Td_ and O_3_ via the inner-sphere complexation accounts for the high adaptation towards inorganic anions. In contrast, the weak interaction between Co^3+^_Oh_ sites and O_3_ resulted in the outer-sphere complexation, which can be heavily interfered with by ionic strength (Supplementary Fig. 51). We also investigated the catalytic ozonation performance of CoGa_2_O_4_ to degrade various recalcitrant phenolic contaminants (Fig. 5c; Supplementary Figs. 52 and 53). Compared to single ozonation process, the CoGa_2_O_4_/O_3_ system achieved both high reaction rates and total organic carbon (TOC) mineralization rates for the treatment of phenolics with different ionization potentials^49^. This can be ascribed by the high OUE and ^⦁^OH production efficiency CoGa_2_O_4_ via the efficient electron transfer between Co^2+^_Td_ and O_3_.

To further evaluate the practical application potential of CoGa_2_O_4_/O_3_ system, CoGa_2_O_4_ was immobilized on cleaned, defatted cotton in a continuous-flow fixed-bed column reactor (0.25 L working volume, with residence time of 5 min) to treat the air flotation effluent wastewater of a petrochemical plant in southern China with initial COD of 108 mg L^−1^ (Fig. 5d; Supplementary Fig. 54). The detailed properties of the real wastewater are listed in Supplementary Table 12. The effluent COD decreases to lower than 50 mg L^−1^ (with a removal efficiency of 46%) within 90 min treatment and reaches 41 mg L^−1^ after 6 h treatment with a corresponding TOC value of 20 mg L^−1^ (Fig. 5e). These meet the Discharge Standard of Pollutants for Petroleum Refining Industry in China (GB 31570-2015, COD < 60 mg L^−1^, TOC < 20 mg L^−1^). Additionally, the time-dependent variation profile of UV_254_ indicates that the CoGa_2_O_4_/O_3_ system achieves an effective removal of aromatic contaminants within 90 min of the treatment (Fig. 5f). Furthermore, 3D-EEM fluorescence spectra suggests that two major fluorescence peaks centered at A (Ex/Em maxima at 275–300/325–375 nm) and B (Ex/Em maxima 225–250/352–400 nm) are discerned in the untreated petrochemical wastewater (Fig. 5g), attributed to aromatic N-containing compounds and petroleum-derived dissolved organic matters (DOM), respectively^50^. After treatment, the peaks in the 3D-EEM spectra nearly disappeared, indicating the exceptional ability of the CoGa_2_O_4_/O_3_ system to destroy a broad band refractory organic contaminants within the real petrochemical wastewater.

Economic evaluations on the catalyst synthesis costs and the EE/O (electrical energy per order) suggest that the cobalt-based spinels synthesized in this study have a great advantage on technological economy than the other types of Co_3_O_4_-based catalysts (Fig. 5h; Supplementary Fig. 55; Supplementary Data 2; and Supplementary Table 13). For future applications, Ga^3+^ can be replaced by more economical metals. Facile treatments, such as defect engineering and heteroatom doping, can also be applied to the cobalt-containing spinels. The optimized ambient microenvironments surrounding the active Co^2+^_Td_ sites can enhance the activity by inducing more intense electronic communications and stronger complexation with the O_3_ molecules, which thereby trigger the increased production of surface-bound ^•^OH that are recalcitrant to inorganic anions in wastewater.

## Discussion

In summary, a geometrical-site-dependent activity was discovered in catalytic ozone activation on Co_3_O_4_ and its Co^2+^_Td_ and Co^3+^_Oh_ substitutes at the [111] crystal facet. CoGa_2_O_4_ with Co^2+^_Td_-dominated sites demonstrated a 17.6-fold higher TOF than that of ZnCo_2_O_4_ with high exposure of Co^3+^_Oh_ sites, which can be ascribed to the improved O_3_ utilization efficiency and the enhanced ^•^OH generation selectivity by avoiding the formation of unfavorable ^1^O_2_ accounted on Co^2+^_Td_ sites. Theoretical calculations suggested that the highly spin-polarized Co^2+^_Td_ sites fine-tuned the position of the MO*s-Co^2+^_Td_, formed by spin-down channels of Co *3d* orbital, close to the Fermi level, decreasing the energy barriers for O_3_ dissociation and accelerating the electronic communication with the O *2p* orbitals of the adsorbed O_3_ molecules. The strong hybridization strength with the promoted electron transfer between Co^2+^_Td_ sites and O_3_ molecules induced inner-sphere complexation and favored the formation of surface-bound ^•^OH. As a result, the CoGa_2_O_4_/O_3_ system exhibited high adaptability towards inorganic anions and demonstrated high treatment efficiency for real petrochemical wastewater. Tailoring the spin-polarized electronic states from geometrical-site engineering offers intrinsic insights into regulating the reaction thermodynamics in TM oxide-induced catalysis. Moreover, the modulated ROS production by different geometrical sites envisaged the practical potential of spinel oxides in real wastewater treatment.

## Methods

### Chemicals

Sulfuric acid (H_2_SO_4_, 98%), cobalt chloride hexahydrate (CoCl_2_⦁6H_2_O, 99.99%), zinc chloride anhydrous (ZnCl_2_, 99.95%), aluminum chloride hexahydrate (AlCl_3_⦁6H_2_O), magnesium chloride (MgCl_2_, 99.95%), gallium chloride (GaCl_3_, 99.99%), citric acid (CA, 99.5%), ethanol (99%), methanol (MeOH, 99%), para-chlorophenol (pCP, 99.5%), para-nitrophenol (pNP, 99.9%), phenol (99.9%), para-hydroxyl benzoic acid (pHBA, 99.5%), benzoic acid (BA, 99.9%), DMSO (99%), methylene blue (MB, 96%), rhodamine B (95%), OA (99.8%), sodium hydroxide (NaOH, 99%), and sodium sulfate (Na_2_SO_4_, 99%) were obtained from Aladdin Reagents. Horseradish peroxidase (95%) was procured from Sigma-Aldrich. 2,2,6,6-tetramethyl-4-piperidinol (TEMP, 99%) and 5,5-dimethyl-1-pyrroline (DMPO, 99%) were purchased from Dojindo Laboratories. SOSG was purchased from Invitrogen by Thermo Fisher Scientific. Ultra-pure water (18.2 MΩ⦁cm) was used in all the experiments. All the chemicals in this work were used for further purification.

### Synthesis of geometrical-substituted cobalt-based catalysts

A sol-gel method was used to synthesize cobalt-based spinel oxides^51^. For synthesis of CoGa_2_O_4_, ZnCo_2_O_4_, and MgCo_2_O_4_, stoichiometric amounts of CoCl_2_•6H_2_O, GaCl_3_, ZnCl_2_, and MgCl_2_ (Co/Ga = 1/2 for CoGa_2_O_4_, Co/Zn = 2/1 for ZnCo_2_O_4_, and Co/Mg = 2/1 for MgCo_2_O_4_) were mixed in a citric acid solution (18 mL, 1.5 M) with a molar ratio of 1:1.5 under magnetic stirring at room temperature to form a stable homogeneous solution. The resulting mixed solution was vigorously stirred while heated in a water bath at 80 °C until a highly viscous gel was formed. The gel was subsequently dried overnight at 120 °C in an oven for foaming. The dried mixture was then milled to a powder and calcined at 700 °C in a muffle furnace for 3 h with a heat ramp of 5 °C/min. Pristine Co_3_O_4_ without geometrical-site substitution was prepared using the same synthesis protocol with CoCl_2_•6H_2_O as the precursor. For CoAl_2_O_4_ synthesis, a similar synthesis protocol was adopted with the CoCl_2_•6H_2_O and AlCl_3_•6H_2_O as the metal precursors (CoCl_2_•6H_2_O/AlCl_3_•6H_2_O molar ratio of 1/2), except for the final calcination temperature was changed to 1000 °C.

### Characterization

XRD patterns of the as-synthesized materials were recorded to investigate their crystallinity and phases (X’Pert-PROMPD, PAN analytical B.V. with Cu Kα radiation). Morphologies of the samples were observed using a ZEISS Gemini SEM 300 with an accelerating voltage of 5 kV. Moreover, transmission electron microscopy (TEM) images, high-angle annular dark field scanning TEM (HAADF-STEM) images, and corresponding energy-dispersive X-ray spectroscopy (EDX) elemental mappings were obtained from a Talos F200S TEM/STEM equipped with an energy-dispersive spectrometer (EDS, SUPERX). The X-ray absorption structure (XAS) spectra, including XANES and extended X-ray absorption fine structure (EXAFS) for the as-synthesized cobalt-based spinels at Co *K*-edge and Ga *K*-edge were collected at the Beamline 21A in National Synchrotron Radiation Research Center, Taiwan, China. The surface chemistry of the materials was investigated using the X-ray photoelectron spectroscopy (XPS) under ultra-high vacuum conditions with Al-Kα X-ray irradiation (Thermo Fisher Scientific ESCALAB 250Xi). N_2_ sorption isotherms were measured using a Micrometrics Tristar 3000 to determine the SSAs and pore size distribution. Raman spectra were recorded using an ISA argon-laser Raman spectrometer (LabRAM HR Evolution) with a wavelength of 532 nm. Temperature-dependent magnetizations were conducted under a magnetic field (*H* = 1 kOe) by the following field-cooling procedures (400–5 K) by a vibrating sample magnetometer (Lakeshore 7404). The point of zero charge (pH_pzc_) was measured by the chemical drift method^52^. Probe-based in situ electron paramagnetic resonance (EPR) tests for ROS detection were conducted by a Bruker EMX-PLUS EPR spectrometer. An additional detailed description of characterization methods is provided in Supplementary Note 1.

### Ozone activation and catalytic ozonation

Catalytic ozonation degradation of aqueous organics was performed in a semi-batch glass reactor at 25 °C. High-purity oxygen (99.999%) was a flow rate of 100 mL min^−1^ was used for ozone generation (Anseros, Germany), and the concentration of the generated O_3_ was monitored by an online ozone detector (Anseros Ozomat GM, Germany). The generated ozone was introduced into the reaction solution through the glass diffusor at the bottom of the semi-batch reactor. Unless specified, the reaction solution was prepared by adding 0.05 g of catalyst to a 500 mL solution containing 50 mg L^−1^ of OA. The initial pH of reaction solution was adjusted to 3 by 0.01 M H_2_SO_4_/NaOH in OA solution. At certain time intervals, samples were withdrawn from the reaction solution, filtered by a 0.22 μm PES filter, and injected into sample bottles for the subsequent ultra-high-performance liquid chromatography (UPLC, Thermo Fisher U3000) and mineralization of TOC (total organic carbon, Shimadzu TOC-CPH) analysis. In the stability evaluation of the catalyst after multiple runs, the catalyst was recovered by vacuum filtration after each cycle, washed three times with ultra-pure water, and dried at 60 °C in the oven for the next use. The dissolved O_3_ in the reaction solution was measured through the Indigo blue method^53^. H_2_O_2_ in solution was examined by the horseradish peroxidase method^54^. A detailed description of the analysis methods is provided in Supplementary Note 2.

### Ozone utilization efficiency calculations

The ozone utilization efficiency can be calculated by the following equation:

1 $$\text{OUE}=\frac{{[{\text{O}}_{3}]}_{\text{used}}}{{[{\text{O}}_{3}]}_{\text{input}}}=\frac{{\int }_{0}^{{\text{t}}}{\left[{\text{O}}_{3}\right]}_{\text{in}}\text{dt}-{\int }_{0}^{{\text{t}}}{\left[{\text{O}}_{3}\right]}_{\text{offgas}}\text{dt}-{\int }_{0}^{{\text{t}}}{\left[{\text{O}}_{3}\right]}_{\text{solution}}\text{dt}}{{\int }_{0}^{\text{t}}{\left[{\text{O}}_{3}\right]}_{\text{in}}\text{dt}}$$

where, [O_3_]_used_ is the total ozone utilization, [O_3_]_input_ is the input ozone concentration, [O_3_]_solution_ is the dissolved ozone concentration, [O_*3*_]_offgas_ is the exhaust gas concentration after the ozone reaction.

The *R*_O_ factor (molar ratios of the degraded OA to the consumed O_3_) can be calculated by the following equations.

2 $${{\text{n}}[\mathrm{OA}]}_{\mathrm{degraded}}={{\text{n}}[\mathrm{OA}]}_{0}-{{\text{n}}[\mathrm{OA}]}_{t}$$

3 $${{\text{R}}}_{\text{O}}=\frac{{{\text{n}}[\text{OA}]}_{\text{degraded}}}{{\text{n}}{[{\text{O}}_{3}]}_{\text{used}}}$$

where *n*[OA]_degraded_ is the molar amount oxalic acid degraded, ${{\text{n}}[\mathrm{OA}]}_{0}$ is the oxalic acid concentration at the initial moment, ${{\text{n}}[OA]}_{t}$ is the residual oxalic acid concentration after the reaction, and ${\text{n}}{[{{\rm{O}}}_{3}]}_{\mathrm{consumed}}$ represents the molar amount of consumed O_3_.

### Calculation of S_BET_ normalized activity efficiency

S_BET_ normalized activity efficiency (AE), which can be derived from the following equations, was used to compare the catalytic performances of the as-synthesized Co-based spinels with the reported catalysts in literature.

4 $${\text{AE}}=\frac{{\text{Reaction}} \,{\text{rate}}}{{\text{catalyst}}\, {\text{loading}}}$$

5 $${\text{Normalized}} \,{\text{AE}}=\frac{\mathrm{AE}}{{S}_{\mathrm{BET}}\,}$$

### In situ Raman experiments

In situ Raman spectra were collected by a laser confocal Raman spectrometer (LabRAM HR Evolution HORIBA, Japan) equipped with a 532 nm laser. Typically, 10 mg of the sample was placed in an in situ reaction cell. Saturated O_3_ solution with pH 3 was continuously pumped into the reaction cell with a flow rate of 15 mL L^−1^ to ensure the catalyst was uniformly permeated.

### Calculation of ^•^OH concentration

The concentration of the produced ^•^OH during the reaction was quantified using OA as the molecular probe due to its sluggish reaction rate with the produced ^1^O_2_ and ROS with low oxidation potentials.

6 $$-\frac{\text{d}\left[\text{OA}\right]}{\text{dt}}={{\text{k}}}_{{\text{O}}_{3}}\left[\text{OA}\right]\left[{\text{O}}_{3}\right]+{k}_{{{\scriptstyle{{\bullet }}\atop}} {\mathrm{OH}}}[\text{OA}][{\scriptstyle{{\bullet }}\atop} \!\mathrm{OH}]$$

7 $${\mathrm{ln}}\left(\frac{[{\mathrm{OA}}]}{[{\mathrm{OA}}]_{0}}\right)=-({{\text{k}}}_{{\text{O}}_{3}}{\int }_{0}^{{\text{t}}}{\left[{{\rm{O}}}_{3}\right]}_{\mathrm{sol}}{\text{dt}}+{k}_{{{\scriptstyle{{\bullet }}\atop}} {\mathrm{OH}}}{\int }_{0}^{{\text{t}}}[{\scriptstyle{{\bullet }}\atop} \!\mathrm{OH}]\text{dt})$$

8 $${\int }_{0}^{{\text{t}}}[{\scriptstyle{{\bullet }}\atop} \!\mathrm{OH}]\text{dt}=-(\mathrm{ln}\left(\frac{[\mathrm{OA}]}{{[\text{OA}]}_{0}}\right)+{{\text{k}}}_{{\text{O}}_{3}}{\int }_{0}^{{\text{t}}}{\left[{{\rm{O}}}_{3}\right]}_{\mathrm{sol}}\text{dt})/{k}_{{{\scriptstyle{{\bullet }}\atop}} {\mathrm{OH}}}$$

Where ${k}_{{{\rm{O}}}_{3}}$ is the rate constant for OA with ozone, ${k}_{\bullet \mathrm{OH}}$ is rate constant for OA with ^•^OH radicals, ${\left[{{\rm{O}}}_{3}\right]}_{\mathrm{sol}}$ is the dissolved O_3_ concentration in the reaction solution, and [^*•*^OH] is the concentration of ^•^OH.

Correspondingly, the ^•^OH/O_3_ conversion rate (*R*•_OH_) can be calculated by the following equation.

9 $${\text{R}}=\frac{{\int }_{0}^{{\text{t}}}[{\scriptstyle{{\bullet }}\atop} \!\mathrm{OH}]\text{dt}}{{\int }_{0}^{{\text{t}}}{\left[{{\rm{O}}}_{3}\right]}_{\mathrm{sol}}\text{dt}}$$

### In situ electrochemical measurements

In situ electrochemical measurements were conducted on an electrochemical workstation (CHI 760D, CH Instrument) with a graphite electrode, a saturated calomel electrode, and a glassy carbon electrode (GCE) as the counter electrode, reference electrode, and working electrode, respectively. The electrolyte was prepared by 0.1 M Na_2_SO_4_ solution. H_2_SO_4_ and NaOH solutions (both 0.1 M) were employed to adjust the electrolyte pH to 3. For the preparation of the working electrode, 20 mg of the catalysts were dispersed in 1 mL of isopropanol containing 20 μL of Nafion® solution. The mixed solution was ultrasonicated for 1 h to form a homogeneous ink. The GCE surface was coated by 10 μL ink drops, which were then air-dried and subsequently heated in an oven at 80 °C for 5 min. The working electrode was stabilized in the electrolyte overnight to ensure a stable potential prior to each electrochemical test. In situ OCP tests were performed after stabilization of spinel-catalysts-GCE in the electrolyte. O_3_ was introduced to the reactor with a flow rate of 30 mL min^−1^ and a concentration of 25 mg L^−1^ and the change in potential was recorded. Electrochemical impedance spectroscopy (EIS) is measured in the frequency range of 0.01 Hz to 100 kHz with an amplitude of 10 mV at the open-circuit potential (OCP) of each material.

### Density functional theory calculations

DFT calculations were performed using the Vienna ab initio simulation package, version 5.4.1^55–57^. The exchange-correlation energy was calculated using the Perdew−Burke−Erzenhof (PBE) functional^58^. The Hubbard *U* parameter (GGA +*U*) with *U* = 4.0 eV was adopted to accurately calculate the electron correlation within the d states of cobalt ions. The total energy change during the structure optimization process ultimately converged to 5 × 10^−6^ eV. In addition, the forces per atom were reduced to 0.01 eV Å^−1^. The k-point mesh of the Brillouin zone was set at 2 × 2 × 1 for geometry optimization. A cutoff energy of 450 eV was employed during the computations. The detailed information of the constructed DFT models is presented in Supplementary Table 14. In the calculations, the bottom atom layer was remained fixed, while the other atoms were allowed to relax^59,60^. The climbing image nudged elastic band (CI-NEB) method was used to determine the transition state of the reaction. The charge distribution within a specific structure was determined through static self-consistent calculations. Based on these results, local atomic charges were computed using the Bader charge analysis method. The crystal orbital Hamilton populations (COHPs) of spinel oxides were calculated using the computer program Local Orbital Basis Suite Toward Electronic-Structure Reconstruction (LOBSTER)^61^. And the computed energy releases in different transition states are provided in Supplementary Table 15.

The formation energy of the adsorbate is calculated using the following formula:

10 $${\Delta {\text{E}}}_{\text{ads}}={{\text{E}}}_{\text{slab}-\text{adsorbate}}-{{\text{E}}}_{\text{slab}}-{{\text{E}}}_{\text{adsorbate}}$$

where the *E*_surface-adsorbate_, *E*_slab,_ and *E*_adsorbate_ are the total energies of the adsorbed state, clean surface, and adsorbate, respectively.

## Supplementary information

Supplementary Information

Description of Additional Supplementary Files

Supplementary Data 1

Supplementary Data 2

Transparent Peer Review file

## Source data

Source Data

## Data Availability

The data supporting the findings of this study are included within the main text and the Supplementary Information file. Source data are provided with this paper as a Source Data file. All the raw data relevant to the study are available from the corresponding author upon request. Source data are provided with this paper.
