# Supplementary material for: Tailored ozone activation on geometrical-site-dependent cobalt with selective coordination
Source: Nat Commun. 2025 Jul 1;16:5921. doi: 10.1038/s41467-025-61181-7 (PMC12214642; doi:10.1038/s41467-025-61181-7)
Supplement: Supplementary file 1 — Supplementary Information [file 41467_2025_61181_MOESM1_ESM.pdf]

Supplementary Information

for

Tailored ozone activation on geometrical-site-dependent cobalt with selective coordination

Shenning Liu[1], Yuxian Wang[1,*], Ya Liu[2], Peihan Chen[1], Tao Kong[1], Xiaoguang Duan[2], Chunmao Chen[1,*], Hongqi Sun[3,*], Shaobin Wang[2]

[1] State Key Laboratory of Heavy Oil Processing, China University of Petroleum-Beijing, Beijing 102249, China.

[2] School of Chemical Engineering, The University of Adelaide, Adelaide, SA 5005, Australia

[3] School of Molecular Sciences, The University of Western Australia, Crawley, WA 6009, Australia

*Corresponding Authors

E-mail address: yuxian.wang@cup.edu.cn (YW)

c.chen@cup.edu.cn (CC)

hongqi.sun@uwa.edu.au (HS)

Supplementary Notes

Supplementary Note 1. Characterization details

Cryo-electron paramagnetic resonance (EPR) tests were performed at -196 °C and maintained by liquid nitrogen, with the following test conditions: sweep time of 60 s, microwave power of 1.00 mW, modulation amplitude of 2 G, modulation frequency of 100.00 kHz, and time constant of 5.12 ms.

Temperature programmed desorption by O_2 (O_2-TPD, Xianquan TP5080) was examined to study oxygen species on the surface of the catalysts. For the O_2-TPD test, a certain amount of a catalyst was pretreated under a He flow of 50 mL min^{-1} at 300 °C for 1 h and cooled down to 50 °C. O_2-TPD spectra (10% O_2-90% He) were recorded from 50 to 700 °C at a heating rate of 10 °C min^{-1}.

Temperature programmed desorption by NH_3 (NH_3-TPD, Micromeritics AutoChem II 2920) was examined to study acidic site distribution on the catalyst surface. For the NH_3-TPD test, a certain amount of a catalyst was pretreated under a He flow of 30 mL min^{-1} at 300 °C for 1 h and cooled down to 50 °C. NH_3-TPD spectra (10% NH_3-90% He) were recorded from 50 to 700 °C at a heating rate of 10 °C min^{-1}.

Probe-based in situ electron paramagnetic resonance (EPR) tests for ROS detection were conducted by a Bruker EMX-PLUS EPR spectrometer. A suspension containing 0.1 g L^{-1} catalyst and saturated O_3 solution at pH of 3 reacted for 10 min with sufficient mixing. A certain amount of suspension was filtrated and immediately mixed with DMPO (250 mM) or TEMP (250 mM) and tested in an EPR spectrometer (Bruker EMX-PLUS). During the tests, tert-butanol as the $^{\bullet}OH$ quencher was added in TEMP solution to neutralize its interference. Test conditions: sweep time, 60 s; microwave power, 20 mW; field modulation amplitude, 2 G; center field, 3510 G; sample g factor: 2.0000.

Supplementary Note 2. Analysis methods related to the catalytic ozonation tests

The concentrations of OA and different phenolic contaminants were determined by ultrahigh-performance liquid chromatography (UPLC, Thermofisher U3000 series, USA) with an Acclaim 120 C18 column (2.1 ×150 mm, 5 μm). UHPLC detecting parameters for different organics and their ionization potentials are listed in **Supplementary Table 5**.

The dissolved ozone concentration was detected by the Indigo method[1]. Specifically, the experiment was conducted in a 0.5 semi-batch glass flask reactor containing 0.05 g catalyst at 25 °C. At certain intervals, 1 mL of the reaction mixture was withdrawn from the reactor and promptly filtered through a 0.22 μm PTFE filter. The filtrate was then thoroughly mixed with 4 mL of indigo solution prepared in phosphate buffer for 30 seconds. The absorbance of the resulting mixture was measured at 600 nm. The concentration of ozone in the liquid phase can be calculated via the following equation.

$$C=\frac{2.4\times(A_0-A)\times V_0}{V} \quad (1)$$

where, C (mg L^{-1}), liquid phase ozone concentration; A_0, blank sample absorbance; A, sample absorbance; V_0 sample volume; V sampling reaction solution volume.

Photoluminescence spectra spectroscopy was used to further verify the presence of •OH using coumarin as the fluorescence probe. The experiment was conducted in a 0.5 semi-batch glass flask reactor containing 0.05 g catalyst at 25 °C. After 10 min of reaction, 3 mL of the sample was extracted from the spinel oxides/ozone system and promptly mixed with 1 mL of coumarin solution (5 mM). Subsequently, roughly 1 mL of this mixture was transferred to a glass slide for subsequent image acquisition and analysis. An inverted fluorescence microscope was employed to capture images. The setting parameters are UV excitation at 330–380 nm, color separation at 400 nm, and cut-off at 420 nm. The exposure time was set

as 20 s for all the captured images in this work.

A commercial fluorescence probe-SOSG was used to detect generated $^1O_2$ in the spinels/O_3 system. 100 μg SOSG was dissolved 300 μL Ar purged methanol to prepare the SOSG solution. Before initiating the catalytic ozonation reaction, 40 μL of the prepared SOSG solution was added into the reaction solution. After 10 min of reaction, 5 mL solution was withdrawn and immediately filtered by a 0.22 μm PES syringe filter for fluorescence analysis (F-7000, Hitachi) with an excitation wavelength of 488 nm and an emission wavelength of 500-650 nm.

The TOF for a single active site is the quantity of conversions per unit of time. The catalyst intrinsic activity is represented by the TOF value, which quantifies the rate of catalytic reaction.

$$\text{TOF}=\frac{\textit{The number of react converted of product produced}}{\textit{Reaction time}\times\textit{the number of active sites}} \tag{2}$$

$$\text{The number of active sites} = SSA \times D \tag{3}$$

$$\text{D}=\frac{\text{atom number}}{S_{(111)}}=\frac{1}{\left(\frac{\sqrt{2}}{2}a\right)^2\sin\frac{\pi}{3}}=\frac{4\sqrt{3}}{3}a^2 \tag{4}$$

where, “the number of react converted of product produced” represents the conversion rate of $^{\bullet}OH$; reaction time is 3600 s; SSA is specific surface areas; D is the degree of (111) crystal facets atomic concentration; and a is the lattice constant obtained from the result of Rietveld-refined XRD.

Supplementary Table 1. XRD Rietveld refinement results and comparisons of the interplanar spacing and bond length in Co_3O_4, $ZnCo_2O_4$, and $CoGa_2O_4$.

Sample	Rietveld refinement results (Å)			d (Å, Bragg's law)	d (Å, interplanar spacing equation)
	a	b	c		
Co_3O_4	8.10	8.10	8.10	4.74	4.72
$ZnCo_2O_4$	8.08	8.08	8.08	4.73	4.70
$CoGa_2O_4$	8.33	8.33	8.33	4.85	4.82

Supplementary Table 2. Deconvolution results for high-resolution XPS surveys of O 1s and Co 2p for the as-synthesized samples.

Catalyst	Co 2p		O 1s		
	Co^{2+}(at.%)	Co^{3+}(at.%)	Lattice O(at.%)	-OH/OV (at.%)	Adsorbed O (at.%)
Co_3O_4	33.1	66.9	41.2	43.4	15.4
$ZnCo_2O_4$-fresh	0	100	40.6	37.7	21.7
$ZnCo_2O_4$-used	0	100	55.3	20.8	23.9
$CoGa_2O_4$-fresh	100	0	44.7	31.8	23.5
$CoGa_2O_4$-used	100	0	55.3	29.3	15.4

Supplementary Table 3. The EXAFS fitting parameters at the Co K-edge for various samples.

**Sample**	**Shell**	**N**	**R(Å)**	**σ^2(Å)**	**ΔE_0(eV)**	**S_0^2**	**R factor**
	Co-O	6	1.93	0.006(1)			
Co_3O_4	Co-Co1	6	2.92	0.004(1)	-3.23	0.77	0.017
	Co-Co2	12	3.40	0.006(1)			
	Co-O	4	1.95	0.005(1)			
$ZnCo_2O_4$	Co-Co	12	2.94	0.004(1)	-2.72	0.85	0.015
	Co-Zn	12	3.42	0.006(1)			
	Co-O	4	2.06	0.005(1)			
$CoGa_2O_4$	Ga-Ga	6	3.01	0.007(1)	1.72	0.79	0.024
	Co-Co	12	3.52	0.006(1)			

Supplementary Table 4. The EXAFS fitting parameters at the Ga K-edge for $CoGa_2O_4$.

Sample	Shell	N	R(Å)	σ^2(Å)	ΔE_0(eV)	S_0^2	R factor
$CoGa_2O_4$	Ga-O	6	1.95	0.0098(1)	4.959	0.83	0.023
	Ga-Ga	6	2.96	0.0066(1)			
	Ga-Co	6	3.46	0.0029			
	Ga-Ga	6	3.57	0.0018(1)			

Supplementary Table 5. UHPLC detecting parameters for different organics and their ionization potentials.

**Organics**	**Flow rate (mL)**	**Mobile phase (methanol: phosphoric acid (2 mM))**	**Wavelength (nm)**	**pKa**	**Ionization Potential (ev)**
Oxalic acid	0.80	80: 20	210	1.14, 4.32	—
Phenol (Ph)	0.25	50: 50	215	9.95	8.49
4-Nitrophenol (pNP)	0.25	70: 30	254	7.15	9.10
para-Chlorophenol (pCP)	0.25	70: 30	280	9,18	8.69
Benzoic acid (BA)	0.25	65: 35	230	4.21	9.50
para-Hydroxyl benzoic acid (pHBA)	0.25	65: 35	254	4.48	8.79

Supplementary Table 6. BET specific surface area and pore information of the as-synthesized samples.

Sample	BET Surface Area (m^2 g^{-1})	Pore Volume (cm^3 g^{-1})	Average pore size (nm)
Co_3O_4	0.3	0.0034	4.4
$ZnCo_2O_4$	0.4	0.0084	7.7
$MgCo_2O_4$	4.5	0.0043	45.8
$CoAl_2O_4$	23.1	0.16	26.9
$CoGa_2O_4$	0.3	0.0071	7.6
Plate Co_3O_4	17.5	0.053	12.1
Spherical Co_3O_4	78.4	0.283	14.4
3DOM-Co_3O_4	28.1	0.204	29.1

Supplementary Table 7. Observed rate reaction constants (k), BET surface area and catalysts load normalized apparent reaction rate (k_{norm}) and R_O of the as-synthesized samples.

Sample	k (min^{-1})	k_{norm} (g m^{-2} min^{-1})	O_3 consumption (mole OA/mole O_3)
Co_3O_4	0.0585	3.8	1.64
$ZnCo_2O_4$	0.0091	0.42	1.10
$CoGa_2O_4$	0.0962	5.2	2.63
$MgCo_2O_4$	0.0144	0.064	-
$CoAl_2O_4$	0.0522	0.046	-

Supplementary Table 8. The concentration of Co element in the solution after reaction with different spinel catalysts.

Sample	Cobalt concentration (mg/L)
Co_3O_4	0.28
$ZnCo_2O_4$	0.25
$CoGa_2O_4$	0.08
$CoGa_2O_4$ after 4th usage	0.09

Supplementary Table 9. Turnover frequencies (TOFs) of ozone conversion from different spinel oxides.

Sample	$\int[O_3]dt$ (mol)	$\int[^{\cdot}OH]dt$ (mol)	a (Å)	D	TOF
Co_3O_4	5.2×10^{-4}	2.5×10^{-6}	8.10	1.0×10^{16}	4.3×10^{-2}
$ZnCo_2O_4$	1.8×10^{-4}	3.5×10^{-7}	8.078	7.2×10^{15}	8.2×10^{-3}
$CoGa_2O_4$	5.6×10^{-4}	5.3×10^{-6}	8.33	5.8×10^{15}	1.5×10^{-1}

Supplementary Table 10. Proportion of Co^{3+} and Co^{2+} on different exposed facets of Co_3O_4 in previous reports

No.	Catalyst	Co^{2+} (%)	Co^{3+} (%)	Ref.
1	[100]-Co_3O_4-1	44.99	55.01	[2]
	[111]-Co_3O_4-1	57.05	42.95	
2	[100]-Co_3O_4-2	33.44	66.56	[3]
	[111]-Co_3O_4-2	33.47	66.53	
3	[100]-Co_3O_4-3	33.56	66.44	[4]
	[111]-Co_3O_4-3	36.50	63.50	
4	[100]-Co_3O_4-4	51.22	48.78	[5]
	[111]-Co_3O_4-4	52.15	47.85	
5	[100]-Co_3O_4-5	66.23	33.77	[6]
	[111]-Co_3O_4-5	67.11	32.89	
6	[100]-Co_3O_4-6	35.6	64.4	[7]
	[111]-Co_3O_4-6	42.8	57.2	
7	[100]-Co_3O_4-7	36.22	63.67	[8]
	[111]-Co_3O_4-7	42.19	57.81	
	[100]-Co_3O_4-9	31.7	68.3	This work
	[111]-Co_3O_4-9	33.1	66.9	

Supplementary Table 11. Curie-Weiss fitting parameters and fitting results of the as-prepared cobalt oxides samples.

**Sample**	**1/C**	**C**	μ_{eff}	**Co**$^{2+}$**-**t_2	**Co**$^{3+}$**-**e_g
Co_3O_4	0.319	3.13	5.01	3.327	0.96
$ZnCo_2O_4$	0.706	1.42	3.36	-	0.94
$CoGa_2O_4$	0.594	1.69	3.67	3.38	-

Supplementary Table 12. The physico-chemical properties of real wastewater.

Parameter	Value
pH	7.3
COD	107.9 mg/L
UV_{254}	0.117
Cl^-	922.8 mg/L
SO_4^{2-}	793.0 mg/L
NO_3^-	132.3 mg/L
Na^+	474.7 mg/L
NH_4^+	34.3 mg/L
K^+	10.5 mg/L
Mg^{2+}	18.9 mg/L
Ca^{2+}	65.2 mg/L

Supplementary Table 13. Comparison of energy consumption for simulated wastewater using different spinel oxides based on EE/O concept.

Catalysts	Loading ($g\ L^{-1}$)	Initial OA concentration (mg/L)	System power (kW)	Simulated wastewater volume (mL)	Removal rate (C_f/Ci)
Co_3O_4	0.1	50	0.2	500	0.0286
$ZnCo_2O_4$	0.1	50			0.61061
$CoGa_2O_4$	0.1	50			0.07633
3DOM Co_3O_4	0.1	50			0.03553
Plate Co_3O_4	0.1	50			0.23559
Spherical Co_3O_4	0.1	50			0.09414

Supplementary Table 14. Details of DFT models.

Model-exposed site	Periodic unit size	Layers of model	Vacuum slab size	Crystallographic plane	Total charge	Magnetic moment
Co_3O_4-Co^{2+}	a = 9.804 Å, b = 9.804 Å, c = 8.35 Å, α = 60°, β = 120°, and γ = 90°.	2	10 Å	(111)	612	Co^{2+}_{Td}=3, Co^{3+}_{Oh}=2
Co_3O_4-Co^{3+}	a = 9.805 Å, b = 9.805 Å, c = 8.345 Å, α = 60°, β = 120°, and γ = 90°.	2	10 Å	(111)	612	Co^{2+}_{Td}=3, Co^{3+}_{Oh}=2
$ZnCo_2O_4$-Co^{3+}	a = 9.92Å, b = 9.92 Å, c = 8.426 Å, α = 60°, β = 120°, and γ = 90°.	2	10 Å	(111)	648	Zn^{2+}_{Td}=0, Co^{3+}_{Oh}=2
$CoGa_2O_4$-Co^{2+}	a = 10.292 Å, b = 10.292 Å, c = 8.937 Å, α = 60°, β = 120°, and γ = 90°.	2	10 Å	(111)	468	Co^{2+}_{Td}=3, Ga^{3+}_{Oh}=0

Supplementary Table 15. Computed energy releases in different transition states.

Configuration	O_3 adsorption	TS_1	O_3 dissociation	H_2O adsorption	TS_2	H_2O dissociation	•OH desorption
Co_3O_4-Co^{2+}	0.41 eV	-0.6 eV	2.32 eV	0.35 eV	-1.51 eV	-0.53 eV	-0.44 eV
Co_3O_4-Co^{3+}	0.32 eV	-0.75 eV	2.2 eV	0.24 eV	-2.36 eV	-0.42 eV	0.77 eV
$ZnCo_2O_4$-Co^{3+}	1.27 eV	-1.15 eV	3.05 eV	0.38 eV	-3.69 eV	-0.48 eV	0.62 eV
$CoGa_2O_4$-Co^{2+}	1.54 eV	-1.01 eV	4.36 eV	0.22 eV	-3.23 eV	-0.91 eV	-0.97 eV

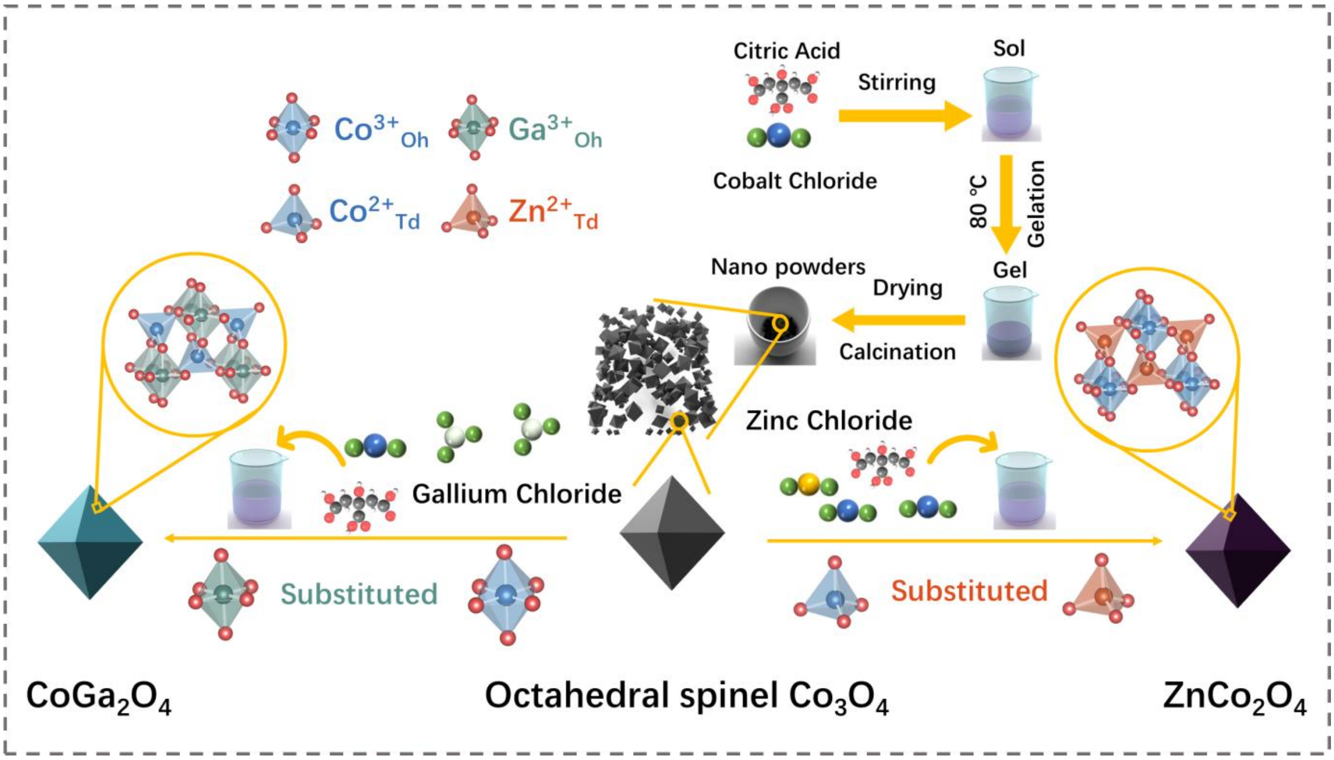

Supplementary Fig. 1 | Synthesis route for Co_3O_4, $ZnCo_2O_4$, and $CoGa_2O_4$ spinel oxides.

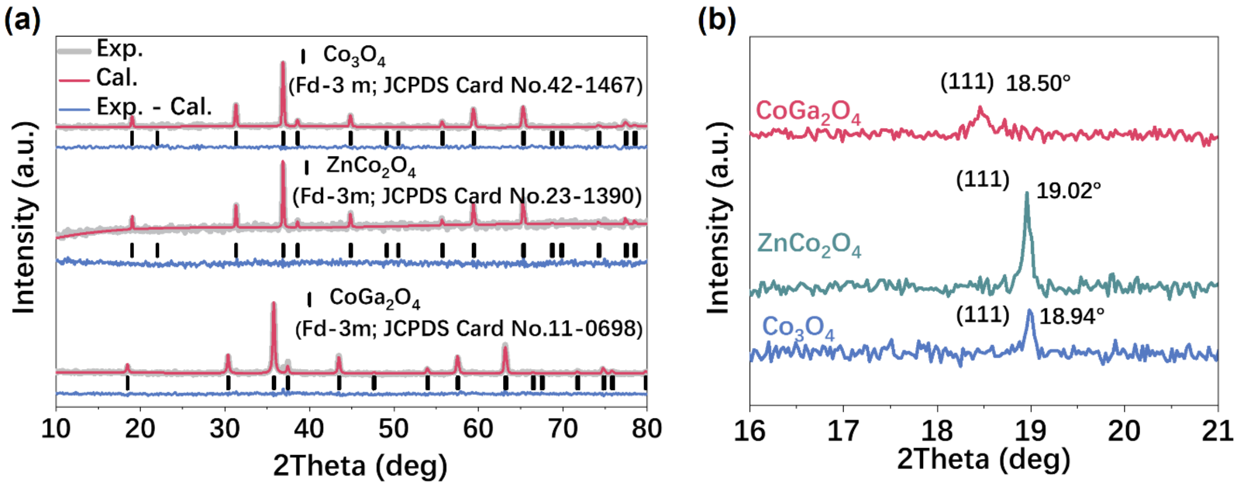

Supplementary Fig. 2 | Crystal structure of the as-synthesized spinels. XRD patterns **(a)** and enlarged XRD patterns **(b)** for Co_3O_4, $ZnCo_2O_4$, and $CoGa_2O_4$.

For pristine Co_3O_4, the diffraction peaks located at 19°, 31°, 37°, 45°, 59°, and 65° are attributed to the (111), (220), (311), (400), (511), and (440) planes of its cubic spinel phase, respectively. $ZnCo_2O_4$ and $CoGa_2O_4$ obtained similar diffraction patterns to pristine Co_3O_4, with peaks slightly shifted to lower positions, suggesting that they shared the same cubic spinel crystal phase.

According to Bragg's law, the interplanar spacing for the (111) plane of Co_3O_4, $ZnCo_2O_4$, and $CoGa_2O_4$ are determined as 4.74, 4.73, and 4.85 Å, respectively. It can be found that substituting Co^{3+} by Ga^{3+} slightly increased the interplanar spacing for the (111) plane, while replacing Co^{2+} by Zn^{2+} trivially decrease the interplanar spacing

We also applied the following equations to rigorously calculate the interplanar spacing of (111) plane for Co_3O_4, $ZnCo_2O_4$, and $CoGa_2O_4$[9].

$$|d|^2=(\mathbf{ha^*}+\mathbf{kb^*}+\mathbf{lc^*})\cdot(\mathbf{ha^*}+\mathbf{kb^*}+\mathbf{lc^*})$$
$$=\mathbf{h}^2|\mathbf{a}^*|^2+\mathbf{k}^2|\mathbf{b}^*|^2+\mathbf{l}^2|\mathbf{c}^*|^2+2\mathbf{hk}|\mathbf{a}^*||\mathbf{b}^*|\cos\gamma^* \quad (5)$$

$$+2\mathbf{hl}|\mathbf{a}^*||\mathbf{c}^*|\cos\boldsymbol{\beta}^*+2\mathbf{kl}|\mathbf{b}^*||\mathbf{c}^*|\cos\boldsymbol{\alpha}^*$$

where **h**, **k**, **l** are the Miller indexes, and **a***, **b***, **c***, cos**α***, cos**β***, and cos**γ*** are constants derived from the unit cell parameters from XRD Rietveld refinement results. The derived interplanar spacings are summarized in the **Supplementary Table 1**. Similarly, the interplanar spacings of (111) plane in $CoGa_2O_4$ was slightly higher than those in Co_3O_4 and $ZnCo_2O_4$.

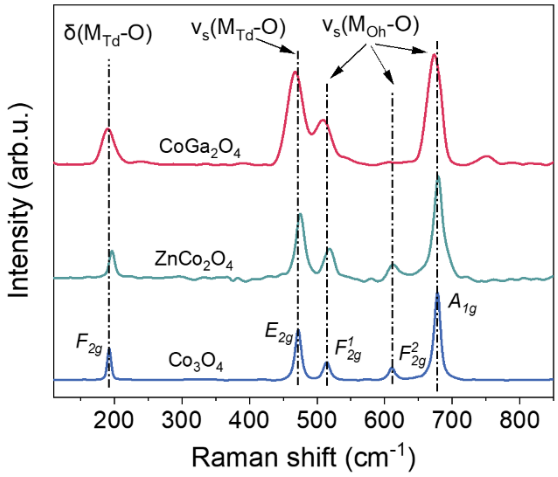

Supplementary Fig. 3 | Raman spectra of spinel oxides.

Raman spectra indicate that Co_3O_4 displays five Raman peaks: F_{2g}^{1} (194 cm^{-1}), E_g (480 cm^{-1}), F_{2g}^{2} (521 cm^{-1}), F_{2g}^{3} (615 cm^{-1}), and A_{1g} (688 cm^{-1}). Specifically, the A_{1g} and F_{2g}^{1} peaks correspond to signals from octahedral and tetrahedra sites, respectively, while E_g, F_{2g}^{2}, and F_{2g}^{3} are signals of octahedral and/or tetrahedra sites without clear identification[10]. Substituting Co^{3+} by Ga^{3+} in $CoGa_2O_4$ blue shifted the A_{1g} symmetry, while the peak position of F_{2g}^{1} symmetry remained unchanged. The variation of the cation-anion bond length at the octahedral sites and polyhedral distortion occurring in the spinel lattice after Ga^{3+} replacement accounts for the change of A_{1g} symmetry in $CoGa_2O_4$. The unchanged coordinative environment of $Co^{2+}{}_{Td}$ sites maintained the F_{2g}^{1} symmetry. Similarly, the shift of F_{2g}^{1} symmetry peak and the maintaining of A_{1g} symmetry in $ZnCo_2O_4$ suggest that Zn^{2+} occupied the tetrahedral sites while the $Co^{3+}{}_{Oh}$ sites remained unchanged. The above Raman results further solidify the normal spinel structures of the as-synthesized Co-based spinels and the minor presence of inversed $Co^{2+}{}_{Oh}$/$Co^{3+}{}_{Td}$ sites[11].

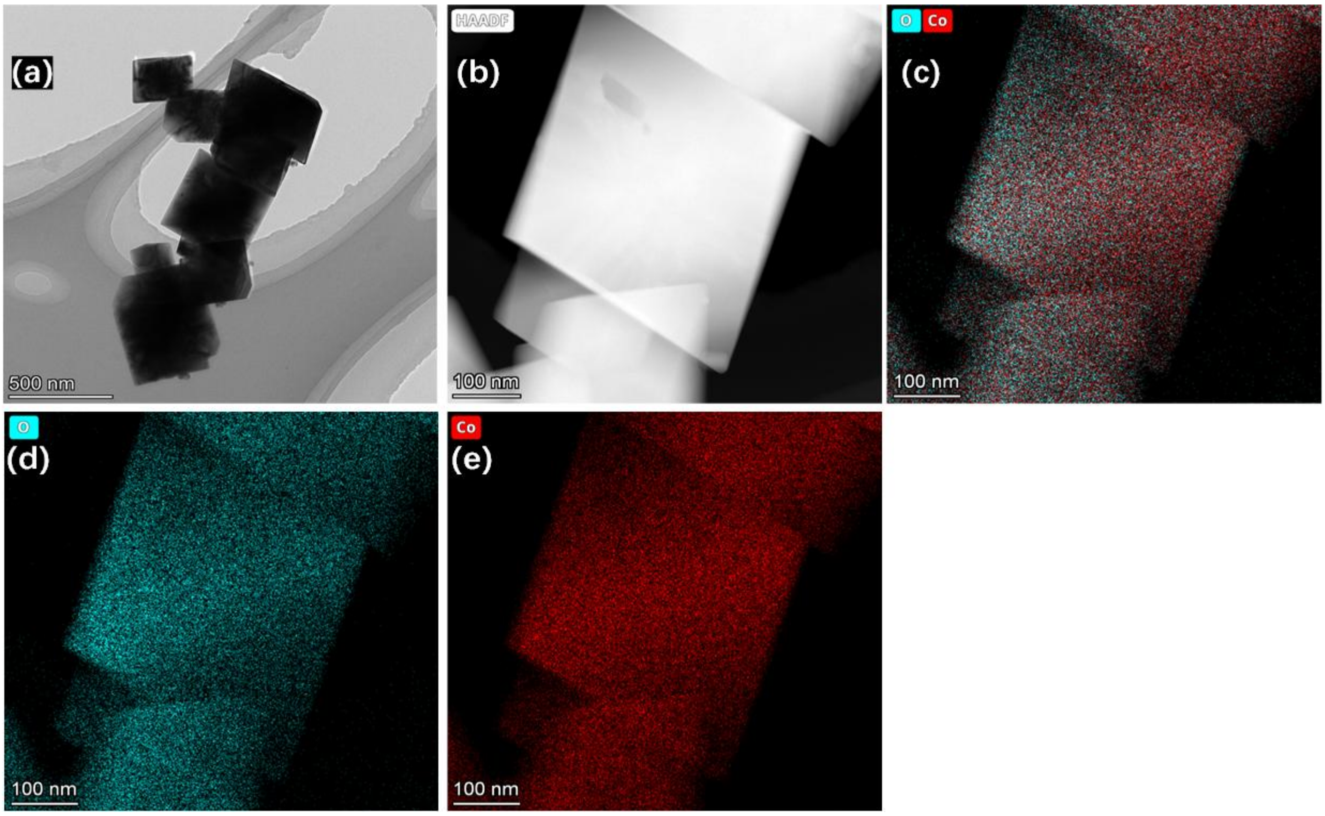

Supplementary Fig. 4 | Microstructure and elemental composition of Co_3O_4. (a) TEM image of Co_3O_4; **(b)** HAADF-STEM image of Co_3O_4 and the corresponding EDX elemental mappings of O and Co **(c)**, O **(d)**, and Co **(e)**.

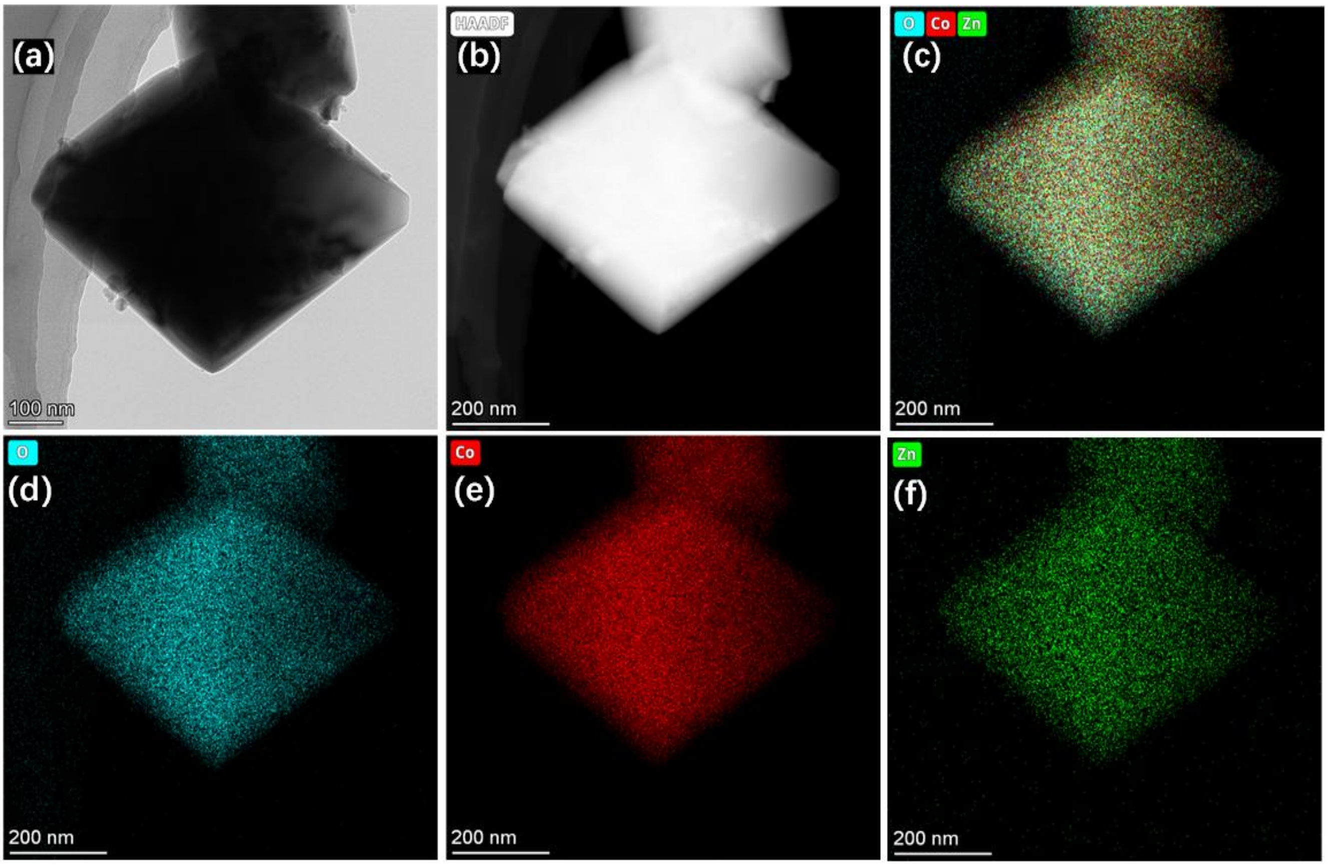

Supplementary Fig. 5 | Microstructure and elemental composition of $ZnCo_2O_4$. (a) TEM image of $ZnCo_2O_4$; **(b)** HAADF-STEM image of $ZnCo_2O_4$ and the corresponding EDX elemental mappings of O, Co, and Zn **(c)**, O **(d)**, Co **(e)**, and Zn **(f)**.

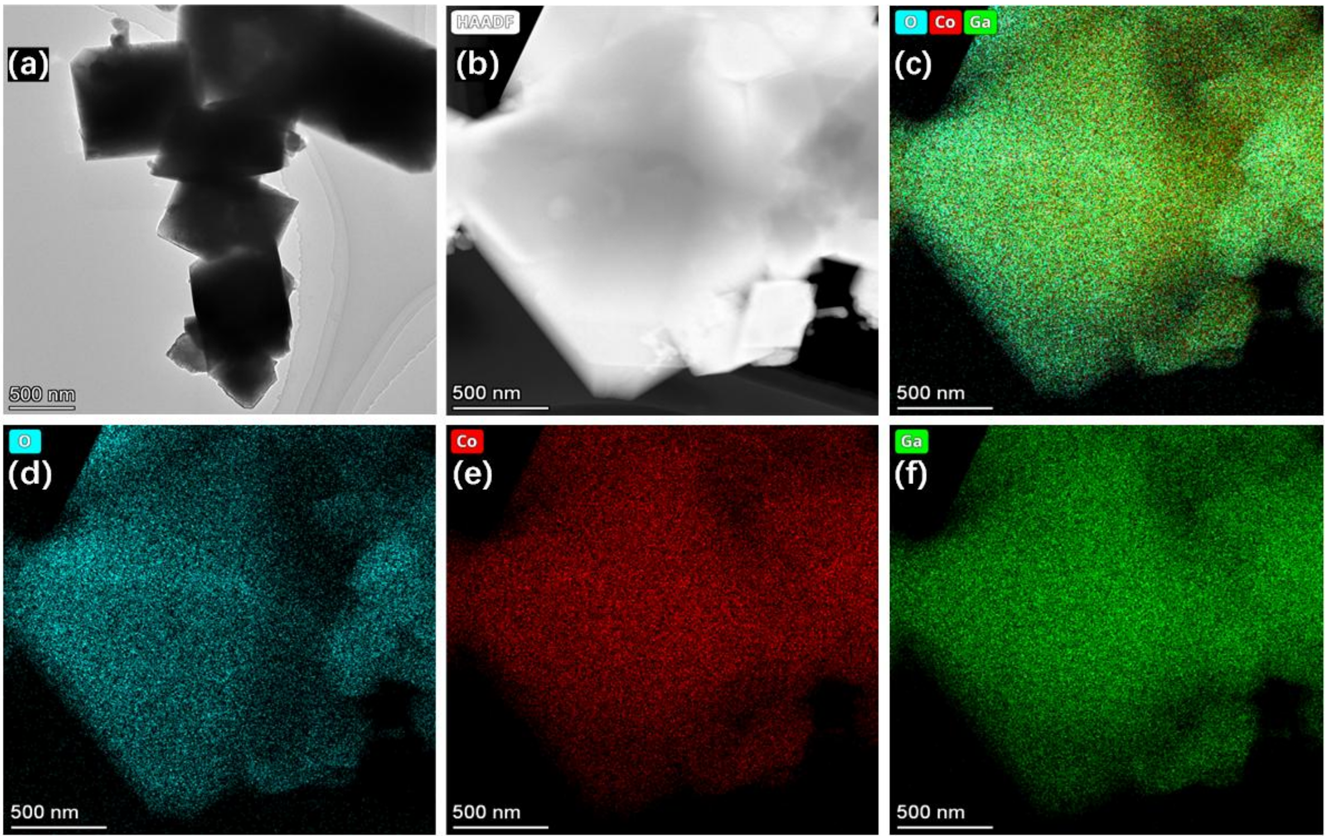

Supplementary Fig. 6 | Microstructure and elemental composition of $CoGa_2O_4$. (a) TEM image of $CoGa_2O_4$; **(b)** HAADF-STEM image of $CoGa_2O_4$ and the corresponding EDX elemental mappings of O, Co, and Ga **(c)**, O **(d)**, Co **(e)**, and Ga **(f)**.

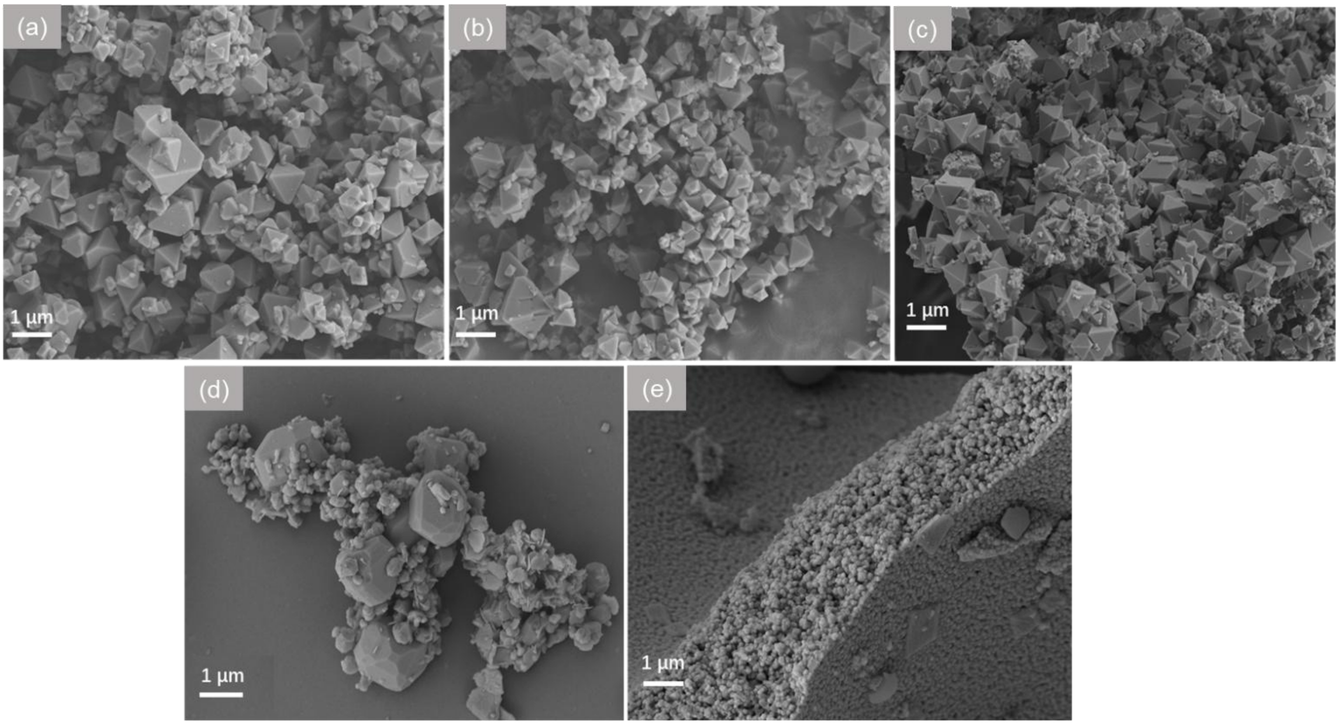

Supplementary Fig. 7 | Morphology of the as-synthesized spinels. SEM images of **(a)** Co_3O_4; **(b)** $ZnCo_2O_4$; **(c)** $CoGa_2O_4$; **(d)** $MgCo_2O_4$; **(e)** $CoAl_2O_4$;

The morphologies of $CoAl_2O_4$ and $MgCo_2O_4$ deviate significantly from those of Co_3O_4, $ZnCo_2O_4$, and $CoGa_2O_4$ due to the differences in ionic radii and charge density. The ionic radii of Co^{2+}, Co^{3+}, Mg^{2+}, Zn^{2+}, Al^{3+}, and Ga^{3+} are 74.5, 61, 72, 74, 53.5 and 62 pm, respectively[12, 13, 14, 15, 16]. The similar ionic radii of Ga^{3+}/Zn^{2+} and Co^{3+}/Co^{2+} enable $CoGa_2O_4$ and $ZnCo_2O_4$ to maintain the same octahedral morphology as Co_3O_4. In contrast, the smaller ionic radius of Al^{3+} compared to Co^{3+} induces contraction in the electronic structure, resulting in the granular morphology of $CoAl_2O_4$. Although Mg^{2+} has an ionic radius similar to that of Co^{2+}, the fewer valence electrons in Mg^{2+} compared to Co^{2+} lead to the flake and block morphology exhibited by $MgCo_2O_4$.

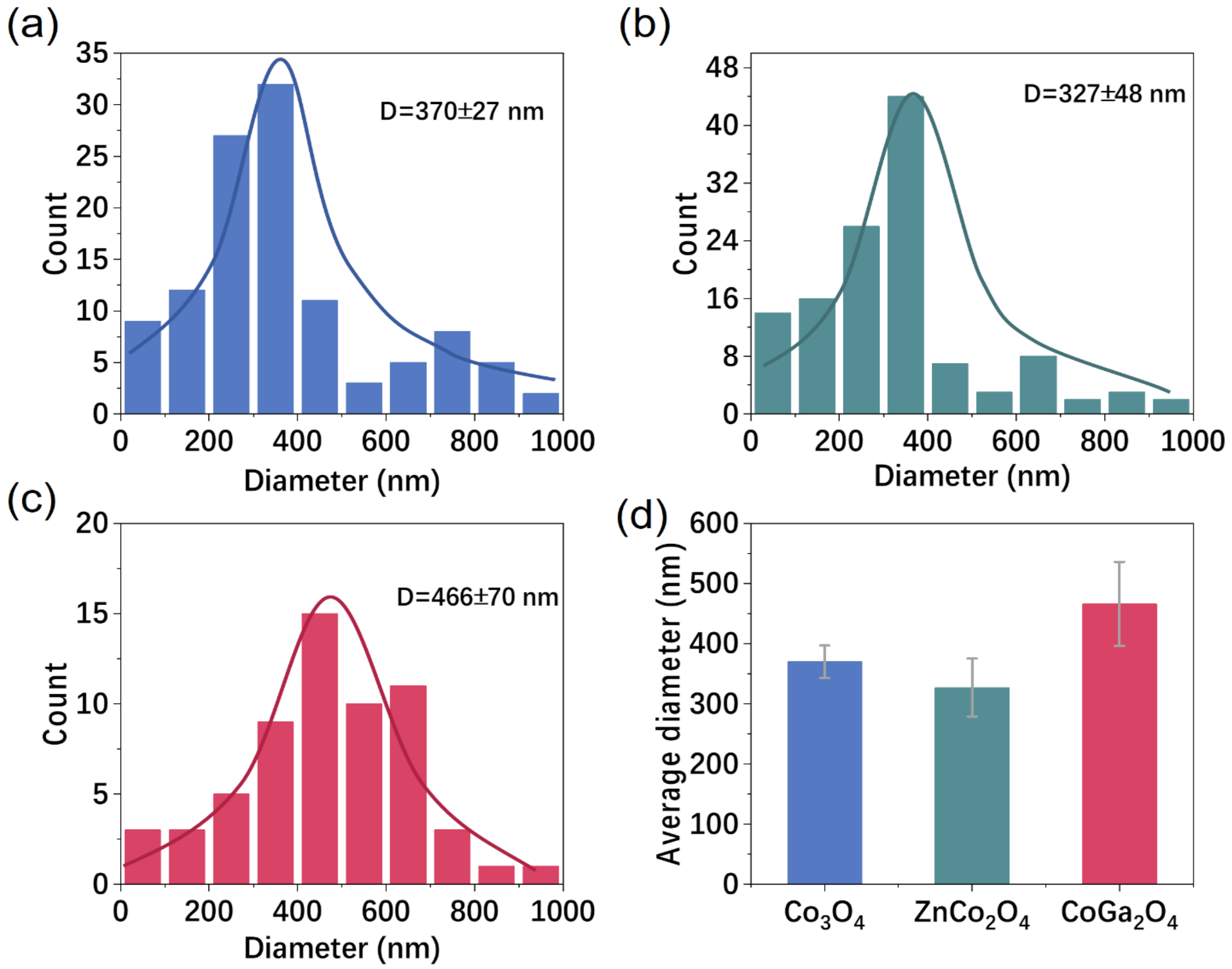

Supplementary Fig. 8 | Particle size distributions of **(a)** Co_3O_4, **(b)** $ZnCo_2O_4$ and **(c)** $CoGa_2O_4$ octahedra prepared by sol-gel method obtained with **Supplementary Fig. 6**; **(d)** Average particle sizes of the as-prepared three samples. Error bars are standard error values of three tests (n = 3). Source data are provided as a Source Data file.

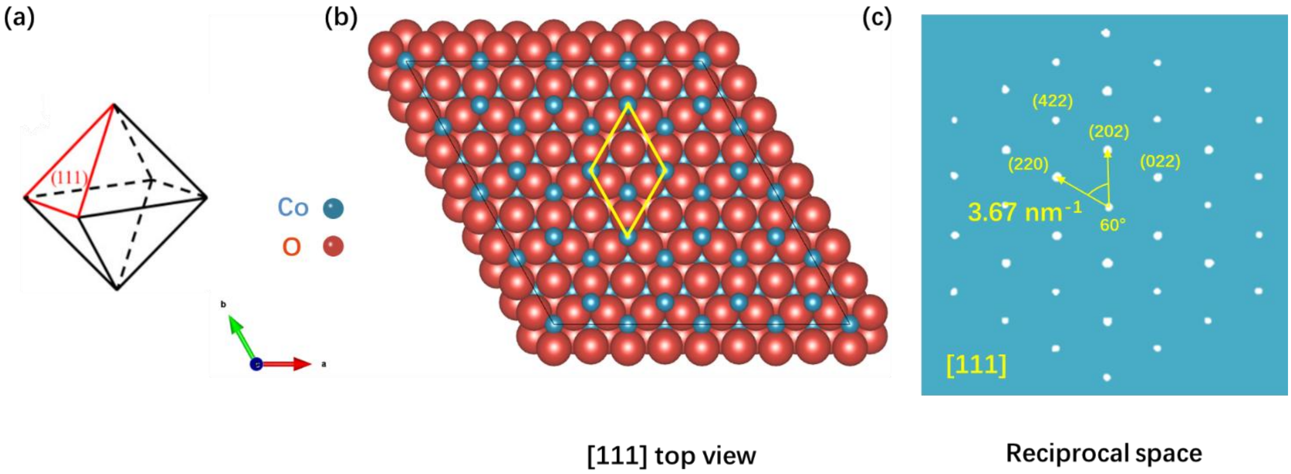

Supplementary Fig. 9 | Illustration of method used for determining the exposed facet. (a) Schematic illustration of octahedral spinel Co_3O_4 with (111) facets. Atomic structure of [111] surface of the top view **(b)**. Standard diffraction pattern of spinel Co_3O_4 in [111] direction in the reciprocal space **(c)**.

Crystal spacings (norms of the vectors) and angles of the crystal planes measured in the reciprocal space (such as FFT patterns and SAED plots) are of great significance to indexing the diffraction vectors and determining the exposed crystal facet[17]. Typically, three crystal planes parallel to the same line (the normal line perpendicular to the diffraction plane) are required to be indexed. And these three crystal planes need to satisfy the parallelogram rule: $V_{h_1k_1l_1}+V_{h_2k_2l_2}=V_{h_3k_3l_3}$. As shown in **Supplementary Fig. 9**, the crystal planes determining the [111] facet are $V_{(220)}$, $V_{(202)}$, and $V_{(422)}$, respectively, with the interplanar angle of 60 ° between the (220) and (202) crystal plane. The interplanar crystal spacing between these crystal planes is 3.67 nm^{-1}.

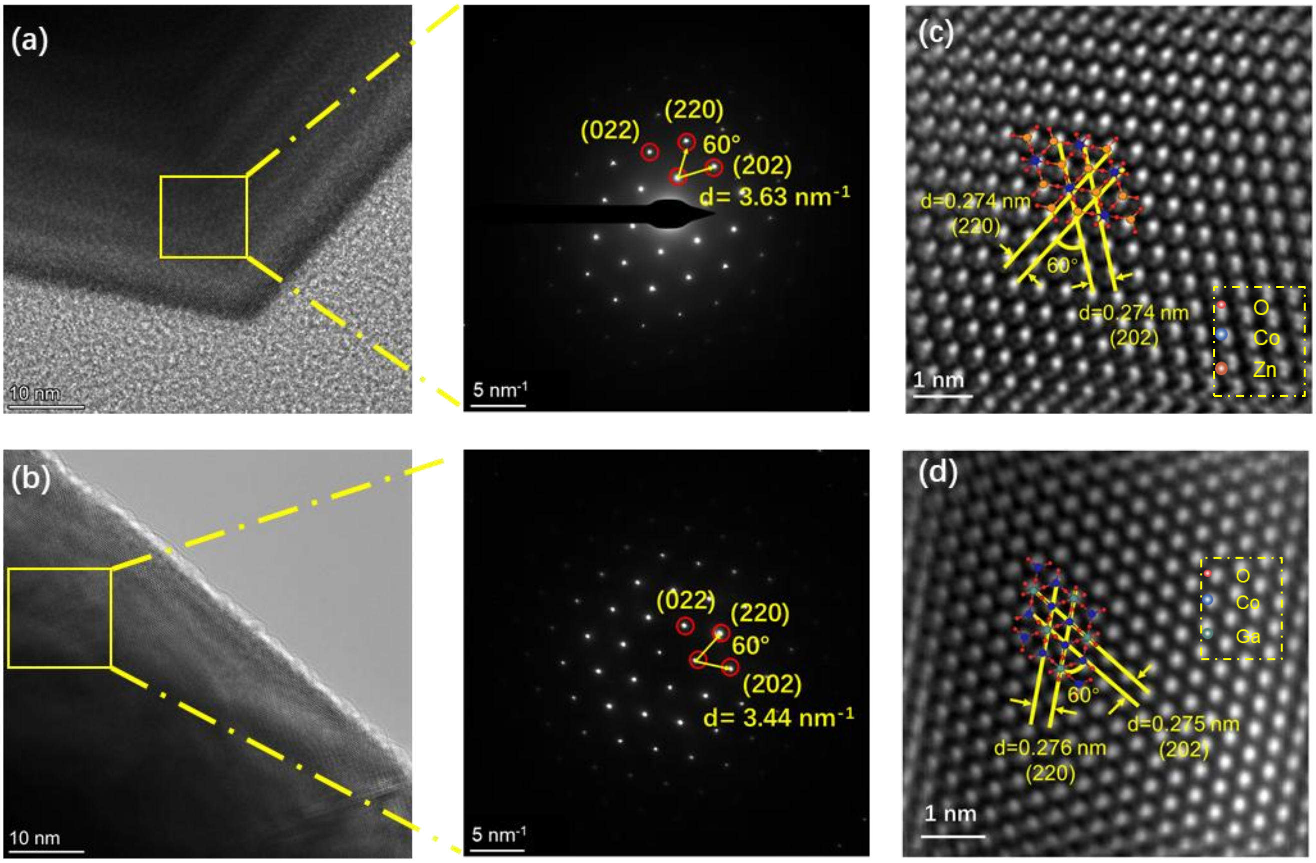

Supplementary Fig. 10 | HRTEM images of $ZnCo_2O_4$ **(a)** and $CoGa_2O_4$ **(b)** in [111] orientation and their corresponding SAED images and atomic resolved HRTEM images (**c-d**).

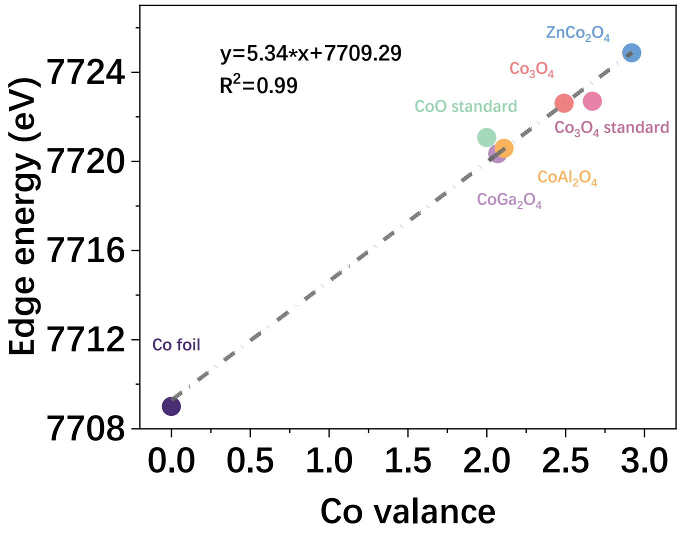

Supplementary Fig. 11 | Average oxidation states of Co adapted from ref.[18].

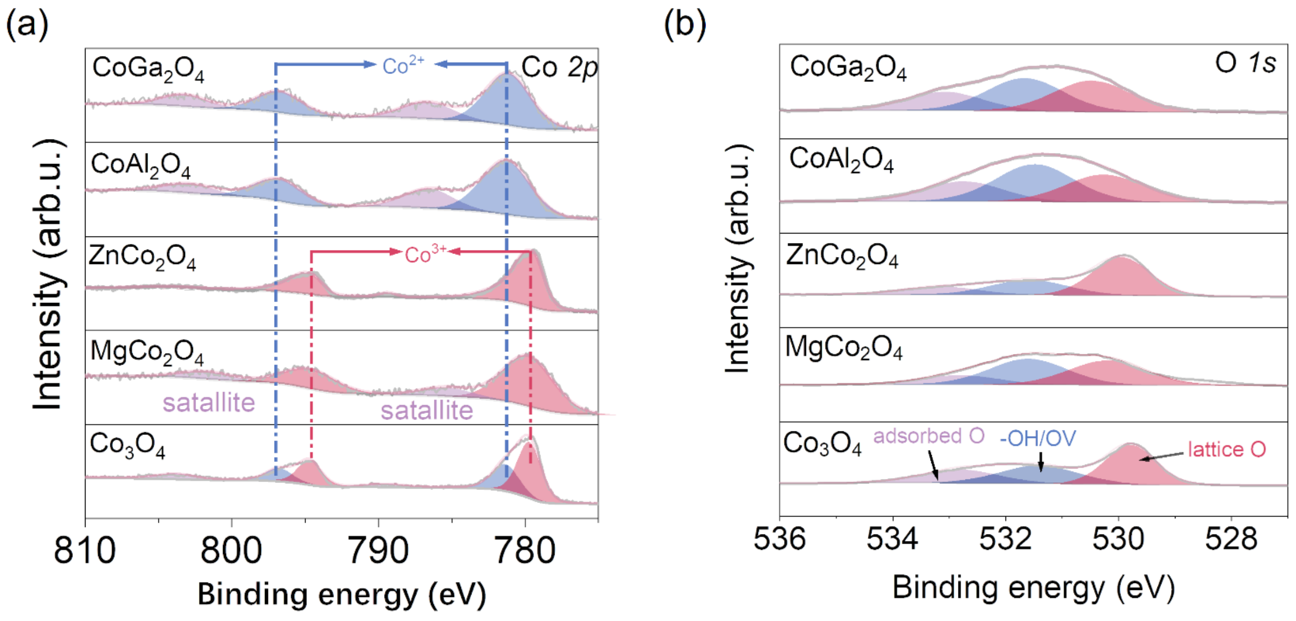

Supplementary Fig. 12 | High-resolution XPS surveys on Co *2p* **(a)** and O *1s* **(b)** for spinel oxides.

All the Co *2p* XPS spectra display a pair of peaks attributable to Co $2p_{3/2}$ and Co $2p_{1/2}$, which are generated by Co *2p* spin-orbital doublet. The subpeaks 781.3 and 796.81eV are assigned to Co^{2+}, whereas the peaks at 780.1 and 794.8 eV are attributed to Co^{3+}. Thus, it can be confirmed that the valence states of exposed Co ions in $CoGa_2O_4$/$CoAl_2O_4$ and $ZnCo_2O_4$/$MgCo_2O_4$ are +2 and+3, respectively, while Co^{2+} and Co^{3+} coexist in Co_3O_4.

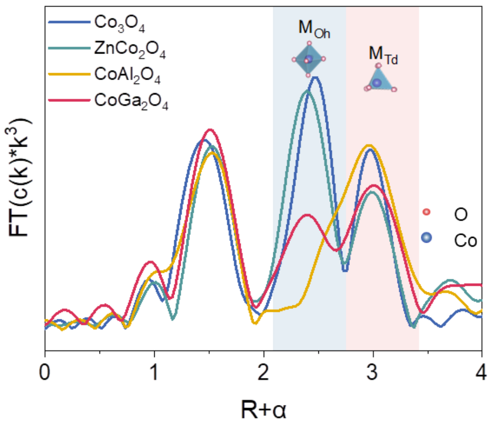

Supplementary Fig. 13 | The corresponding Fourier transformed EXAFS (FT-EXAFS) spectra at R space.

FT-EXAFS spectra indicated that the peak intensity ratio of Co_{Oh}-Co_{Oh} path (at 2.5 Å) to Co_{Td}-Co_{Oh} path (at 3.0 Å) for Co_3O_4 and $CoGa_2O_4$ were 1.39 and 0.79, respectively. The significant decrease of the peak intensity ratio suggests that most of the Co octahedral sites have been replaced by Ga^{3+} cations, while the considerably decreased Co_{Oh}-Co_{Oh} path for $CoGa_2O_4$ might originate from minor presence of $Co^{2+}{}_{Oh}$ sites and the interference signal of Ga *d* orbital electrons. To investigate the influence of this interference signal from Ga, we recorded the FT-EXAFS spectrum of $CoAl_2O_4$, which was synthesized using the same protocol as these Co-based spinels, and compared it to that of $CoGa_2O_4$. Evidently, the absence of d orbital in Al^{3+} diminished the scattering path at ~2.5 Å. This suggests $Co^{2+}{}_{Oh}$ sites as the minor coordination-geometry-competitor against the dominant $Co^{2+}{}_{Td}$ sites.

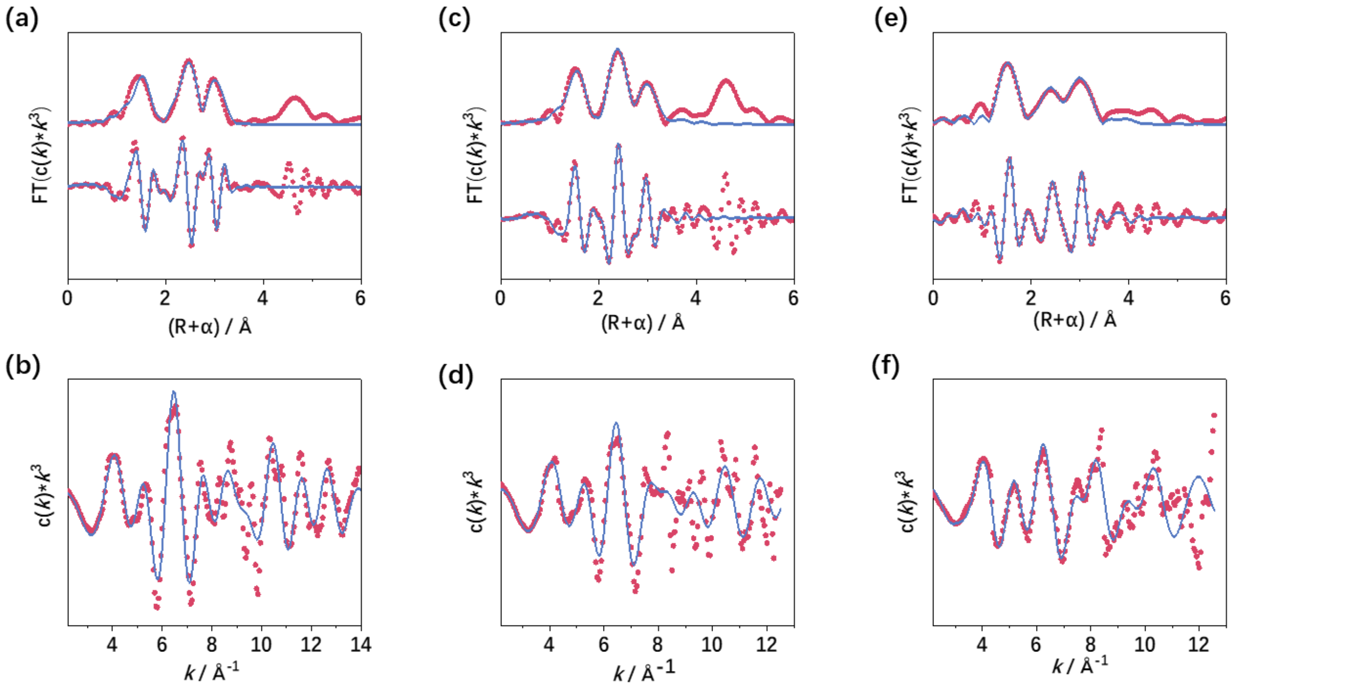

Supplementary Fig. 14 | The EXAFS fitted curves of Co_3O_4 (**a**) at R space and (**b**) at k space. The EXAFS fitted curves of $ZnCo_2O_4$ (**c**) at R space and (**d**) at k space. The EXAFS fitted curves of $CoGa_2O_4$ (**e**) at R space and (**f**) at k space.

Noted that the radial distances in EXAFS spectra were not phase-corrected, and the abscissa was thus represented as (R+α) to convert the apparent distances to the real bond distances by adding a typical value of 0.3−0.4 Å[12, 19]. For an easy discussion, the radial distances are referred to the apparent distances.

The following parameters can be derived by fitting curves to the k^3-weighted EXAFS spectra of spinel oxides: coordination number (N), absorber-backscatter distance (R), and Debye-Waller parameter (σ). The coordination number was restricted to the expected values in the octahedral and tetrahedral sites of the spinel structure, with the scattering path fixed to show variations in atomic distances between different spinel oxides. EXAFS fitting range (r-range) was set as 1-3.5 Å[20].

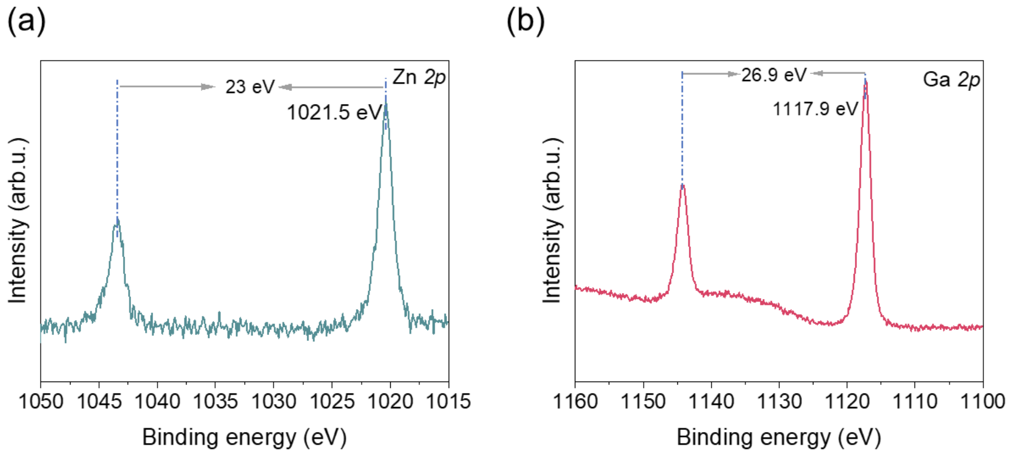

Supplementary Fig. 15 | High resolution XPS survey on Zn *2p* of $ZnCo_2O_4$ (**a**) and Ga *2p* of $CoGa_2O_4$ (**b**).

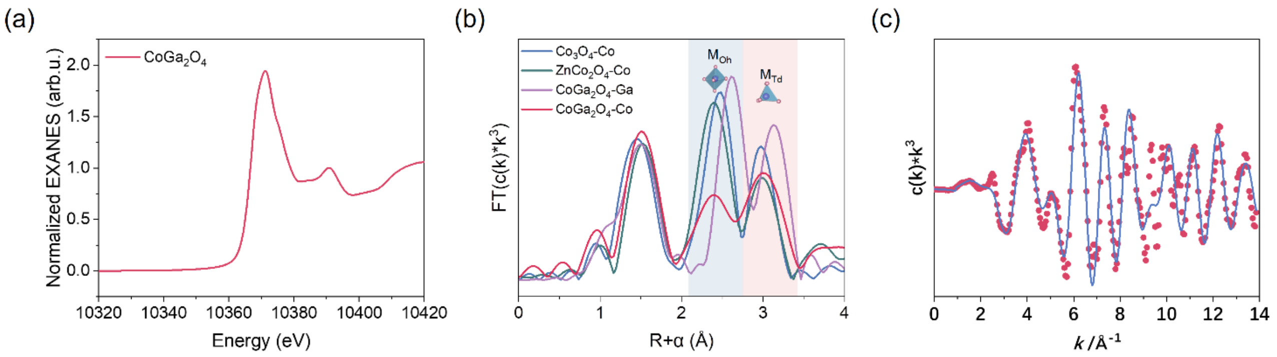

Supplementary Fig. 16 | Ga K-edge XANES spectrum **(a)** and the corresponding EXAFS results at R space **(b)** and at k space **(c)**.

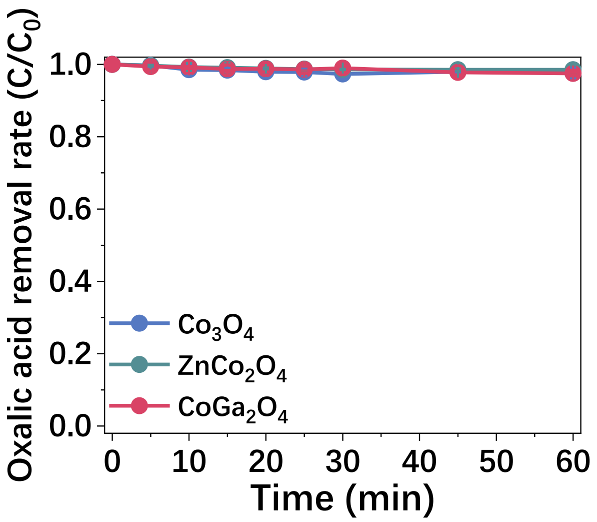

Supplementary Fig. 17 | Oxalic acid removal profiles by adsorption with Co_3O_4, $ZnCo_2O_4$, and $CoGa_2O_4$. Catalyst loading: 0.1 g L^{-1}; temperature: 25 °C; ozone flow rate: 100 mL min^{-1}; ozone concentration: 25 mg L^{-1}; initial pH was adjusted to 3 by adding 0.01 M H_2SO_4/NaOH in OA solution. $[OA]_0$: 50 mg L^{-1}. No ozone was injected in the adsorption tests. Error bars are standard error values of three tests (n = 3). Source data are provided as a Source Data file.

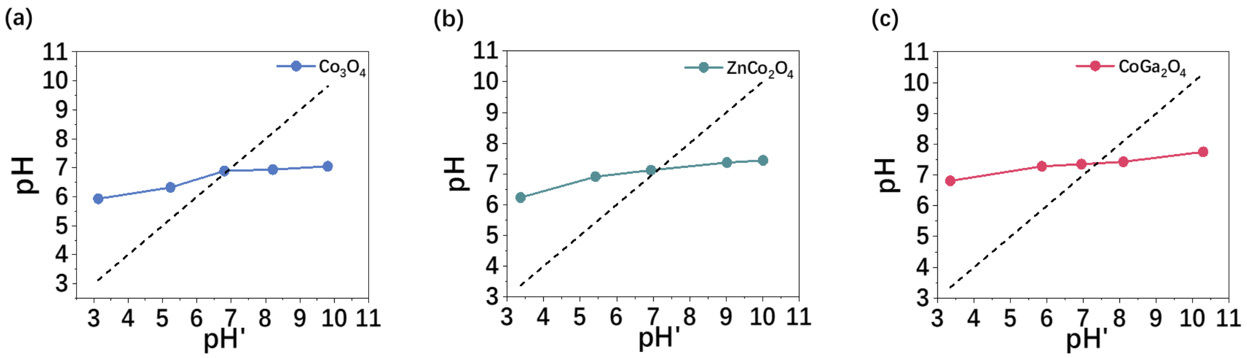

Supplementary Fig. 18 | pH-drift method for determining the pH_{pzc}s of Co_3O_4 **(a)**, $ZnCo_2O_4$ **(b)**, and $CoGa_2O_4$**(c)**.

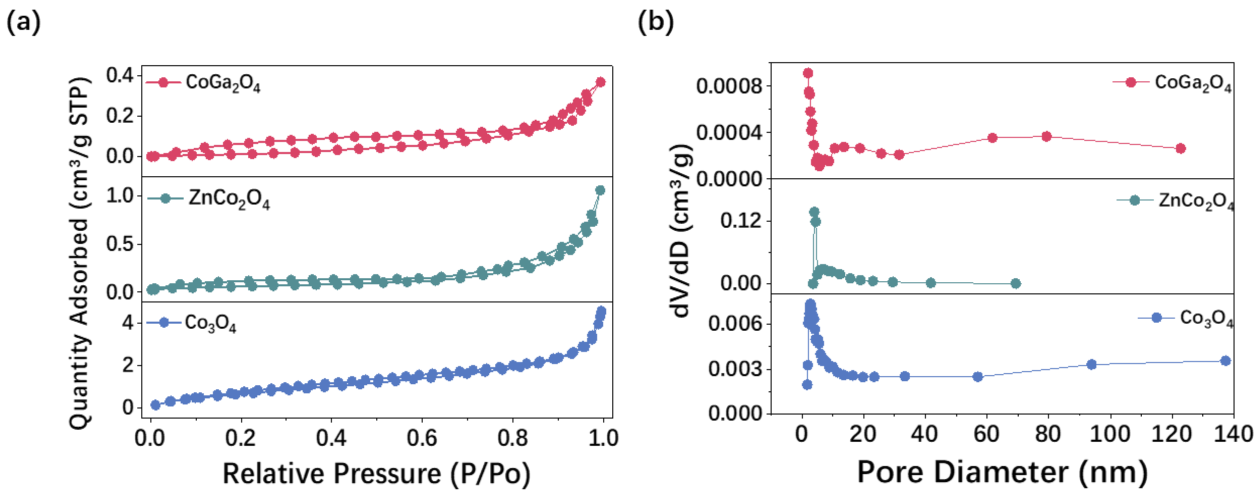

Supplementary Fig. 19 | N_2 sorption isotherms **(a)** and pore size distributions **(b)** of the as-synthesized spinel oxides.

\

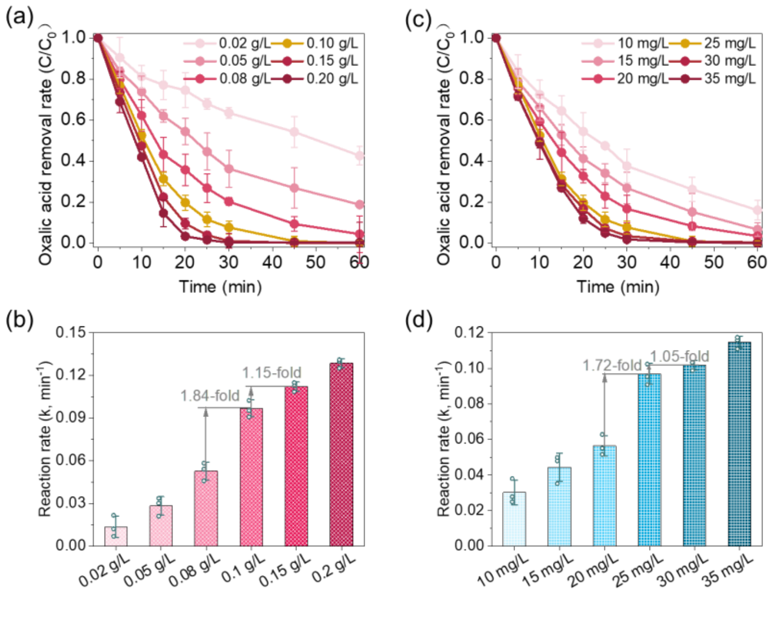

Supplementary Fig. 20 | Effects of reaction parameters on catalytic ozonation efficiency. Effect of catalytic loading **(a)** and O_3 dosage **(c)** on degradation of oxalic acid for $CoGa_2O_4$ and their corresponding rate constants comparison (**b** and **d**) Catalyst loading: 0.1 g L^{-1}; temperature: 25 °C; ozone flow rate: 100 mL min^{-1}; ozone concentration: 25 mg L^{-1}; initial pH was adjusted by adding 0.01 M H_2SO_4/NaOH in OA solution. $[OA]_0$: 50 mg L^{-1}. Error bars are standard error values of three tests (n = 3). Source data are provided as a Source Data file.

We investigated the activity of $CoGa_2O_4$ catalysts under different catalyst loadings and ozone dosages. The results indicated that increasing the catalytic loading and the ozone dosage beyond 0.1 g/L and 25 mg/L accordingly resulted in minor improvements of the catalytic activity. Excessive catalyst loading and the ozone dosage beyond those points led to a markedly diminished marginal benefit for improving the catalytic efficiency. Although a higher catalyst loading offers more active sites, the concentration of dissolved ozone in solution limits the reaction rate because the active sites on the catalysts have a high affinity for dissolved ozone but are relatively inert to gaseous O_3. Therefore, a catalyst loading of 0.1 g/L and an ozone dosage of 25 mg/L were identified as the optimum operating parameters in this study.

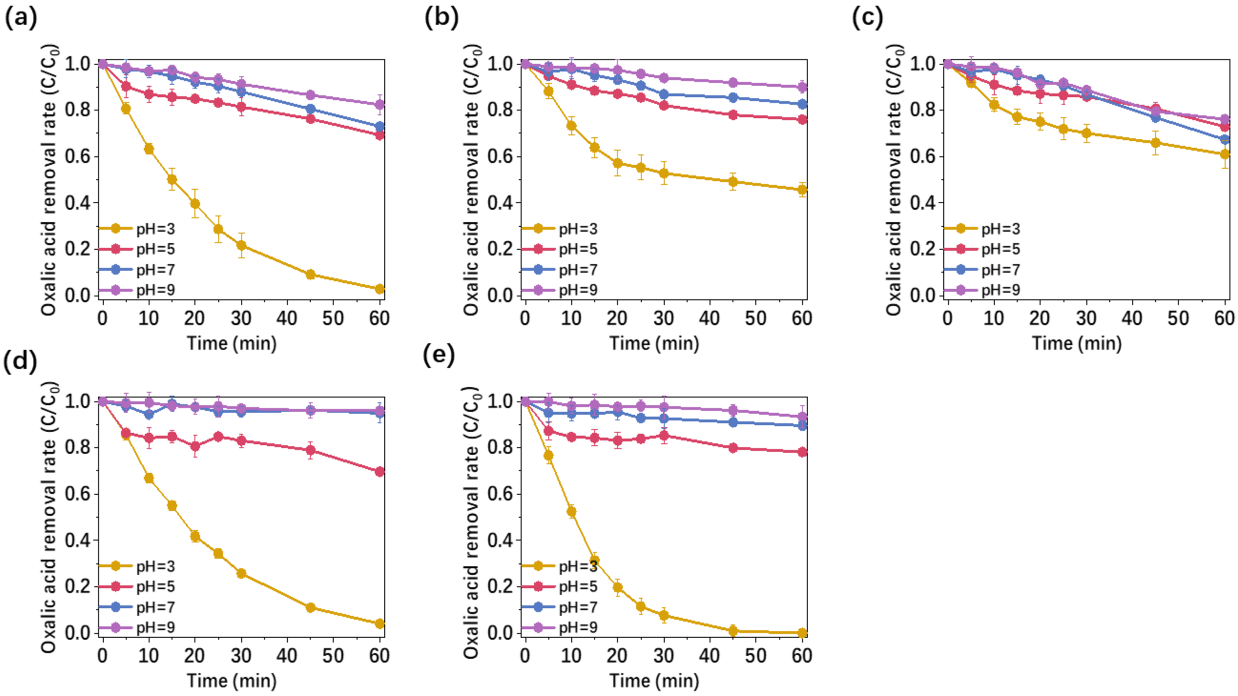

Supplementary Fig. 21 | Effect of initial solution pH on degradation of oxalic acid for Co_3O_4**(a)**, $MgCo_2O_4$ **(b)**, $ZnCo_2O_4$ **(c)**, $CoAl_2O_4$ **(d)**, $CoGa_2O_4$ **(e)**. Catalyst loading: 0.1 g L^{-1}; temperature: 25 °C; ozone flow rate: 100 mL min^{-1}; ozone concentration: 25 mg L^{-1}; initial pH was adjusted by adding 0.01 M H_2SO_4/NaOH in OA solution. $[OA]_0$: 50 mg L^{-1}. Error bars are standard error values of three tests (n = 3). Source data are provided as a Source Data file.

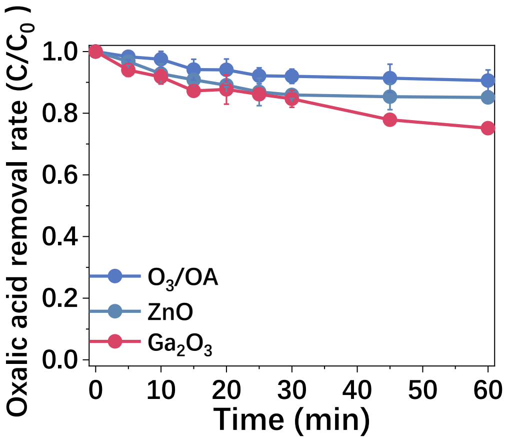

Supplementary Fig. 22 | Oxalic acid degradation profiles by ozonation and catalytic ozonation with ZnO and Ga_2O_3. Catalyst loading: 0.1 g L^{-1}; temperature: 25 °C; ozone flow rate: 100 mL min^{-1}; ozone concentration: 25 mg L^{-1}; initial pH was adjusted to 3 by adding 0.01 M H_2SO_4/NaOH in OA solution. $[OA]_0$: 50 mg L^{-1}. No ozone was injected in the adsorption tests. Error bars are standard error values of three tests (n = 3). Source data are provided as a Source Data file.

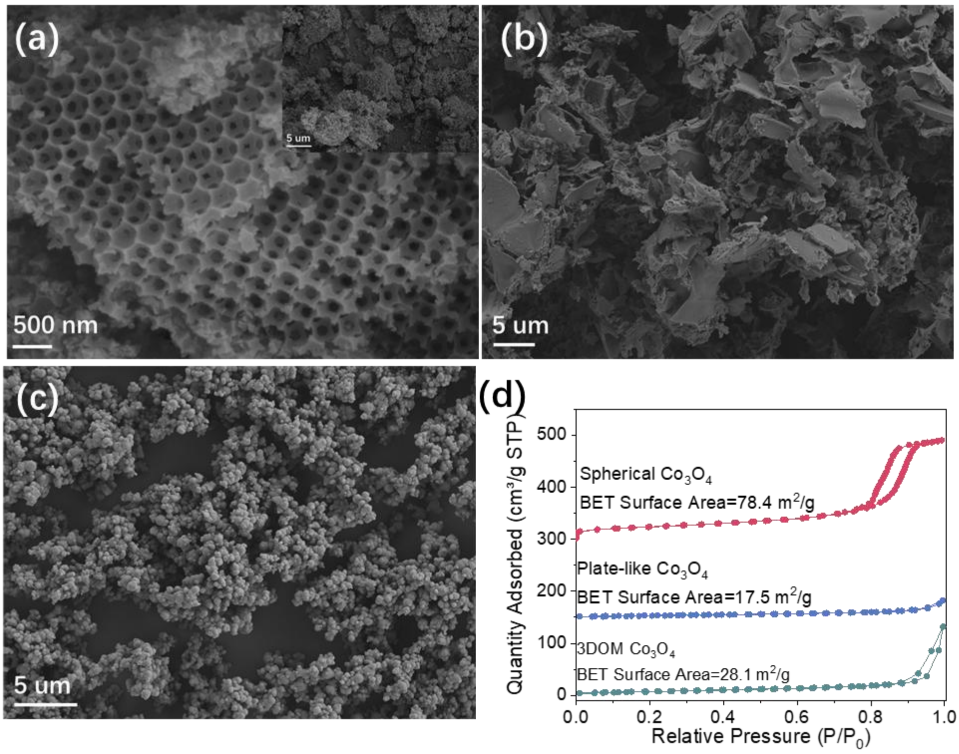

Supplementary Fig. 23 | Morphology and N_2 sorption isotherms for different Co_3O_4 samples. SEM images of 3DOM Co_3O_4 **(a)**, plate-like Co_3O_4 **(b)**, and spherical Co_3O_4 **(c)**. **(d)** N_2 sorption isotherms of synthesized Co_3O_4.

Synthesis of 3DOM-Co_3O_4

Three-dimensionally ordered poly methyl methacrylate (PMMA) microspheres with an average diameter of 350 nm were firstly synthesized according to a previously reported method[21, 22]. First, 120 mL of deionized water was added to a 500 mL four-neck flask, followed by the introduction of argon gas (0.5 L/h) to purge the air inside. The flask was then heated in an oil bath for 30 min to remove any remaining air. Next, 12 mL of methyl methacrylate was added to 120 mL deionized water, and the solution was maintained at 70 °C with stirring at 350 rpm to stabilize the temperature. Subsequently, potassium persulfate (30 mL, 0.12 g preheating to 70 °C) was added, and after 2 h reaction, a milky white liquid was achieved and transferred to a centrifuge. The centrifugation was conducted at 4000 rpm for 10 h to achieve

ordered packing of the microspheres. After centrifugation, the supernatant was decanted, and the solid was dried in an oven at 40 °C for 24 h. The yielded well-ordered PMMA microspheres with a diameter of approximately 350 nm. For synthesis of 3DOM-Co_3O_4, 30 mmol of $Co(NO_3)_2 \cdot 6H_2O$ and 30 mmol citric acid were completely dissolved in a solution containing 10 mL deionized water and 10 mL methanol under stirring, and then 1.5 g of the as-synthesized ordered PMMA microspheres were soaked into the above solution and kept static for 24 h. After filtration, the solid powders were dried at 50 °C for 24 h, then calcined at 500 °C for 4 h under the air atmosphere. And 3DOM-Co_3O_4 with an average macroporous size of 290 nm was obtained. The yield of 3DOM-Co_3O_4 is around 1.2%.

Synthesis of plate Co_3O_4

In a typical synthesis, 30 mmol of $Co(NO_3)_2 \cdot 6H_2O$ and 30 mmol citric acid were dissolved in a solution containing 10 mL DI water and 10 mL methanol under stirring, and then the solution was dried at 50 °C for 120 h. After the drying process, the mixed precursors were grinded into fine powders, followed by calcination at 500°C for 4 h under the air atmosphere in a muffle furnace with a heating rate of 5 ºC/min. The yield of plate Co_3O_4 is around 8%.

Synthesis of spherical Co_3O_4

To prepare the spherical Co_3O_4, 0.4 mol of $Co(NO_3)_2 \cdot 6H_2O$ and 0.03 mol of NaOH and were dissolved in 40 mL of DI water with vigorous stirring. After 1 h, the solution was moved into a Teflon-lined autoclave and heated at 180 °C for 5 h. The obtained solution was filtered with DI water and ethyl alcohol and dried overnight at 50 °C. Subsequently, the dried powders were calcined at 500 °C for 3 h in the air atmosphere with a heating rate of 5 ºC/min. The yield of spherical Co_3O_4 is around 1.1%.

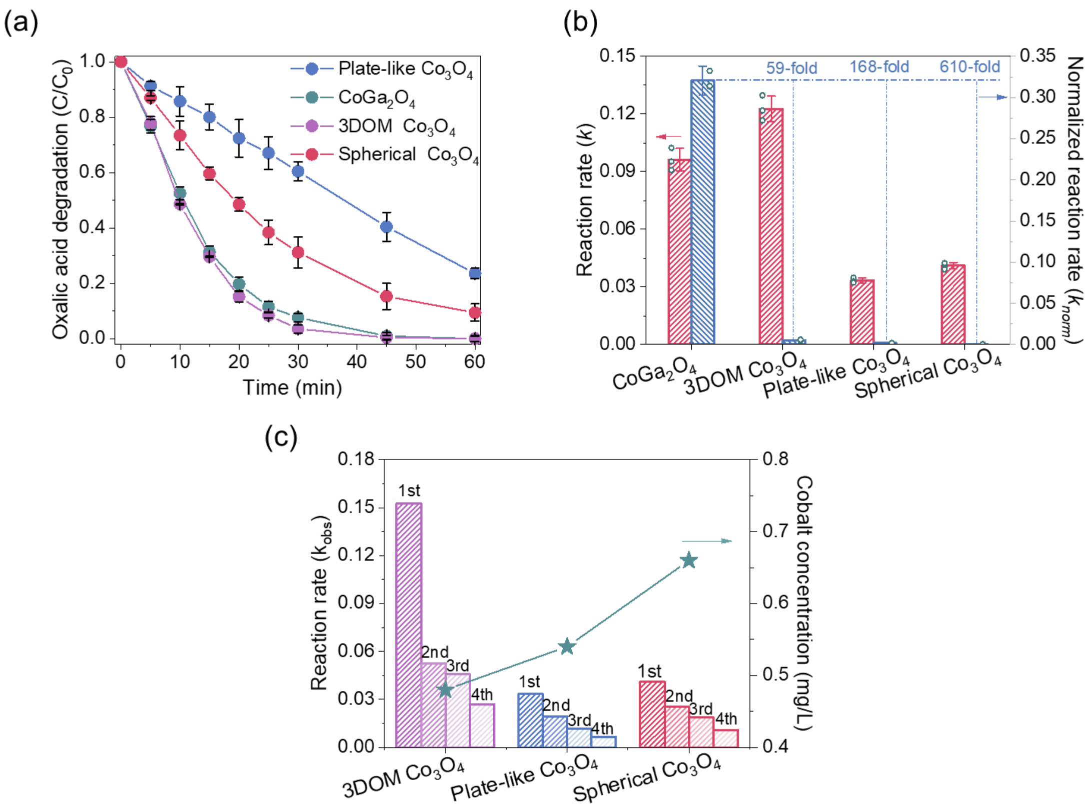

Supplementary Fig. 24 | Catalytic ozonation performance and recyclability tests of different Co_3O_4 samples. (a) Catalytic ozonation for OA degradation with synthesized Co_3O_4 samples; **(b)** Comparison of reaction rates and normalized reaction rates of different Co_3O_4/O_3 systems. **(c)** Cyclic experiments for the as-synthesized Co_3O_4 with high SSAs and the corresponding cobalt leaching concentrations. [catalyst]=0.1 g L^{-1}; temperature: 25 °C; initial pH=3, $[OA]_0$: 50 mg L^{-1}. Error bars are standard error values of three tests (n = 3). Source data are provided as a Source Data file.

The recyclability of these synthesized Co_3O_4 with high SSAs was also evaluated by a multi-cycle reusability test. Clearly, the Co_3O_4 with high SSAs demonstrated much inferior recyclability than the $CoGa_2O_4$. Strong passivation was observed after the 3rd cycle. Moreover, elevated cobalt leaching was observed for both plate-like Co_3O_4 and spherical Co_3O_4, as indicated by the inductively coupled plasma mass spectrometry (ICP-MS) results (0.54 and 0.66 mg/L), which exceed the national wastewater

discharge standard of China (0.5 mg/L) and contribute to secondary pollution. In contrast, the strong bonding in $CoGa_2O_4$ crystal structure and the fast reaction kinetics at the catalyst surface guarantee the structural integrity of $CoGa_2O_4$ during the multi-cycle reusability test. As a result, only minor passivation was observed after 4 cycles with a negligible amount of leached Co^{2+} (0.09 mg/L).

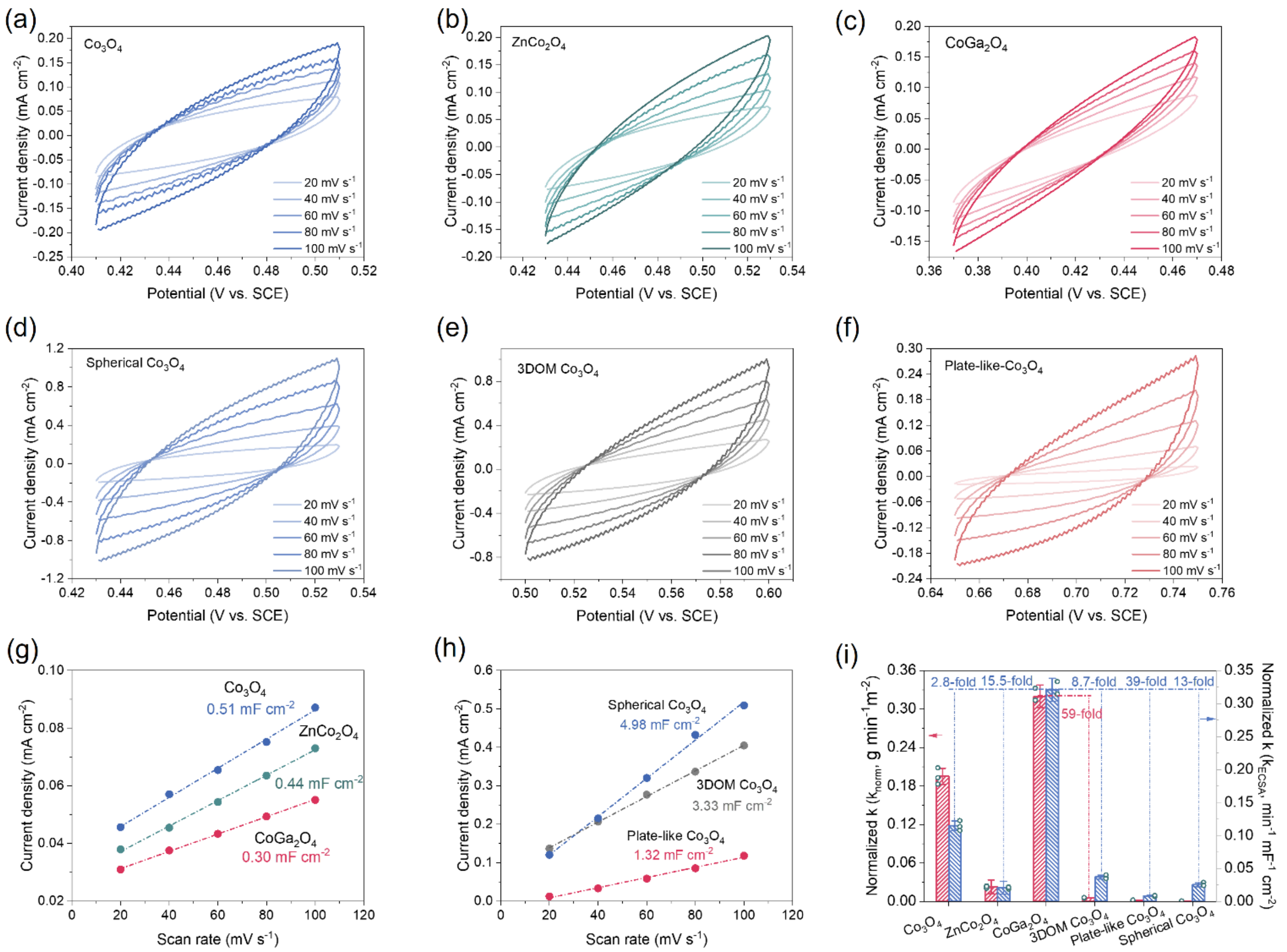

Supplementary Fig. 25 | Electrochemical active surface area tests and comparison of the normalized catalytic ozonation performance of different Co_3O_4 samples. Cyclic Voltammetry (CV) curves of Co_3O_4 **(a)**, $ZnCo_2O_4$ **(b)**, $CoGa_2O_4$ **(c)**, spherical Co_3O_4 **(d)**, 3DOM Co_3O_4 **(e)**, and plate-like Co_3O_4 **(f)** at different scan rates, and ECSA measurements of samples **(g)** and **(h)**. **(i)** Comparison of the BET surface area normalized reaction rates (k_{norm}s) and ECSA normalized reaction rates (k_{ECSA}s) of different spinels/O_3 systems. The electrolyte contained 100 mg L^{-1} Na_2SO_4 (pH=3) and the 0.1 mg cm^{-2} catalyst was loaded on glassy carbon-rotating disk electrode (GC-RDE). Error bars are standard error values of three tests (n = 3). Source data are provided as a Source Data file.

Considering the electron transfer nature of the heterogeneous O_3 activation process, we further measured the electrochemical active surface areas (ECSAs) of the as-synthesized catalysts to examine the number

of the exposed active sites during the reaction. The intrinsic activity of the active sites with different coordination geometries was further compared by ECSAs normalized reaction kinetics ($k_{ECSA}s$), which served as a strong complementary of SSAs normalized reaction kinetics (k_{norm}s) to exclude the influence of exposed active sites.[23] By fitting the non-Faradaic currents from cyclic voltammetry measurements at different scan rates, the ECSAs of Co_3O_4, $CoGa_2O_4$, $ZnCo_2O_4$, and 3DOM Co_3O_4 are derived as 0.50, 0.30, 0.44, and 3.3 mF cm^{-2}, respectively. This suggests that 3DOM Co_3O_4 exposes the highest number of active sites during the heterogeneous reactions owing to its periodic structure, which agrees with its highest BET SSA. The $k_{ECSA}s$ can provide a more accurate representation of the intrinsic activity of the exposed active sites than reaction kinetics normalized by BET SSAs (k_{norm}s). It is found that the exposed Co sites in $CoGa_2O_4$ obtained an 8.7-fold higher intrinsic activity than those in 3DOM Co_3O_4. Additionally, the intrinsic activity of the Co sites in $CoGa_2O_4$, Co_3O_4, and $ZnCo_2O_4$ aligns well with the trends observed in the k_{norm}s comparison. These results collectively reinforce the notion that regulating coordination geometry in Co-based spinel outperforms SSA engineering in determining the intrinsic activity of the active sites.

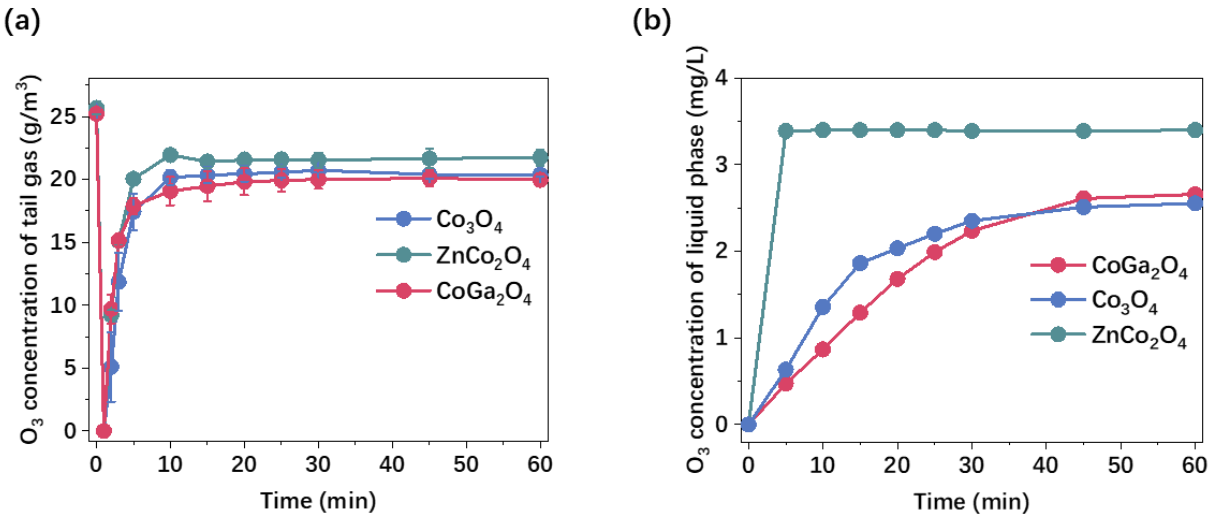

Supplementary Fig. 26 | Variations of O_3 concentrations in the off-gas **(a)** and in reaction solution **(b)**. Reaction conditions: Catalyst loading: 0.1 g L^{-1}; temperature: 25 °C; ozone flow rate: 100 mL min^{-1}; ozone concentration: 25 mg L^{-1}; initial pH was adjusted by adding 0.01 M H_2SO_4/NaOH in OA solution. $[OA]_0$: 50 mg L^{-1}. Error bars are standard error values of three tests (n = 3). Source data are provided as a Source Data file.

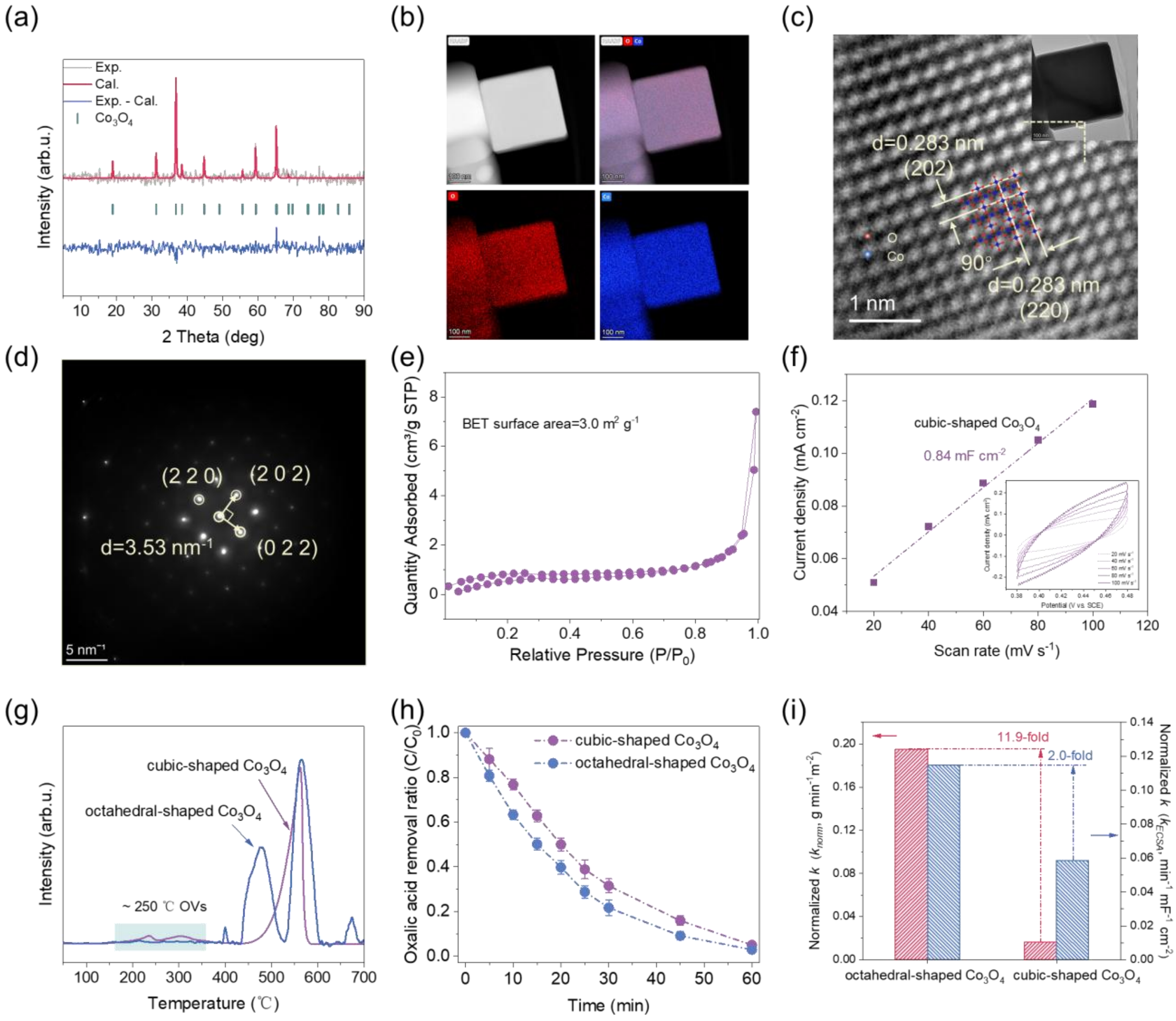

Supplementary Fig. 27 | Comparisons of physiochemical properties and catalytic performance of cubic-shaped and octahedral-shaped Co_3O_4. (a) Rietveld-refined XRD patterns of cubic-shaped Co_3O_4. **(b)** EDX mapping images of cubic-shaped-Co_3O_4. Atomic-resolved high-resolution TEM (HRTEM) image **(c)** and the corresponding SAED image (d) of cubic-shaped-Co_3O_4. **(e)** N_2 sorption isotherms of synthesized cubic-shaped-Co_3O_4. **(f)** ECSA measurements of cubic-shaped-Co_3O_4. **(g)** O_2-TPD profiles of cubic-shaped-Co_3O_4 and octahedral-shaped Co_3O_4. **(h)** Catalytic ozonation activity of cubic-shaped-Co_3O_4 and octahedral-shaped Co_3O_4. **(i)** Comparison of the k_{norm}s and k_{ECSA}s for cubic-shaped-Co_3O_4 and octahedral-shaped Co_3O_4. Error bars are standard error values of three tests (n = 3). Source data are

provided as a Source Data file.

We further synthesized cubic-shaped Co_3O_4 with the exposed crystal facet of [100] as the comparative material and evaluated its catalytic ozonation activity [24]. The synthesis method is described as below.

Synthesis of cubic-shaped Co_3O_4

In a typical synthesis, 0.04 mol of cobalt nitrate hexahydrate and 0.01 mol of sodium hydroxide were dissolved in 40 mL of deionized water under vigorous stirring. Once the mixture was thoroughly dissolved, it was transferred to a Teflon-lined autoclave and subjected to hydrothermal treatment at 180 °C for 5 h. After the heating process, the solid product was collected by filtering and washing with deionized water and ethyl alcohol. The obtained product was then dried at 80 °C overnight.

The XRD with Rietveld refinement results (**Supplementary Fig. 27a**) and the EDX mappings (**Supplementary Fig. 27b**) suggest the successful synthesis of Co_3O_4 spinel oxide. We further determined the exposed crystal facet of cubic-shaped Co_3O_4 (**Supplementary Fig. 27c**) using the same protocol as the other Co-based spinels via measuring interplanar crystal spacing and angle of the crystal planes measured in the SAED plot [24] (**Supplementary Fig. 27d**). The interplanar crystal spacing of 0.283 nm and a fixed interplanar angle of 90 ° between the (0 2 2), (2 0 2), and (0 2 2) crystal plane suggest the [100] crystal plane as the dominated exposed facet. By normalizing their reaction kinetics with BET SSAs/ECSA (**Supplementary Fig. 27h&i**), we found that cubic-shaped Co_3O_4 exhibited a 2.0-fold lower intrinsic activity than octahedral-shaped Co_3O_4 with the exposed crystal facet of [111], revealing that the [111] facet indeed accelerated the reaction kinetics.

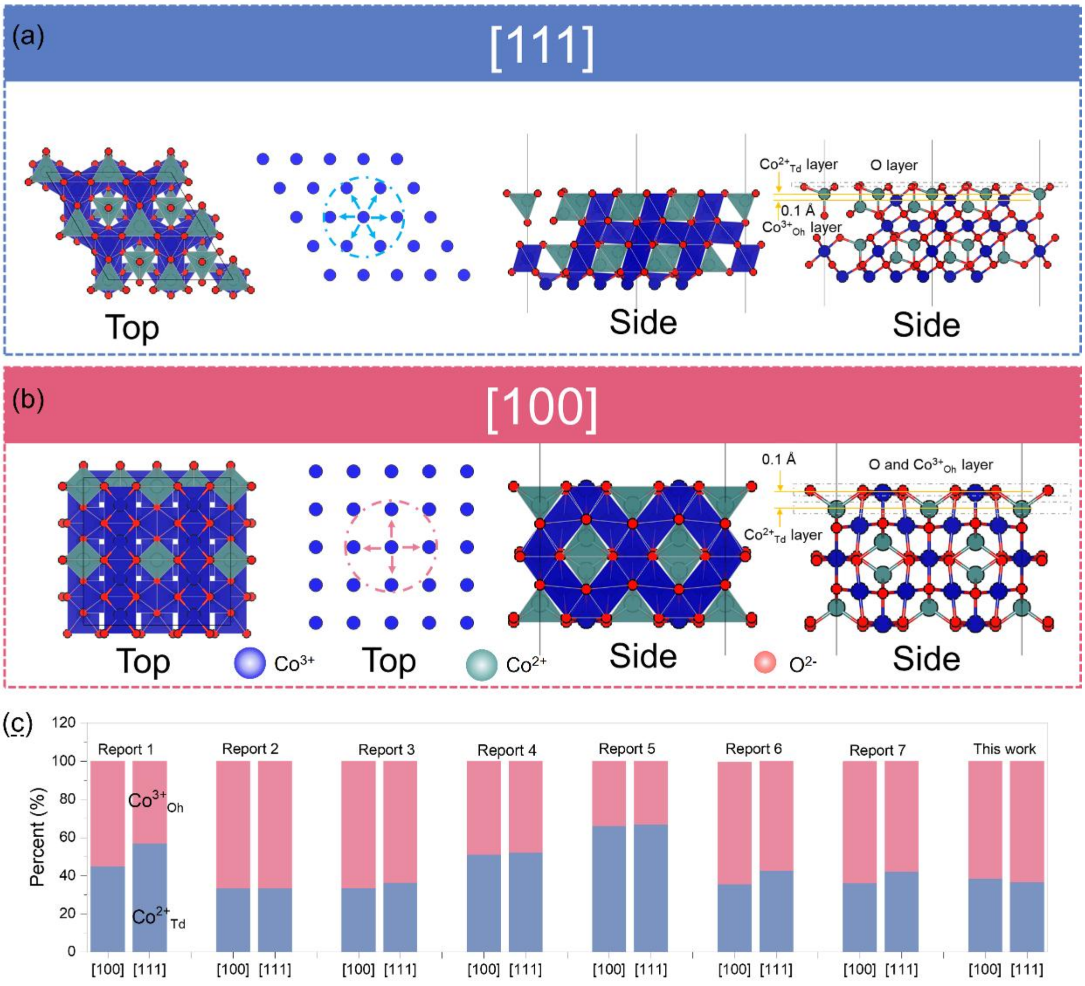

Supplementary Fig. 28 | Distribution of Co sites on different exposed crystal planes. Theoretical models of [111] **(a)** and [100] **(b)** crystal facets of Co_3O_4 and the spatial distribution of Co atoms on each facet. **(c)** Reported distribution of cobalt valence states across different crystal facets of Co_3O_4. Report include Report 1[2], Report 2[3], Report 3[4], Report 4[5], Report 5[6], Report 6[7], and Report 7[8].

In the [111] crystal facet, the angle between the resided Co atoms is 60 °, which is smaller than the 90 ° angle of the resided Co atoms in the [100] crystal facet. This enables [111] facet to reside a higher number of Co atoms within the same reaction area than [100] facet.

We then differentiated the proportions of geometric sites on these crystal facets. By constructing theoretical models of the [111] and [100] crystal facets, we find that although Co^{3+}_{Oh} and Co^{2+}_{Td} are the

exposed geometric sites on [100] and [111] facets, respectively, the interlayer spacing of Co^{2+}_{Td} and Co^{3+}_{Oh} sites in both facets is only ~0.1 Å, which is significantly lower than the inter-atom spacing of the Co sites (>2 Å). This tiny interlayer spacing suggests that the dependence of exposed geometric sites on crystal facet is only theoretically applicable. In actual characterizations and reactions, both Co^{2+}_{Td} and Co^{3+}_{Oh} sites are present on the different crystal facets and the compositional proportions of Co^{2+}_{Td} and Co^{3+}_{Oh} sites among the exposed atoms on [100] and [111] facets are similar (**Supplementary Fig. 28c** and **Supplementary Table 11**). This study follows this trend as well. Given the similar proportion of Co^{2+}_{Td} and Co^{3+}_{Oh} sites among the exposed atoms on [100] and [111] facets, the [111] facet with a higher atomic density obtains a greater number of the active Co^{2+}_{Td} sites, which accounted for its high activity in O_3 activation.

It is noteworthy that the sparse distribution of Co atoms in [100] facet can promote local structural reconfiguration and facilitate the formation of surface structural defects such as oxygen vacancies and strain defects. As revealed in O_2-TPD profiles in **Supplementary Fig. 27g**, a greater amount of surface oxygen vacancies are observed on cubic-shaped Co_3O_4 with the [100] facet than that of octahedral-shaped Co_3O_4 with the [111] facet. These structural defects can also act as the active sites for O_3 activation. In our study, the higher activity of the [111] facet than the [100] facet suggests that the contribution of exposed number of active sites to activity outperforms that of surface defective structures. This further highlights the governing role of geometric coordination sites in catalytic activity.

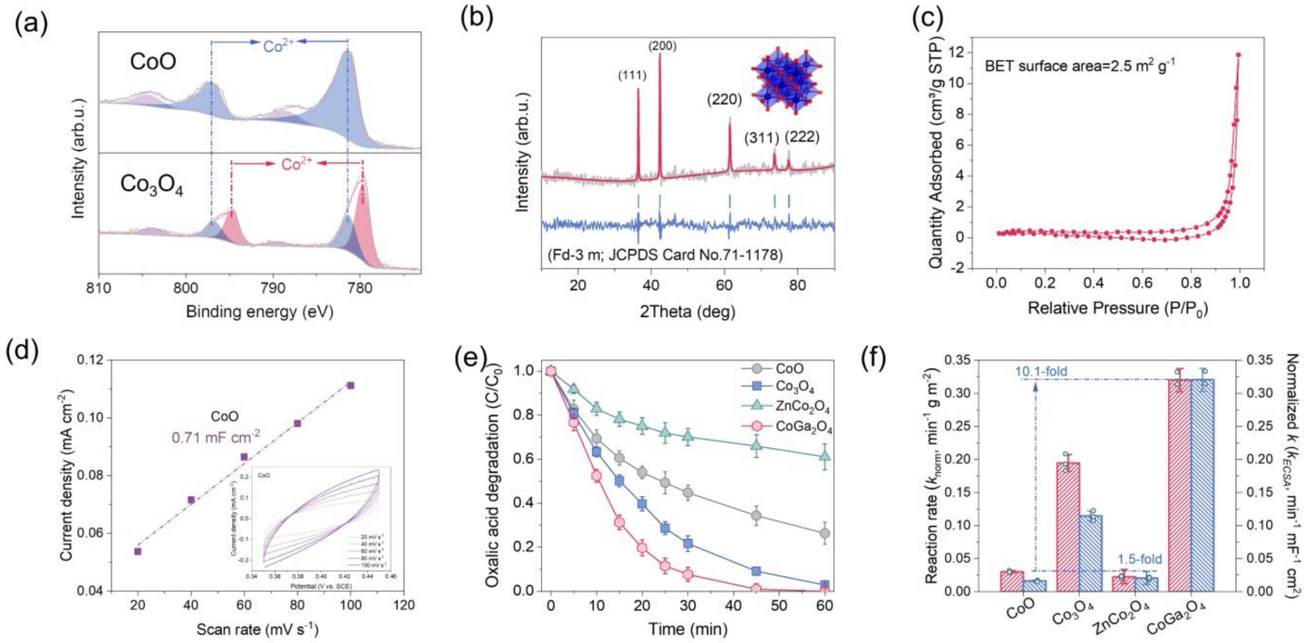

Supplementary Fig. 29 | Characterization and catalytic performance evaluation of CoO. (a) High resolution XPS surveys on Co *2p* for CoO and Co_3O_4. **(b)** Rietveld-refined XRD patterns of CoO. **(c)** N_2 sorption isotherms of synthesized CoO. **(d)** ECSA measurements of CoO with inset of the corresponding CV curves with different scanning rates. **(e)** Catalytic ozonation activity of CoO and spinel oxides. **(f)** Comparison of k_{ECSA}s and k_{norm}s of CoO and spinel oxides. Error bars are standard error values of three tests (n = 3). Source data are provided as a Source Data file.

Synthesis of CoO

The Co_3O_4 powder was placed into a corundum crucible and then transferred into a tube furnace. Subsequently, the sample was calcined at 900 °C for 4 h under an Ar atmosphere.

To differentiate the intrinsic catalytic activity between Co^{2+}_{Td} sites with Co^{2+}_{Oh} sites, we synthesized CoO with Co^{2+}_{Oh} as the predominated Co sites according to the previous reported method[25]. XPS survey confirms the absence of Co^{3+} (**Supplementary Fig. 29a**), while XRD Rietveld refinement result suggests the successful preparation of the CoO with Co^{2+}_{Oh} as the single crystal phase (**Supplementary Fig. 29b**). We evaluated the catalytic ozonation activity of the as-synthesized CoO and normalized it by its BET

SSA (**Supplementary Fig. 29c**) and ECSA (**Supplementary Fig. 29d**) to obtain the intrinsic activity. It is found that the intrinsic activity (k_{ECSA}) of $Co^{2+}{}_{Oh}$ sites is 1.5-fold higher than that of $Co^{3+}{}_{Oh}$, yet 10.1-fold lower than $Co^{2+}{}_{Td}$ (**Supplementary Figs. 29e and f**). Similar trend is also observed in comparison of k_{norm}s. This can be ascribed to the inefficient electron transfer ability of octahedrally coordinated O atoms compared to tetrahedrally coordinated O atoms in $Co^{2+}{}_{Td}$ sites.

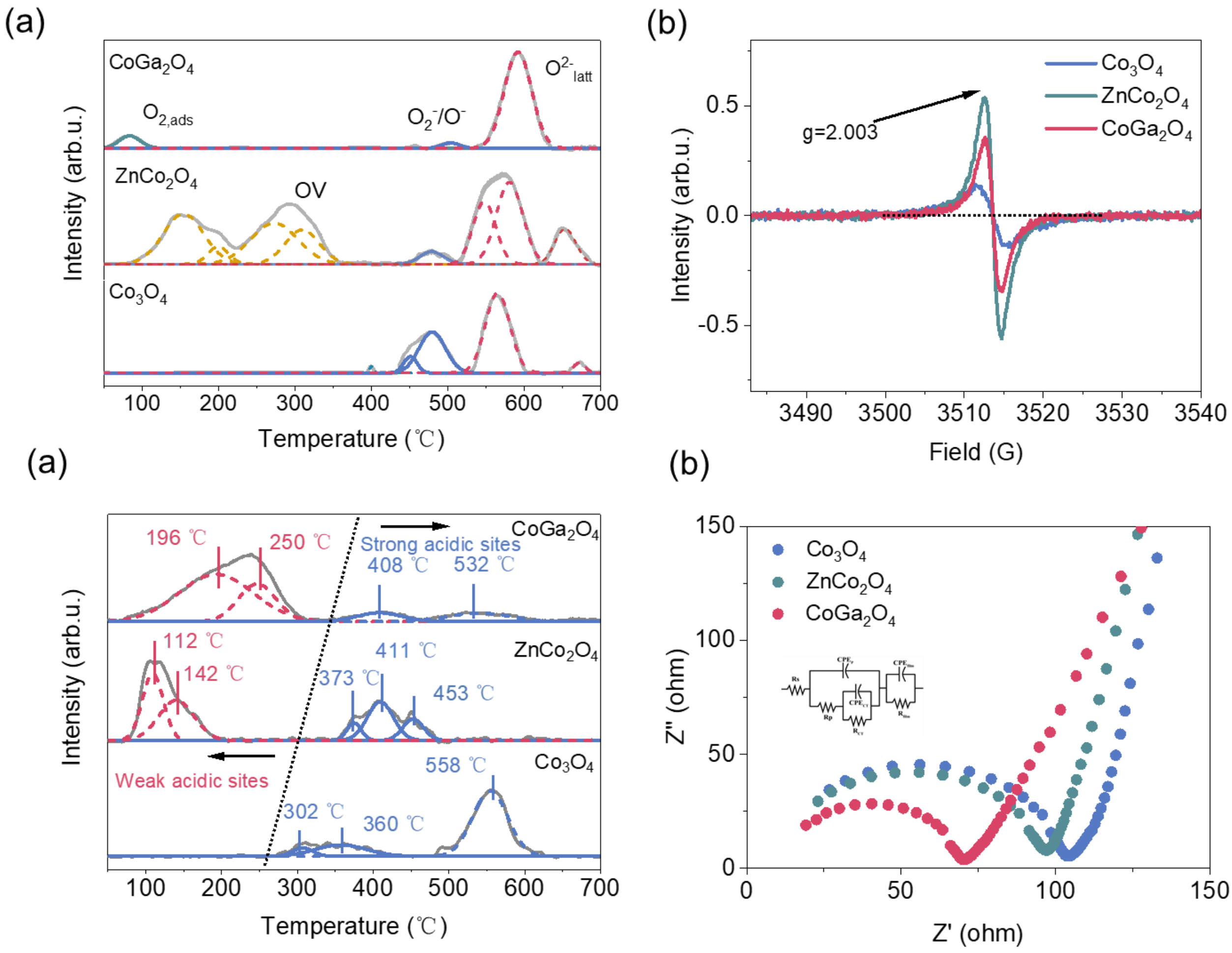

Supplementary Fig. 30 | Physicochemical properties of the as-synthesized spinel oxides. O_2-TPD profiles (**a**), Cryo-EPR spectra (**b**), NH_3-TPD profiles (**c**), and EIS spectra (**d**) of Co_3O_4, $ZnCo_2O_4$, and $CoGa_2O_4$

Cryo-EPR spectra revealed the OVs amount within the spinel oxides followed the order of $CoAl_2O_4$>$ZnCo_2O_4$>$CoGa_2O_4$>Co_3O_4 by comparing their characteristic signals with the g factor of 2.003, suggesting that geometric site substitution enhanced the formation of OVs. Similar trends in OVs degrees were also observed in O_2-TPD and XPS spectra by assessing the low temperature O_2 desorption peaks (~200 ºC) and the deconvolution results of the O *2p* surveys, respectively. Unfortunately, linear regression study suggested that OVs amount for the spinel oxides were quite irrelevant to their catalytic activities.

In NH_3-TPD spectra, the peaks located above 400°C and at around 200 °C can be ascribed to the strong and weak acidic sites, respectively[26]. Both Co^{2+}_{Td} and Co^{3+}_{Oh} geometric substitution resulted in the shift of the strong acidic sites to the weak ones. However, no strong correlations were observed between the amount of either strong acidic sites or weak acidic sties and the catalytic activities, suggesting the amount of the acidic site was not a decisive factor for the activity.

Electrical impedance spectra (EIS) suggested that geometric site substitutions can reduced the charges transfer resistances (R_{ct}s) of the spinel oxides, yet the order of R_{ct}s cannot be well correlated to that of activities.

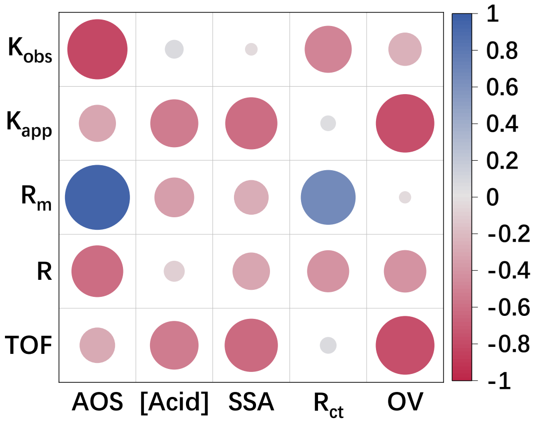

Supplementary Fig. 31 | Heat map between the coefficients of the selected properties and the activity data for correlation.

The heatmap data in was normalized using the following formula.

$$X' = \frac{X - X_{min}}{X_{max} - X_{min}} \tag{6}$$

where, X_{max} is the maximum value of sample data, X_{min} is the minimum value of sample data, X is the sample data, and X' is the normalized data.

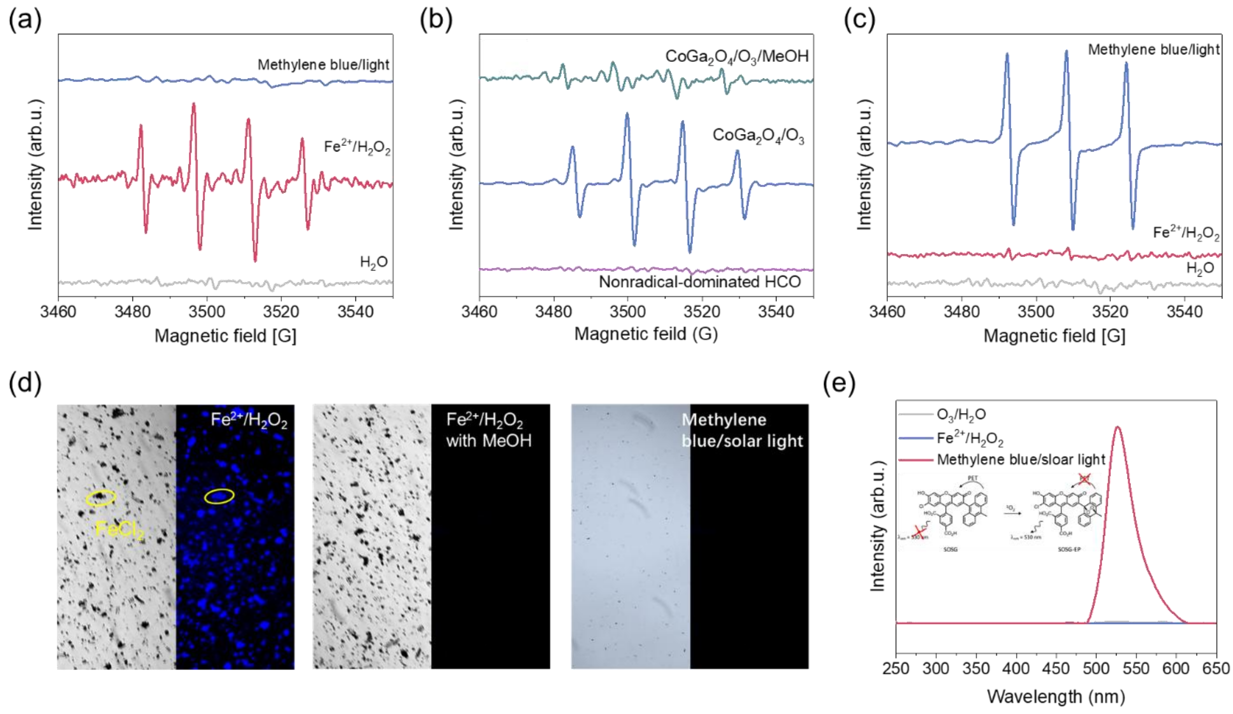

Supplementary Fig. 32 | Testing the selectivity of ROS probes. (**a**) EPR spectra with DMPO as the spin trapping agent for $^{\bullet}OH$ produced in the homogeneous Fenton system and the homogeneous dye-activated photocatalytic system; (**b**) EPR spectra with DMPO as the spin trapping agent in nonradical species-dominated heterogeneous catalytic ozonation system and effect of methanol as a quenching agent for $^{\bullet}OH$. Nonradical EPR spectra was adapted from our previous publication[27]. (**c**) EPR spectra with TEMP as the spin trapping agent for $^1O_2$ produced in the homogeneous Fenton system and the homogeneous dye-activated photocatalytic system. (**d**) Visualization of the generated $^{\bullet}OH$ on the surface of spinel oxides using coumarin as the $^{\bullet}OH$ fluorescence probe by inverted fluorescence microscopy (IFM) in the homogeneous Fenton system and the homogeneous dye-activated photocatalytic system. (**e**) Photoluminescence spectra of singlet oxygen sensor green (SOSG) as the $^1O_2$ probe in the homogeneous Fenton system and the homogeneous dye-activated photocatalytic system.

In this study, a combined strategy using the spin-trapping EPR technique and fluorescence probes was employed to investigate the types of the generated ROS. To verify the selectivity of these methods for

$^{\bullet}OH$ and $^1O_2$ detection, their characteristic signals were validated using the classic homogeneous Fenton system (Fe^{2+}/H_2O_2)[28] and the homogeneous dye-activated photocatalytic system (methylene blue/solar light)[29] that are designed for the production of $^{\bullet}OH$ and $^1O_2$ as the single type of ROS, respectively.

In the EPR tests, strong signals for DMPO-$^{\bullet}OH$ adducts, with the hyperfine splitting couplings of $a_N = a_H = 14.9$ G and intensity ratio of 1:2:2:1, were detected in the Fenton system, yet no such signals were observed in the dye-activated photocatalytic system (**Supplementary Fig. 32a**). These results suggest that DMPO serves as a spin-trapping agent selective for $^{\bullet}OH$ over $^1O_2$. Moreover, we compared the EPR signals in this study to our previously reported ones that are dominated by the nonradical species (i.e., surface-adsorbed oxygen atom and O_3) to reinforce the sensitivity of EPR spin-trapping tests (**Supplementary Fig. 32b**). In catalytic ozonation dominated by surface-adsorbed nonradical species, no such strong DMPO-$^{\bullet}OH$ adducts signals were observed and the signal intensity of the DMPO-$^{\bullet}OH$ adducts was similar to that of H_2O/O_3 system without the presence of a catalyst. For the $CoGa_2O_4/O_3$ system producing massive $^{\bullet}OH$, the addition of methanol as the $^{\bullet}OH$ quencher significantly decreased the intensity of DMPO-$^{\bullet}OH$ adducts, resulting in the formation of carbon-centered radicals, which are the oxidation products of methanol by $^{\bullet}OH$. In our EPR tests using TEMP as the spin-trapping agent, methanol (1 M) was added to eliminate the generated $^{\bullet}OH$, as $^{\bullet}OH$ can also oxidize TEMP to form TEMPO, which may mislead the results. As shown in **Supplementary Fig. 32c**, strong triplet signals with an intensity ratio of 1:1:1 were observed in the methylene blue/solar light photocatalytic system; however, no such signals were detected in the Fenton system. Furthermore, a higher intensity of triplet signals in the $ZnCo_2O_4/O_3$ system than that in the Co_3O_4/O_3 system suggested a greater production of $^1O_2$.

In the tests utilizing fluorescence probes, coumarin was selected as the $^{\bullet}OH$ probe to detect the produced 7-hydroxycoumarin (7-HC) as the selectively oxidation product, whose fluorescence can be observed by

an inverted fluorescence microscope. Strong fluorescence signals of 7-HC were observed on the partially dissolved $FeCl_2$ solid in the Fenton system, whereas no significant fluorescence was detected in the $^1O_2$-generating methylene blue/solar light photocatalytic system (**Supplementary Fig. 32d**). The addition of methanol as the $^•OH$ scavenger quenched the blue fluorescence signals of 7-HC generated in the Fenton system. As a highly selective probe for $^1O_2$, singlet oxygen sensor green (SOSG) can be oxidized into the fluorescence form by $^1O_2$ with an excitation wavelength of 504 nm and an emission wavelength of 525 nm. Similar observations to those of coumarin-based results were noted in the application of SOSG for $^1O_2$ detection (**Supplementary Fig. 32e)**. According to the fluorescence spectra, marginal signal responses were observed in the Fenton system and O_3/H_2O system (without a catalyst), however, a strong fluorescence signal was detected in the methylene blue/UV photocatalytic system. This strong deviation confirms the high selectivity of SOSG as a fluorescence probe for distinguishing $^1O_2$ from $^•OH$.

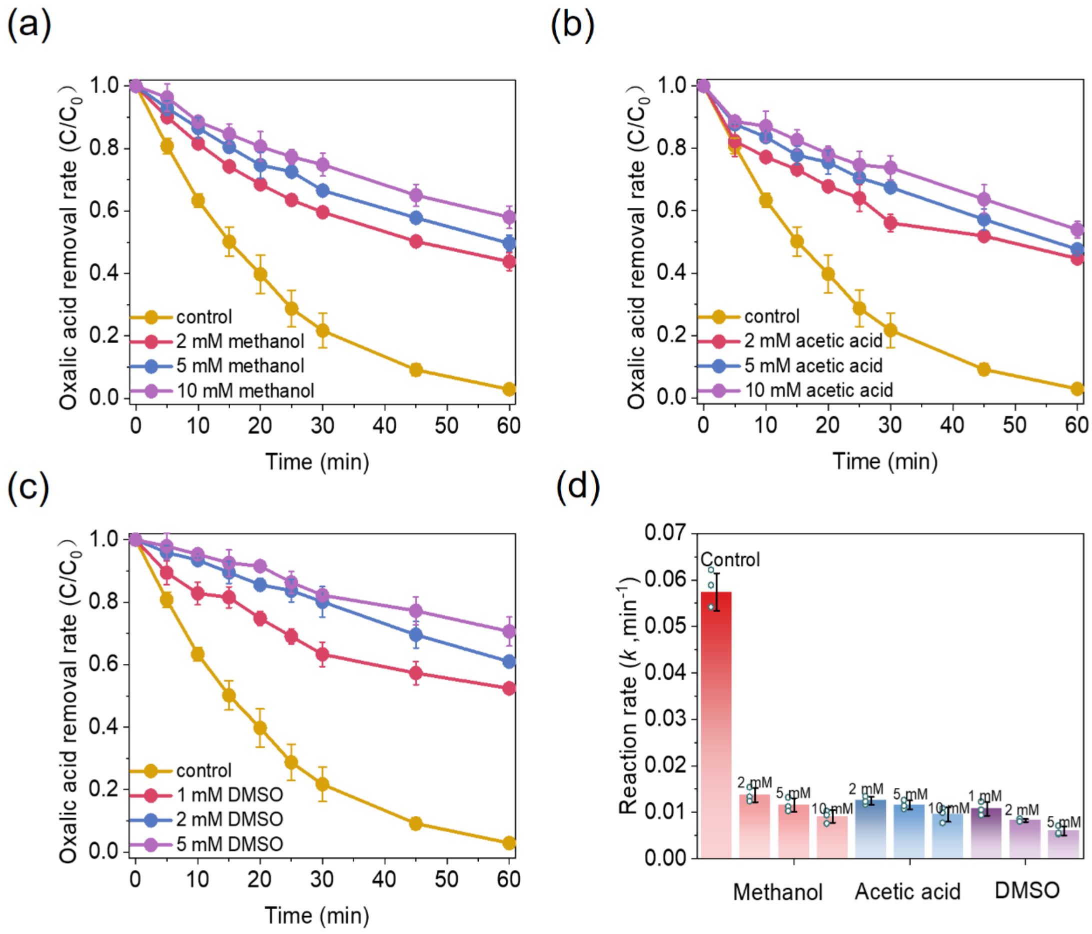

Supplementary Fig. 33 | Quenching tests in catalytic ozonation by Co_3O_4 utilizing methanol (MeOH) **(a)**, Acetic acid (AA) **(b)**, and dimethyl sulfoxide (DMSO) **(c)** as quenching agents. **(d)** Pseudo first-order rate constants of OA degradation quenching tests in catalytic ozonation for Co_3O_4. Catalyst loading: 0.1 g L^{-1}; temperature: 25 °C; ozone flow rate: 100 mL min^{-1}; ozone concentration: 25 mg L^{-1}; initial pH was adjusted to 3 by adding 0.01 M H_2SO_4/NaOH in OA solution. $[OA]_0$: 50 mg L^{-1}. Error bars are standard error values of three tests (n = 3). Source data are provided as a Source Data file.

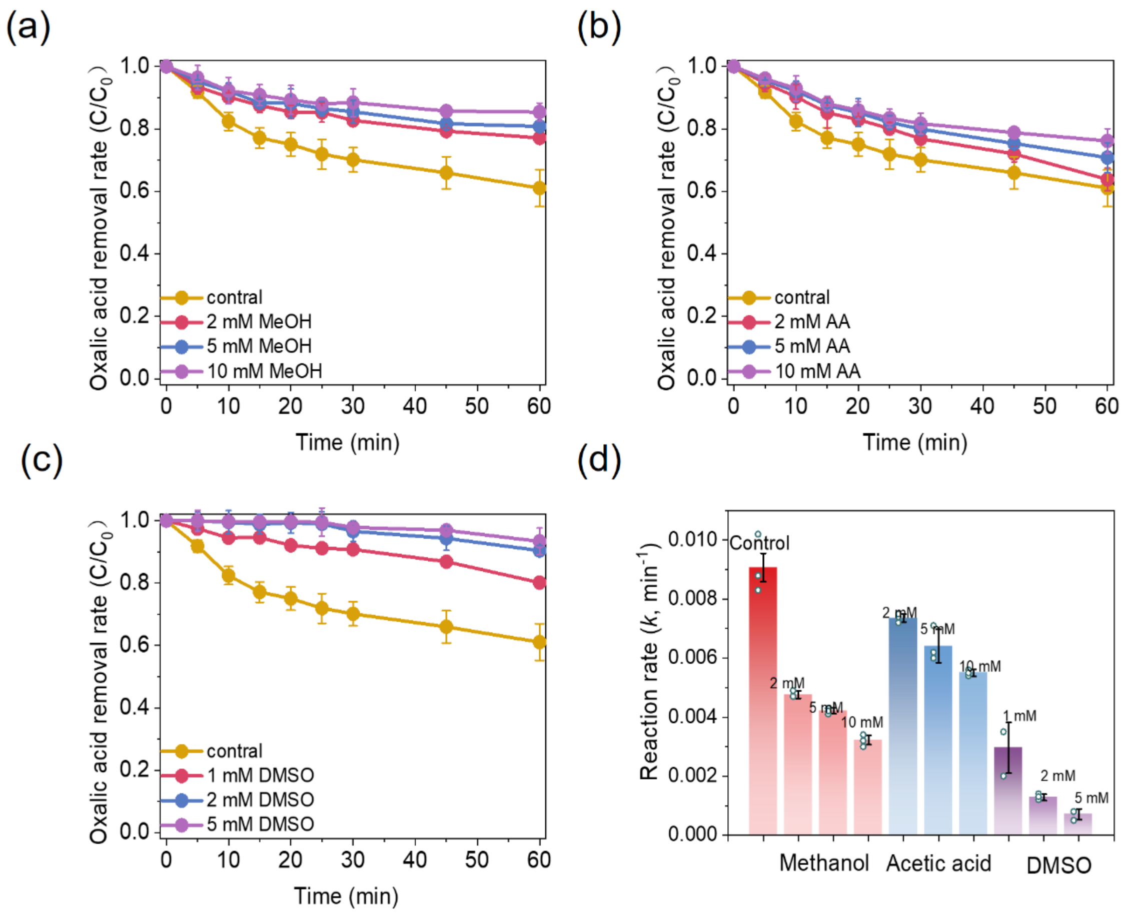

Supplementary Fig. 34 | Quenching tests in catalytic ozonation by $ZnCo_2O_4$ utilizing MeOH **(a)**, AA **(b)**, and DMSO **(c)** as quenching agents. (d) Pseudo first-order rate constants of OA degradation quenching tests in catalytic ozonation for $ZnCo_2O_4$.Catalyst loading: 0.1 g L^{-1}; temperature: 25 °C; ozone flow rate: 100 mL min^{-1}; ozone concentration: 25 mg L^{-1}; initial pH was adjusted to 3 by adding 0.01 M H_2SO_4/NaOH in OA solution. $[OA]_0$: 50 mg L^{-1}. Error bars are standard error values of three tests (n = 3). Source data are provided as a Source Data file.

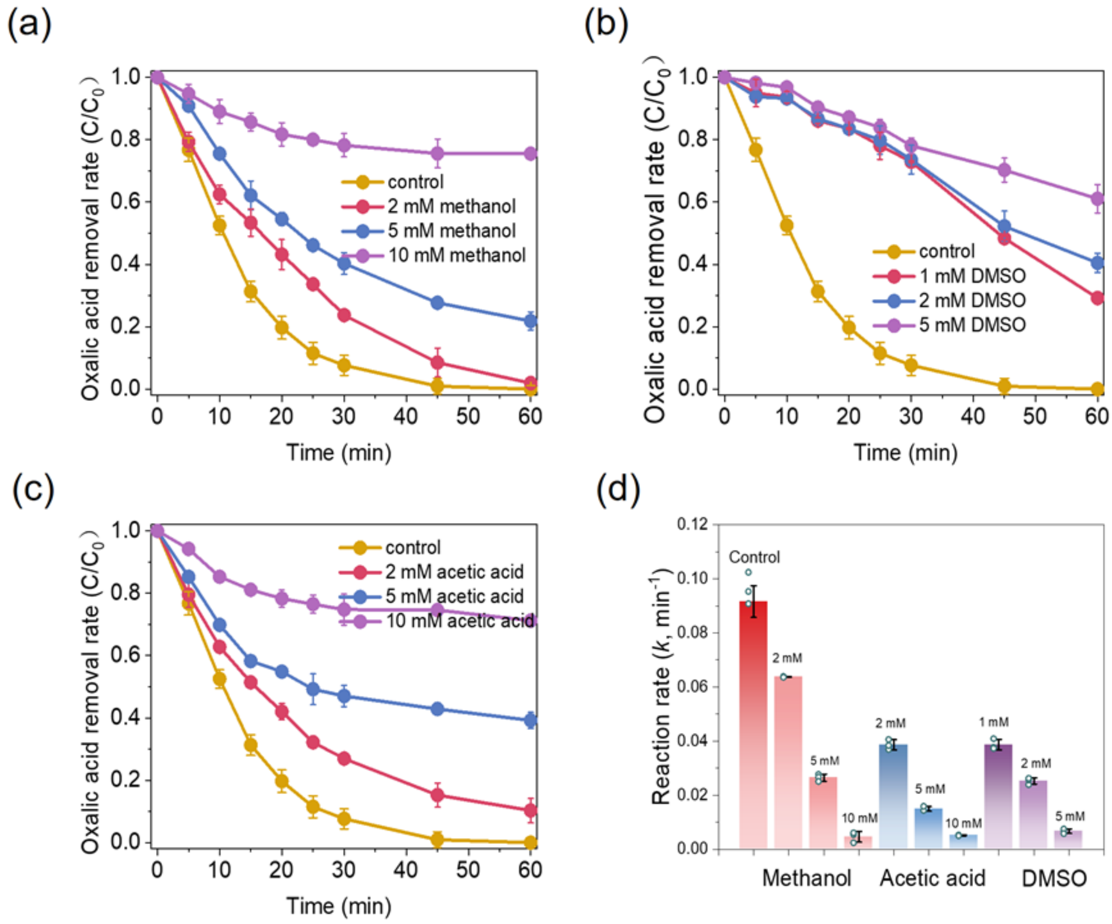

Supplementary Fig.35 | Quenching tests in catalytic ozonation by $CoGa_2O_4$ utilizing MeOH **(a)**, AA **(b)**, and DMSO **(c)** as quenching agents. **(d)** Pseudo first-order rate constants of OA degradation quenching tests in catalytic ozonation for $CoGa_2O_4$. Catalyst loading: 0.1 g L^{-1}; temperature: 25 °C; ozone flow rate: 100 mL min^{-1}; ozone concentration: 25 mg L^{-1}; initial pH was adjusted to 3 by adding 0.01 M H_2SO_4/NaOH in OA solution. $[OA]_0$: 50 mg L^{-1}. Error bars are standard error values of three tests (n = 3). Source data are provided as a Source Data file.

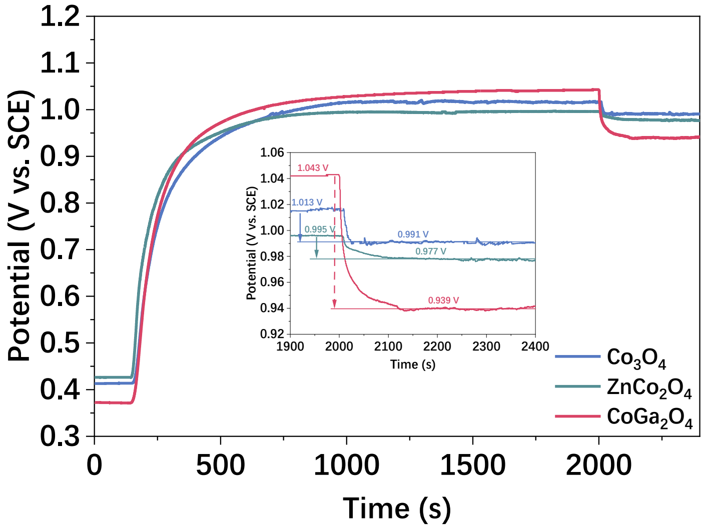

Supplementary Fig. 36 | Open-circuit potential curves of the various spinel oxides.

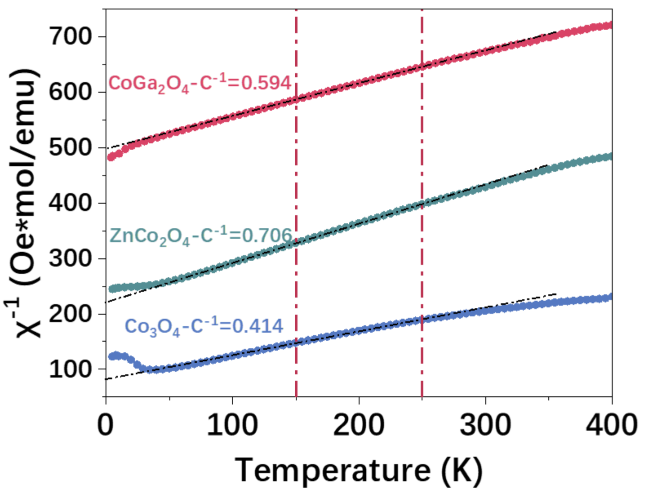

Supplementary Fig. 37 | Temperature-dependent inverse susceptibilities fitted by the Curie–Weiss law over different spinel perovskites.

Spin states of the as-synthesized spinel oxides were examined by fitting the SQUID results according to Curie-Weiss law (χ^{-1}-T fitting) because spin state of spinel oxides might act as a crucial factor determining the activities. The effective magnetic moment (μ_{eff}) can be calculated from the following equation:

$$\mu_{eff} = \sqrt{8C}\,\mu_B \tag{7}$$

where, μ_B is the Bohr magneton.

As illustrated in **Supplementary Table 12**, $Co^{2+}{}_{Td}$ within the spinel oxides was in the high spin state ($e^4t_2^3$), while $Co^{3+}{}_{Oh}$ was in the intermediate spin state ($t_{2g}^5e_g^1$). Therefore, Co_3O_4 and its geometrical site substitutes with fixed (111) facet were constructed for simulation and magnetic moments of $Co^{2+}{}_{Td}$ and $Co^{3+}{}_{Oh}$ were set as high spin state and intermediate spin state, respectively.

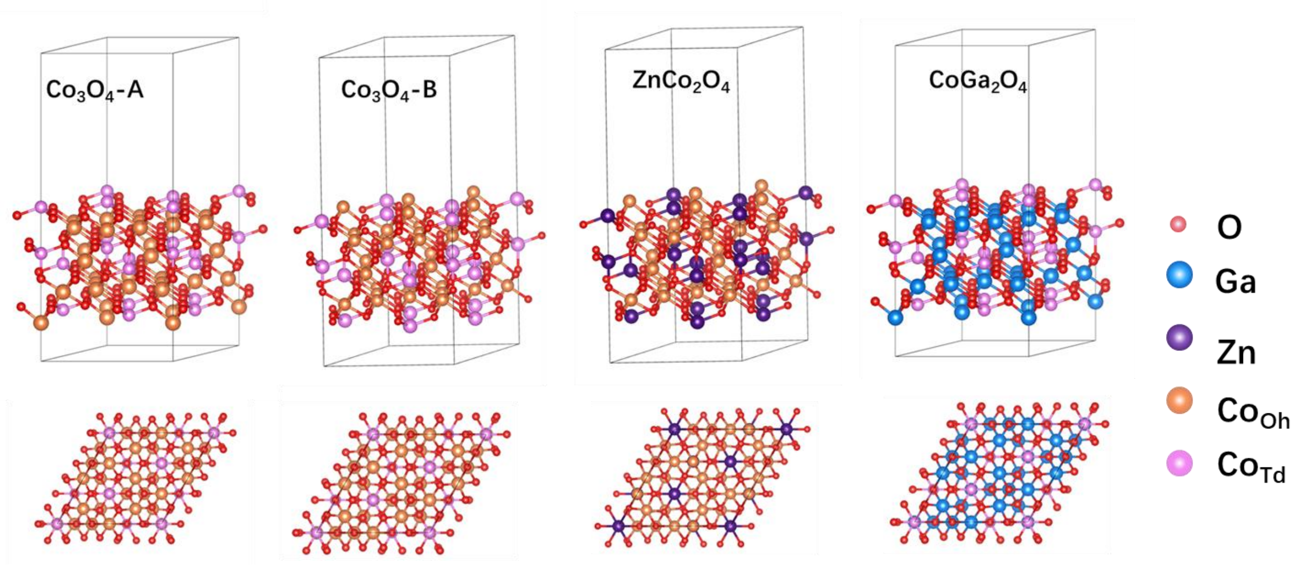

Supplementary Fig. 38 | Optimized models for DFT calculation. Constructed simulation models with [111] facet. Co_3O_4 with exposure of $Co^{2+}{}_{Td}$ and $Co^{3+}{}_{Oh}$ sites; $ZnCo_2O_4$ with exposure of $Co^{3+}{}_{Oh}$ sites; and $CoGa_2O_4$ with exposure of $Co^{2+}{}_{Td}$ sites of spinel oxides (111).

In this study, although Al^{3+} and Mg^{2+} were employed as the $Co^{3+}{}_{Oh}$- and $Co^{2+}{}_{Td}$- sites substitutes within the Co_3O_4 structure accordingly, their empty-filled *3d* orbitals cannot induce the *d-p* orbital hybridizations with the adsorbed O species. Contrarily, *p-p* orbital interactions are expected, which quite deviate from the scenarios occurred to $Co^{3+}{}_{Oh}$- and $Co^{2+}{}_{Td}$- sites in Co_3O_4[30]. In addition, the much higher SSAs of $CoAl_2O_4$ and $MgCo_2O_4$ than the other spinel oxides (**Supplementary Table 6**) together with the heavily aggregated morphology hindering the exposure of [111] facet fail the precise comparisons. Therefore, the subsequent mechanistic investigations mainly focus on Co_3O_4 and its geometrical-site substitutes with fully-occupied inert *3d* orbital electrons and comparable SSAs (i.e., $CoGa_2O_4$ and $ZnCo_2O_4$) with the same exposure of [111] facet.

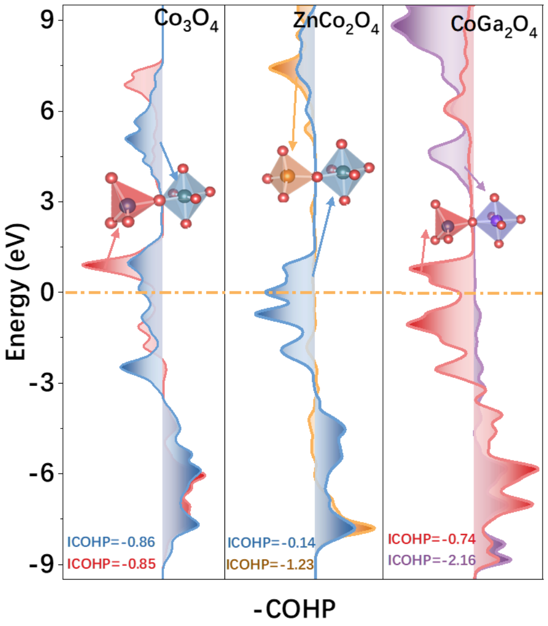

Supplementary Fig. 39 | Calculation of bonding strength via ICOHP analysis. COHP plots and -ICOHP values of interatomic bonds near the spinel-catalysts interfaces (red area: $Co^{2+}{}_{Td}$-O, blue area: $Co^{3+}{}_{Oh}$-O, orange area: $Zn^{2+}{}_{Td}$-O, and purple area: $Ga^{3+}{}_{Oh}$-O).

In spinel oxide crystal structures, substitution of the host cations by the foreign cations can result in the covalency competition between the M_{Td}-O (metal-oxygen bond in tetrahedral unit) and M_{Oh}-O (metal-oxygen bond in octahedral unit) in the M_{Td}-O-M_{Oh} backbone. The bond breakage occurring at the weaker bond in M_{Td}-O-M_{Oh} backbone gives rise to the exposure of the non-oxygen-bonding metal (M-), which is usually considered as the active site in oxygen-involved catalysis. Crystal orbital Hamilton populations (COHPs) analysis was performed to evaluate this covalency competition within the geometrical site substitutions, in which the negative and positive of the COHPs values suggest the bonding and anti-bonding interaction between the electronic states. $Co^{2+}{}_{Td}$ and $Co^{3+}{}_{Oh}$ within the pristine Co_3O_4 obtained a similar anti-bonding interaction near the Fermi level. Substituting $Co^{2+}{}_{Td}$ by Zn^{2+} altered the anti-bonding interaction, making $Co^{3+}{}_{Oh}$ site as the predominant one while trace interaction was observed for Zn^{2+}. A

similar scenario occurred to $CoGa_2O_4$, illustrating that Co^{2+}_{Td} became the dominant anti-bonding interaction site. Therefore, substitution either the Co^{2+}_{Td} or Co^{3+}_{Oh} site by Zn^{2+} or Ga^{3+} diminished the bonding strength of the Co-O. The integrated COHPs values from the lowest energy considered up to the Fermi level (ICOHP) were further derived to qualitatively measure the bonding strength, in which the larger negative ICOHP (-ICOHP) correlates to the stronger bond strength. The calculated -ICOHP values for Zn^{2+}_{Td}-O and Co^{3+}_{Oh}-O in $ZnCo_2O_4$, Co^{2+}_{Td} and Ga^{3+} in $CoGa_2O_4$ were 0.14 and 1.23, 0.74, and 2.16 eV, respectively. The decreased bonding strength for Co^{3+}_{Oh}-O in $ZnCo_2O_4$ and Co^{2+}_{Td}-O in $CoGa_2O_4$ validated that Co^{3+}_{Oh} and Co^{2+}_{Td} as the preferentially exposed active sites in $ZnCo_2O_4$ and $CoGa_2O_4$ in catalytic ozonation reactions accordingly. For pristine Co_3O_4, the similar -ICOHP values for Co^{2+}_{Td} and Co^{3+}_{Oh} suggested that identical exposure possibility of these two sites in catalytic reactions[18, 31].

This favored the exposure of the unsubstituted cations Co^{2+}_{Td} and Co^{3+}_{Oh} as the preferentially exposed active sites in $CoGa_2O_4$ and $ZnCo_2O_4$, respectively, which agreed well with the EXAFS analysis.

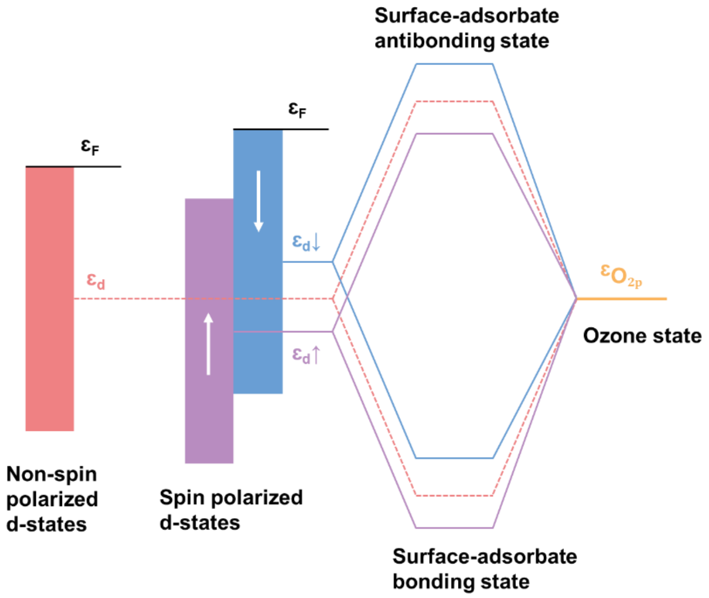

Supplementary Fig. 40 | Schematic illustration of the comparison of the coupling of an adsorbate level $\varepsilon_{O_{2p}}$ with the metal *d*-states characterized by a single *d*-band center (dotted line, ε_d for the non-spin-polarized case) and two *d*-band centers ($\varepsilon_{d\uparrow}$ and $\varepsilon_{d\downarrow}$ represent the spin-up and spin-down centers accordingly for the spin-polarized case)[32]. Adapted with permission from ref [32]. Copyright 2016. Springer Nature.

Two sets of bonding molecular orbitals (MOs) and anti-bonding molecular orbitals (MO*s) can be generated for both spin-up and spin-down channels when they interact with the O *2p* orbitals from O_3. It is suggested that the elevated energy level of 3d orbitals facilitates the injection of their electrons into the MO*s[33].

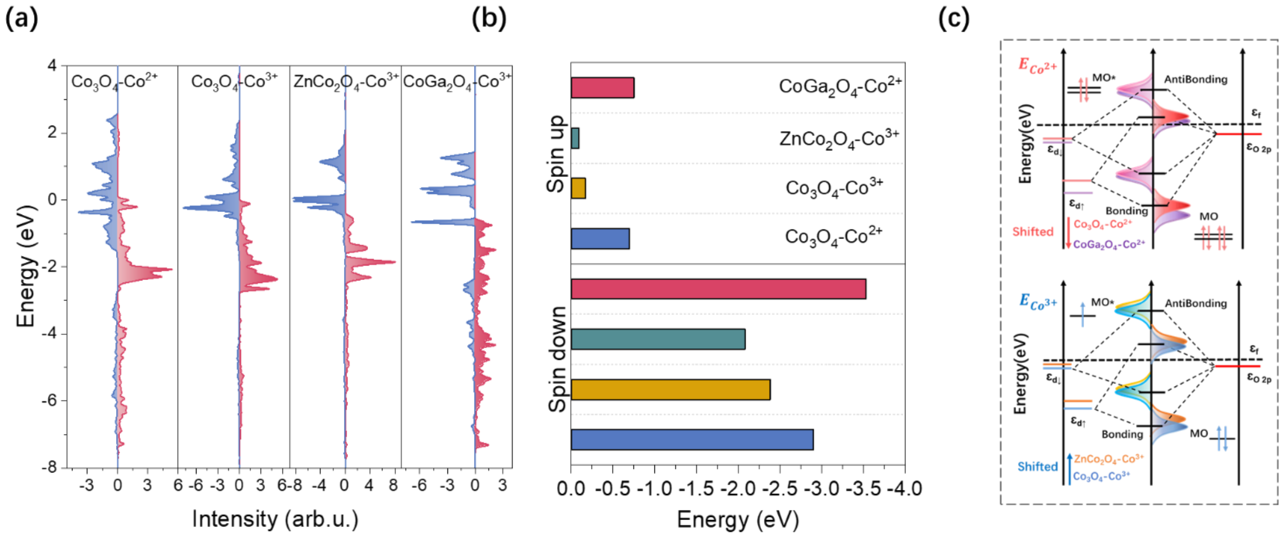

Supplementary Fig. 41 | *D*-band centers analysis and bonding behaviors of different spin channels. **(a)** Projected electronic density of states (PDOS) analysis on Co *3d* orbitals for Co_3O_4-Co^{2+}_{Td}, $CoGa_2O_4$-Co^{2+}_{Td}, Co_3O_4-Co^{3+}_{Oh}, and $ZnCo_2O_4$-Co^{3+}_{Oh}. **(b)** Calculated *d*-band center of spinel oxides. **(c)** Schematic bond formation between the reaction surface and the adsorbate.

Bonding behaviors arising from intrinsic geometrical site differences were investigated by projected electronic density of states (PDOS) analysis. Strong spin polarization was observed for both Co^{2+}_{Td} and Co^{3+}_{Oh} sites in Co_3O_4 and their substitutes stemming from the magnetism of the unpaired electrons.

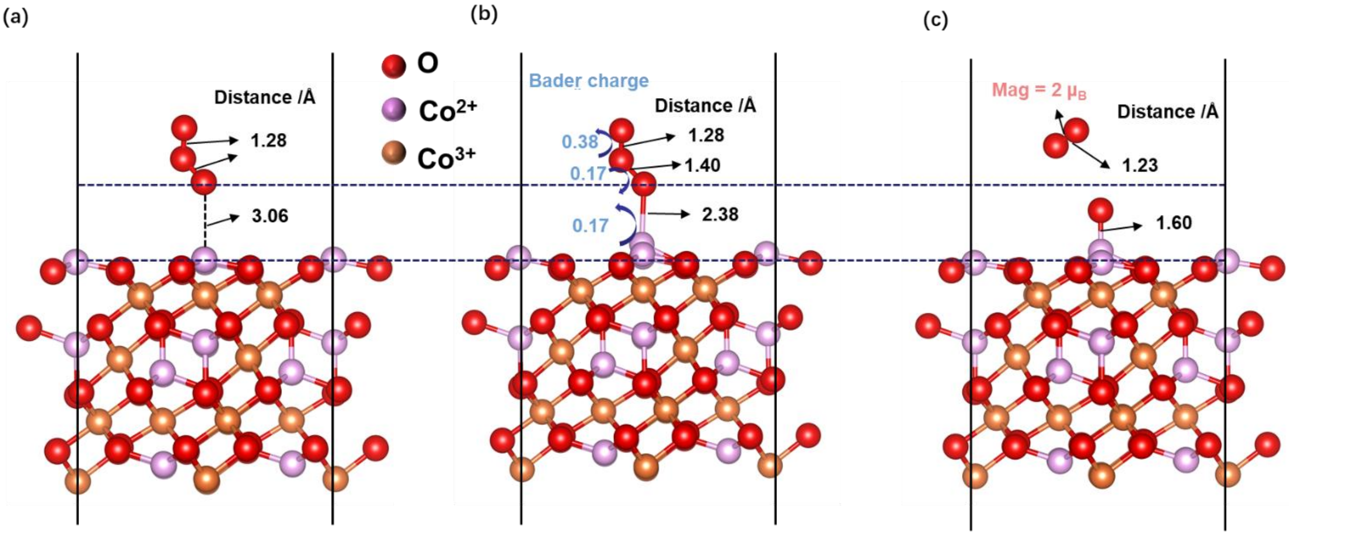

Supplementary Fig. 42 | Interactions between O_3 and Co^{2+}_{Td} site of Co_3O_4 [111] at initial **(a)**, intermediate **(b)**, and final **(c)** adsorption states. The bond length, Bader charge, and magnetic moments are labelled.

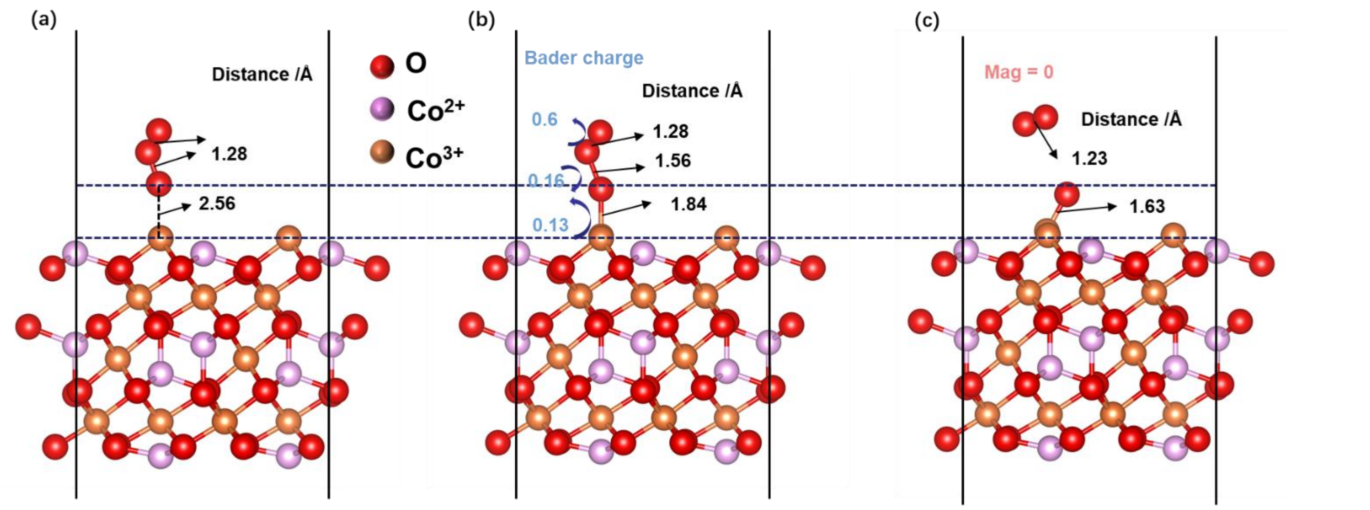

Supplementary Fig. 43 | Interactions between O_3 and Co^{3+}_{Oh} site of Co_3O_4 [111] at initial **(a)**, intermediate **(b)**, and final **(c)** adsorption states. The bond length, Bader charge, and magnetic moments are labelled.

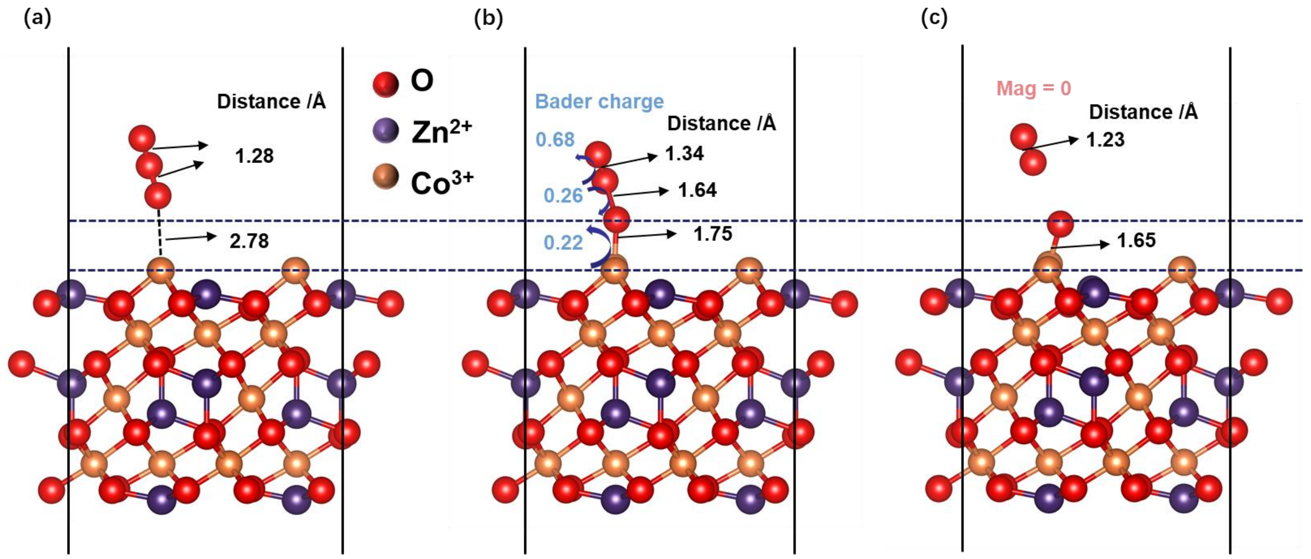

Supplementary Fig. 44 | Interactions between O_3 and Co^{2+}_{Td} site of $ZnCo_2O_4$ [111] at initial **(a)**, intermediate **(b)**, and final **(c)** adsorption states. The bond length, Bader charge, and magnetic moments are labelled.

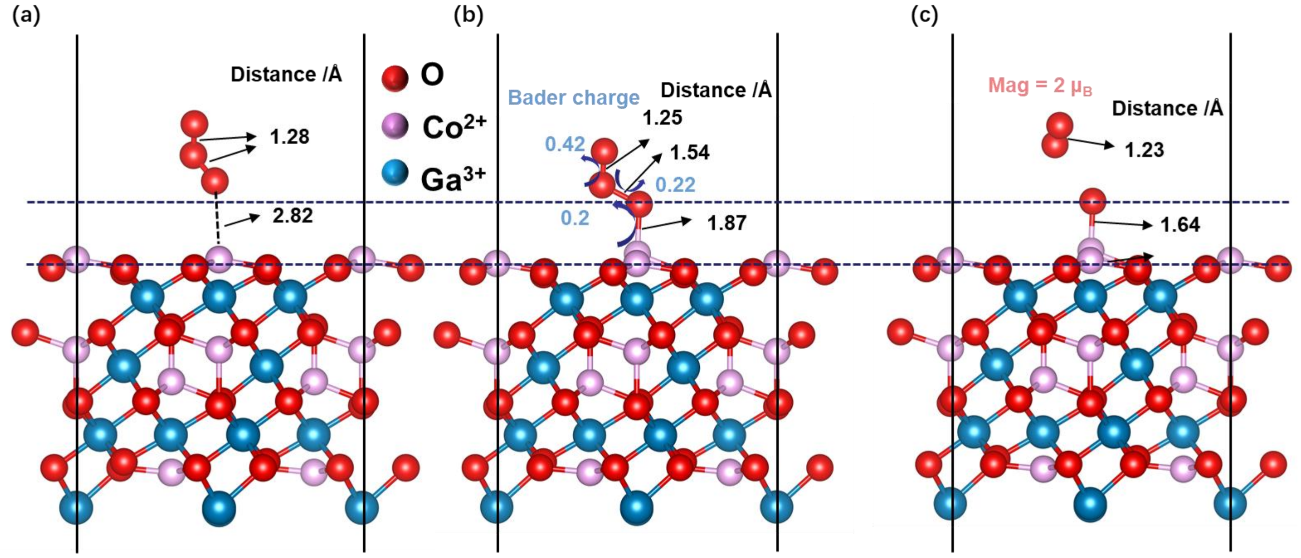

Supplementary Fig. 45 | Interactions between O_3 and Co^{3+}_{Oh} site of $CoGa_2O_4$ [111] at initial **(a)**, intermediate **(b)**, and final **(c)** adsorption states. The bond length, Bader charge, and magnetic moments are labelled.

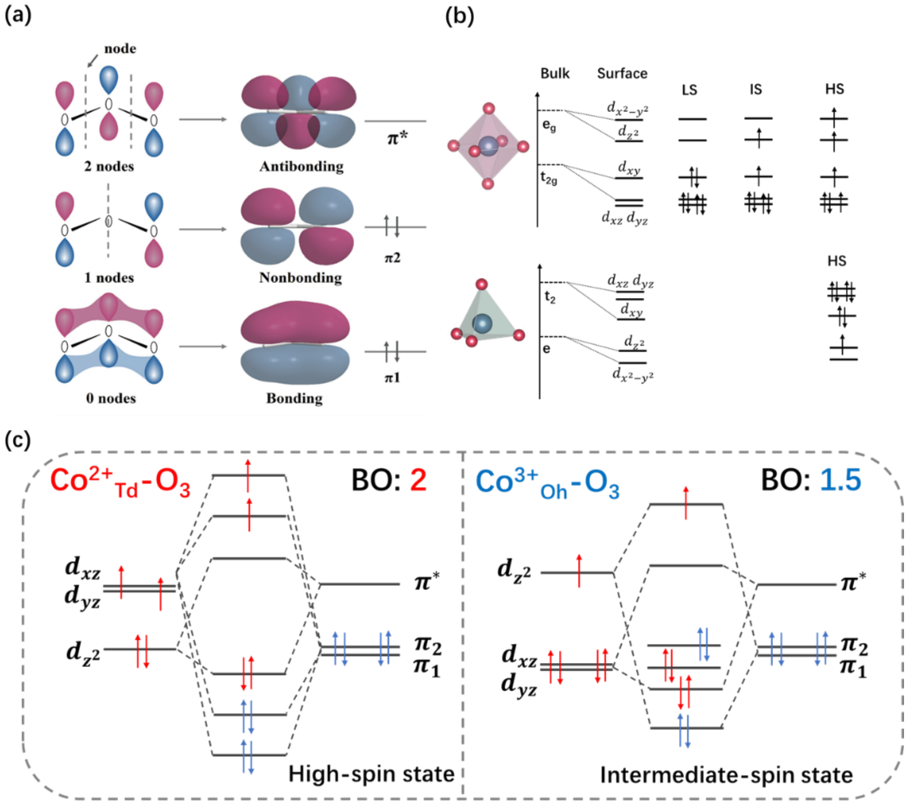

Supplementary Fig. 46 | Analysis of the molecular orbitals of ozone and their bonding behaviors with different Co sites. (a) Bonding orbitals and antibonding molecular orbitals of ozone molecules obtained by molecular orbital theory[34]. **(b)** *D*-electron configurations of Co^{2+}_{Td} sites and Co^{3+}_{Oh} sites in different spin state. **(c)** The orbital interactions between $Co^{2+}_{Td}/Co^{3+}_{Oh}$ centers and O_3 molecules.

To calculate the bond order, the orbital splitting and electron filling behaviors of Co^{2+} and Co^{3+} are analyzed based on the theoretical calculation and magnetic property results. Co^{2+} and Co^{3+} exhibit the spin state ($e^4t_2^3$) and intermediate spin state ($t_{2g}^5e_g^1$), respectively. Given the high symmetrical conservation, both $d_{x^2-y^2}$ and d_{xy} orbitals do not involve in the bonding process [35]. Therefore, only the orbitals of d_{z^2}, d_{yz}, and d_{xz} might be involved in the bonding between cobalt *3d* orbitals and the O *2p* orbital from the adsorbed oxygen species. Note that the frontier orbitals in the molecular orbital

explanation of ozone comprise two bonding orbitals (π_1&π_2) and one anti bonding orbital (π^*), respectively. The frontier of ozone orbitals interacts with the Co^{2+}/Co^{3+} valence orbitals to form bonds. **Supplementary Fig. 40c** schematically illustrates the orbital splitting and electron filling behaviors of Co^{2+} and Co^{3+} and their bonding interactions with O *2p*. The bond order (BO) can be calculated by the following equation.

$$\mathrm{BO}=\frac{\textit{number of bonding electrons-number of antibonding electrons}}{2} \tag{8}$$

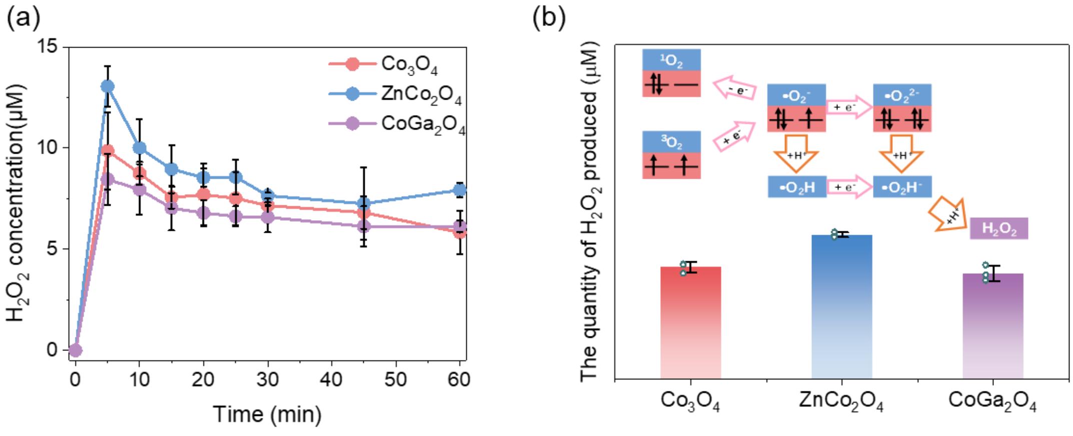

Supplementary Fig. 47 | Testing the produced H_2O_2 concentration for different O_3/spinel systems. **(a)** Time-dependent H_2O_2 generation in different spinel oxides systems. **(b)** Cumulative H_2O_2 generation. Reaction conditions: Catalyst loading: 0.1 g L^{-1}; temperature: 25 °C; ozone flow rate: 100 mL min^{-1}; ozone concentration: 25 mg L^{-1}; initial pH was adjusted by adding 0.01 M H_2SO_4/NaOH in OA solution. $[OA]_0$: 50 mg L^{-1}. Error bars are standard error values of three tests (n = 3). Source data are provided as a Source Data file.

Superoxide radicals ($O_2^{\bullet-}$) play a crucial intermediary role in the electron transfer process during O_3 activation, relating the selection of ozone activation pathways. However, the high reaction kinetics between the $O_2^{\bullet-}$ quenching agents and O_3 bring challenges in accurately determining its concentration by quenching tests. Therefore, the amount of $O_2^{\bullet-}$ was measured indirectly by evaluating the concentration of the produced H_2O_2, a conversion intermediate for $O_2^{\bullet-}$. The higher amounts of H_2O_2 formed for $ZnCo_2O_4$ than those for $CoGa_2O_4$ further revealed that a more complexed reaction process for O_3 activation might be involved in $Co^{2+}{}_{Td}$ substituted spinel oxides, which decreased the ozone utilization efficiency for $^{\bullet}OH$ generation.

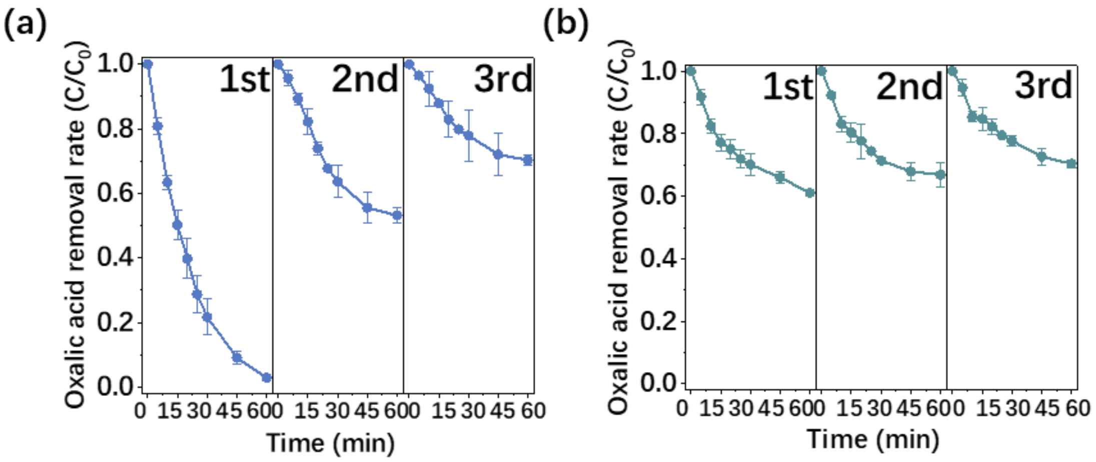

Supplementary Fig. 48 | Catalyst reusability tests for Co_3O_4 **(a)** and $ZnCo_2O_4$ **(b)**. Reaction conditions: Catalyst loading: 0.1 g L^{-1}; temperature: 25 °C; ozone flow rate: 100 mL min^{-1}; ozone concentration: 25 mg L^{-1}; initial pH was adjusted by adding 0.01 M H_2SO_4/NaOH in OA solution. $[OA]_0$: 50 mg L^{-1}. Catalyst was washed by pure water 3 times after each cycle. Error bars are standard error values of three tests (n = 3). Source data are provided as a Source Data file.

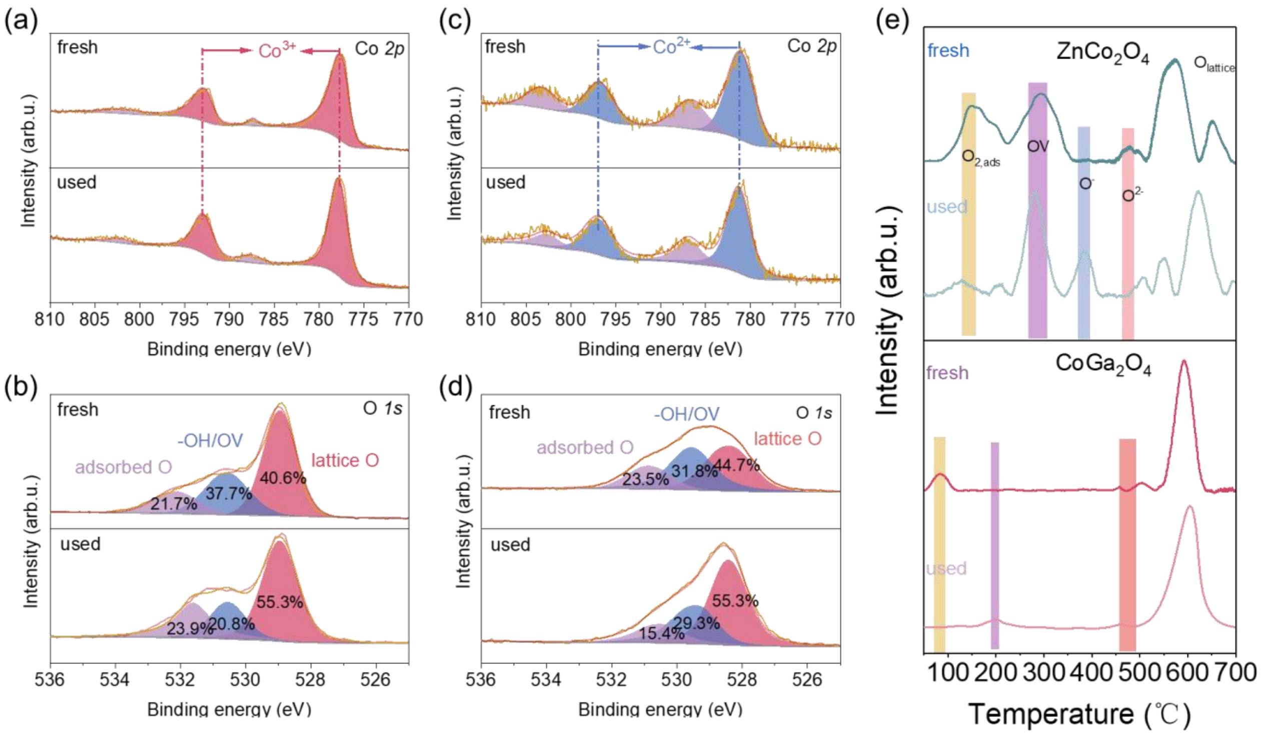

Supplementary Fig. 49 | Comparisons of the physiochemical properties of $ZnCo_2O_4$ and $CoGa_2O_4$ before and after use. High resolution XPS spectra on Co *2p* for $ZnCo_2O_4$ **(a)** and $CoGa_2O_4$ **(c),** High resolution XPS spectra on O *1s* for $ZnCo_2O_4$ **(b)** and $CoGa_2O_4$ **(d)**. **(e)** O_2-TPD profiles of $ZnCo_2O_4$ and $CoGa_2O_4$.

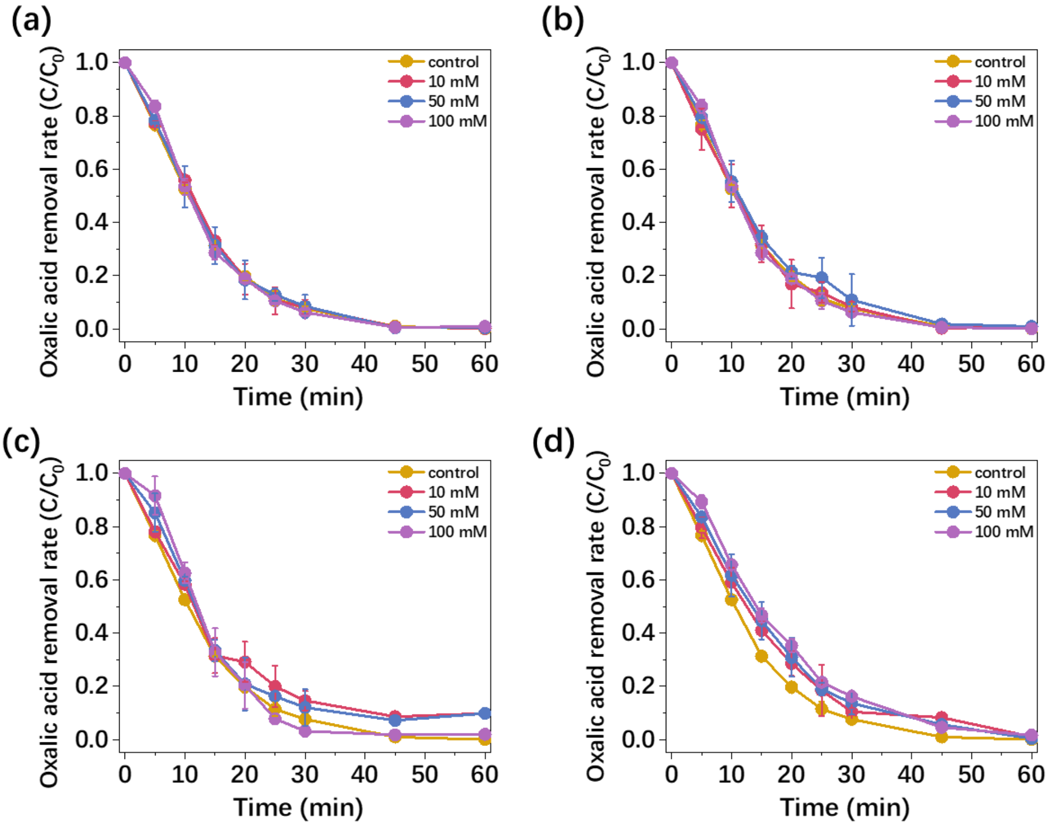

Supplementary Fig. 50 | Effects of anions on OA removal of O_3/$CoGa_2O_4$ system (**(a)** NaCl, **(b)** $NaClO_4$, **(c)** $NaNO_3$ and **(c)** Na_2SO_4). Catalyst loading: 0.1 g L^{-1}; temperature: 25 °C; ozone flow rate: 100 mL min^{-1}; ozone concentration: 25 mg L^{-1}; initial pH was adjusted to 3 by adding 0.01 M H_2SO_4/NaOH in OA solution. $[OA]_0$: 50 mg L^{-1}. Error bars are standard error values of three tests (n = 3). Source data are provided as a Source Data file.

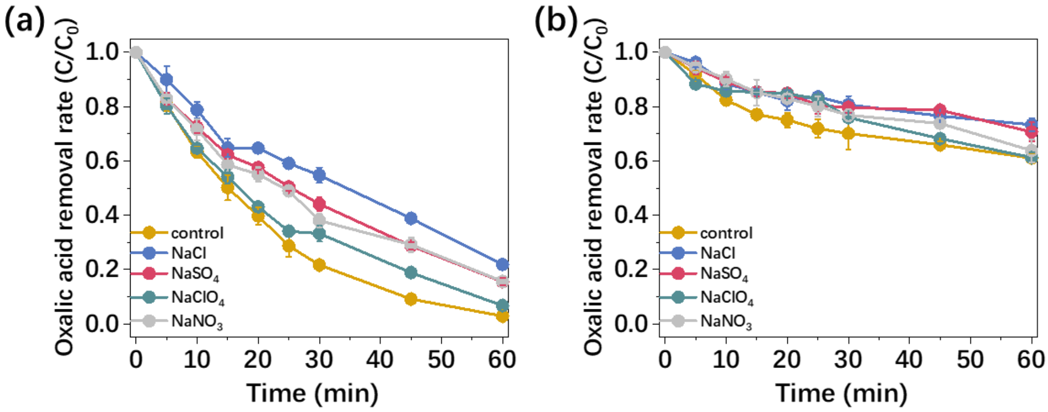

Supplementary Fig. 51| Effects of anions on OA removal of different O_3/spinel oxides (**(a)** Co_3O_4 and **(b)** $ZnCo_2O_4$) system. Catalyst loading: 0.1 g L^{-1}; temperature: 25 °C; ozone flow rate: 100 mL min^{-1}; ozone concentration: 25 mg L^{-1}; initial pH was adjusted to 3 by adding 0.01 M H_2SO_4/NaOH in OA solution. $[NaCl]_0$: 100 mmol L^{-1}, $[Na_2SO_4]_0$: 100 mmol L^{-1}, $[NaClO_4]_0$: 100 mmol L^{-1}, $[NaNO_3]_0$: 100 mmol L^{-1} $[OA]_0$: 50 mg L^{-1}. Error bars are standard error values of three tests (n = 3). Source data are provided as a Source Data file.

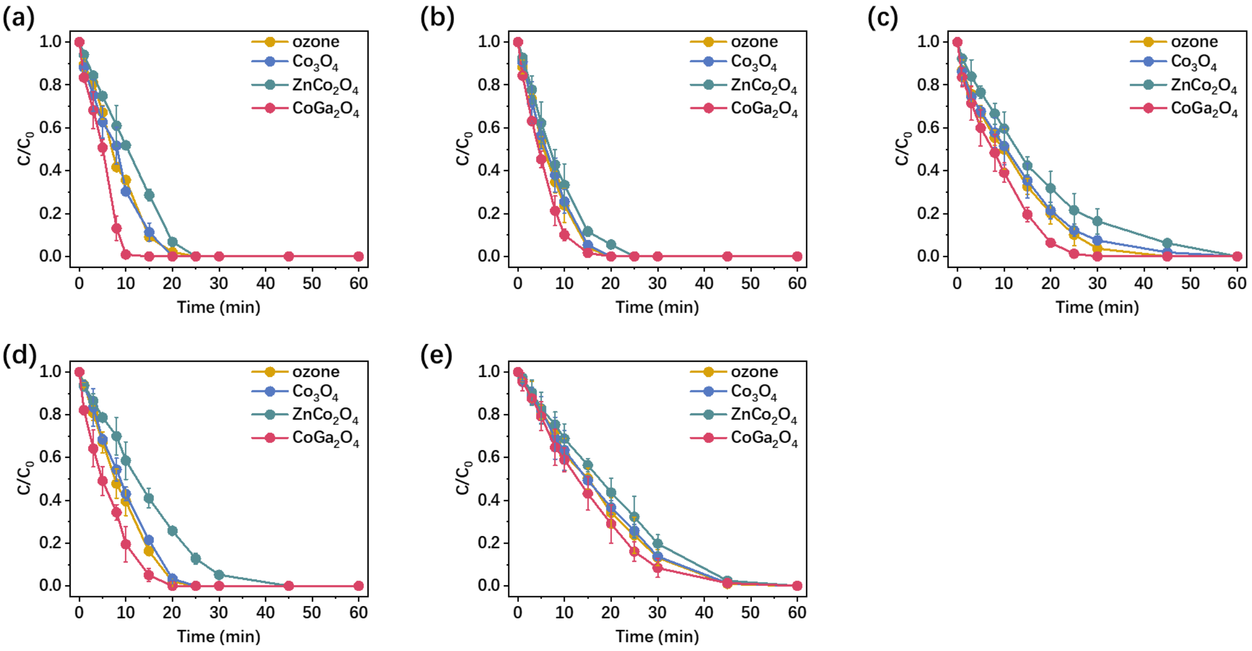

Supplementary Fig. 52 | Catalytic ozonation tests for different target pollutants. Degradation of different phenolics (**(a)** phenol, **(b)** p-CP, **(c)** pHBA, **(d)** p-NP and **(e)** BA of O_3/spinel oxides system. Catalyst loading: 0.1 g L^{-1}; temperature: 25 °C; ozone flow rate: 100 mL min^{-1}; ozone concentration: 25 mg L^{-1}; initial pH was adjusted to 3 by adding 0.01 M H_2SO_4/NaOH in solution. $[phenolics]_0$: 50 mg L^{-1}.

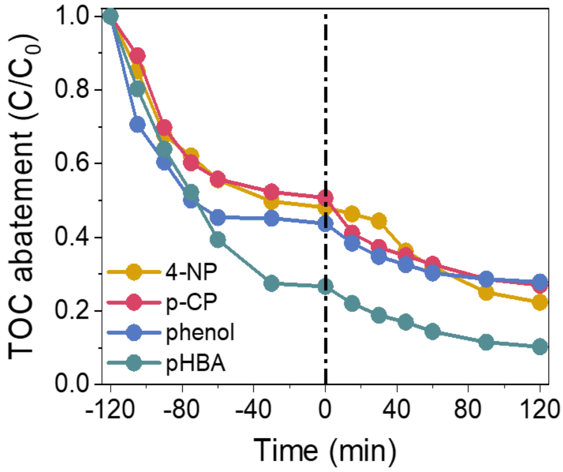

Supplementary Fig. 53 | TOC removal profiles of different phenolic contaminants by catalytic ozonation using $CoGa_2O_4$ as the catalyst. Catalyst loading: 0.1 g L^{-1}; temperature: 25 °C; ozone flow rate: 100 mL min^{-1}; ozone concentration: 25 mg L^{-1}; initial pH was adjusted to 3 by adding 0.01 M H_2SO_4/NaOH in methylene blue solution. [methylene blue]$_0$: 25 mg L^{-1}. [TOC]$_0$: 25.5 mg/L. Ozone was continuously injected in the TOC test 2 h before dosing catalysts. [p-nitrobenzene (p-NP)]$_0$: 25 mg L^{-1}; [para-Chlorophenol (p-CP)]$_0$: 25mg L^{-1}; [phenol]$_0$: 25 mg L^{-1}; [p-hydroxybenzoic acid (pHBA)]$_0$: 25 mg L^{-1}.

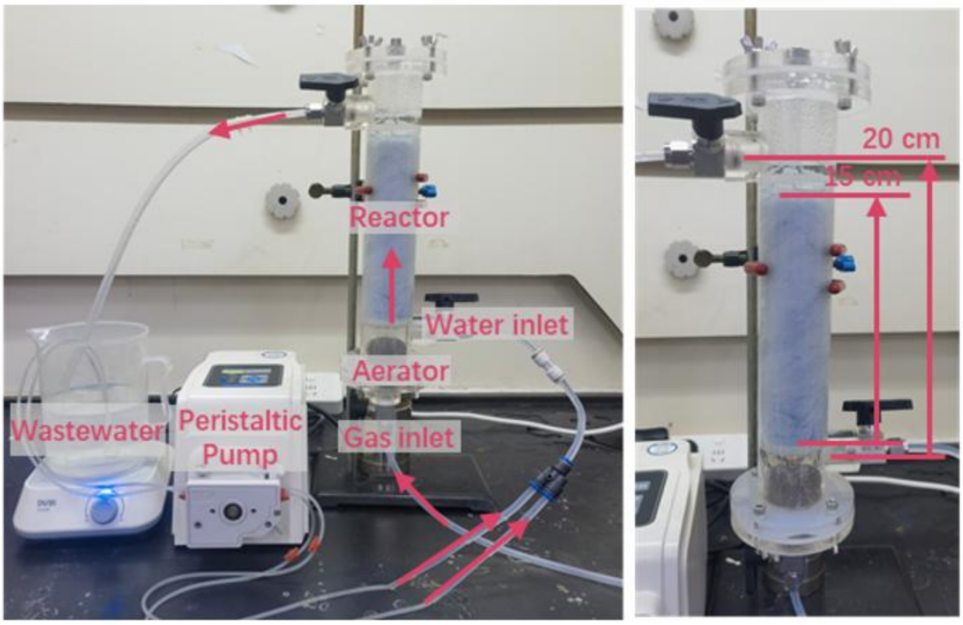

Supplementary Fig. 54 | Picture of the experiment setup for the continuous flow test. Illustration of continuous flow reactor for treatment of wastewater. Catalyst loading: 0.1 g L^{-1}; temperature: 25 °C; ozone flow rate: 100 mL min^{-1}; ozone concentration: 25 mg L^{-1}; initial pH was adjusted to 3 by adding 0.01 M H_2SO_4/NaOH in solution. [simulant wastewater]$_0$: 25 mg L^{-1} 4-CP and 5 g L^{-1} NaCl.

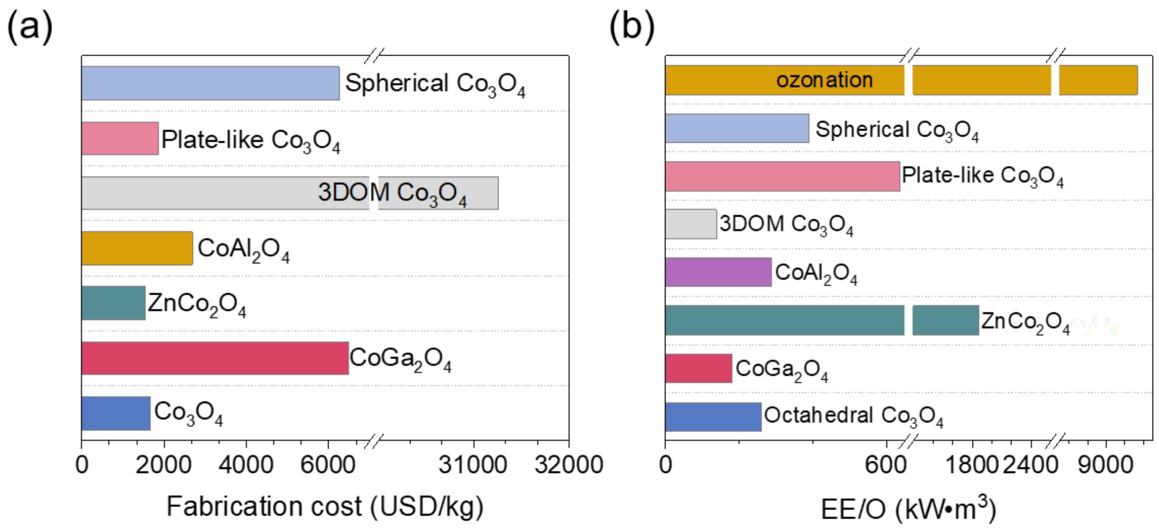

Supplementary Fig. 55 | Economic analysis of different spinel oxides. Comparison of synthesis costs for different Co-based spinels **(a)** and EE/O **(b)** for different reaction systems.

Calculation of synthesis cost of the catalysts

Synthesis cost of the catalysts includes the expense of the consumed reagents and electricity, which can be calculated based on our previously reported work [36].

$$\text{Cost} = R_{Price} \cdot Dosage + P \cdot t \cdot E_{Price} \quad (9)$$

where, R_{Price} is the unit-price of different reagents (USD/g), *Dosage* is the reagent dosage (g), P is the input power of instruments (kW), t is the operating time (h), E_{Price} is the cost of electricity published by State Grid Corporation of China (0.07 USD/kW·h). Detailed information for the cost assessment of the catalysts synthesis is provided in **Supplementary Data 2**.

Calculation of expense of electrical energy in form of the electrical energy per order (EE/O)

The EE/O (kW·h/m^3) is defined as the electrical energy (kW·h) necessary to diminish the concentration of contaminants by one order of magnitude per cubic meter of contaminated water [36]. This metric serves

as an essential reference for scaling up processes, performing economic analyses, and comparing energy efficiency across various treatment technologies. The EE/O was calculated as follows:

$$EE/O = \frac{P \cdot t}{V \cdot \log(\frac{C_f}{C_i})} \tag{10}$$

where, P is the rated power (kW) of the catalytic ozonation system, t is the reaction time (min), V is the volume (m^3) of the simulant wastewater solution in the reactor. C_i and C_f are the initial and final states OA concentration, respectively and provided in **Supplementary Table 13**.

References

1. Bader H, *et al.* Determination of ozone in water by the indigo method. Water Res. **4**, 449-456 (1981).
2. Zhao M, *et al.* Crystal facet regulation and Ru incorporation of Co_3O_4 for acidic oxygen evolution reaction electrocatalysis. ACS Nanosci. Au **6**, 409-415 (2024).
3. Zhou L, *et al.* Facet effect of Co_3O_4 nanocatalysts on the catalytic decomposition of ammonium perchlorate. J. Hazard. Mater. 122358 (2020).
4. Wang H, *et al.* Oriented generation of $^1O_2$ from peroxymonosulfate via Co_3O_4 facet engineering. Appl. Catal. B Environ. 124854 (2025).
5. Wen P, *et al.* Engineering the crystal facets of Co_3O_4 nanostructures for supercapacitor and SERS applications. Electrochim. Acta 145588 (2025).
6. Jian Y, *et al.* Efficient propane low-temperature destruction by Co_3O_4 crystal facets engineering: Unveiling the decisive role of lattice and oxygen defects and surface acid-base pairs. Appl. Catal. B Environ. 119657 (2021).
7. Zhai G, *et al.* Boosting soot combustion efficiency of Co_3O_4 nanocrystals via tailoring crystal facets. Chem. Eng. J. 488-498 (2018).
8. Wang C, *et al.* Catalytic reactivity of Co_3O_4 with different facets in the hydrogen abstraction of phenol by persulfate. Appl. Catal. B Environ. 118819 (2020).
9. Liu Y, *et al.* Clarifications of concepts concerning interplanar spacing in crystals with reference to recent publications. SN Appl. Sci. 1-29 (2020).
10. Hu Q, *et al.* Designing efficient nitrate reduction electrocatalysts by identifying and optimizing active sites of Co-based spinels. J. Am. Chem. Soc. **5**, 2967-2976 (2024).
11. Liu Z, *et al.* Optimal geometrical configuration of cobalt cations in spinel oxides to promote oxygen evolution reaction. Angew. Chem. Int. Ed. **12**, 4766-4772 (2020).
12. Wang X, *et al.* Geometrical-site-dependent catalytic activity of ordered mesoporous Co-based spinel for benzene oxidation: in situ DRIFTS study coupled with Raman and XAFS spectroscopy. ACS Catal. **3**, 1626-1636 (2017).

13. Shin C, *et al.* Magnesium: properties and rich chemistry for new material synthesis and energy applications. Chem. Soc. Rev. **6**, 2145-2192 (2023).
14. Marcos R, *et al.* Novel hierarchical Co_3O_4/ZnO mixtures by dry nanodispersion and their catalytic application in the carbonylation of glycerol. J. Catal. **2**, 288-293 (2010).
15. Ghosh D, *et al.* Theoretical calculation of absolute radii of atoms and ions. part 2. the ionic radii. Int. J. Mol. Sci. **6**, 379-407 (2003).
16. Ghosh D, *et al.* Theoretical calculation of absolute radii of atoms and ions. part 1. the atomic radii. Int. J. Mol. Sci. **2**, 87-113 (2002).
17. Qu J, *et al.* Determination of crystallographic orientation and exposed facets of titanium oxide nanocrystals. Adv. Mater. **37**, 2203320 (2022).
18. Tang P, *et al.* Covalency competition induced active octahedral sites in spinel cobaltites for enhanced pseudocapacitive charge storage. Adv. Energy Mater. **2**, 2102053 (2021).
19. Ravel B, *et al.* Athena, artemis, hephaestus: data analysis for X-ray absorption spectroscopy using IFEFFIT. J. Synchrotron Radiat. **4**, 537-541 (2005).
20. Yang W, *et al.* Enhancement of copper catalyst stability for catalytic ozonation in water treatment using ALD overcoating. ACS Appl. Mater. Interfaces **50**, 43323-43326 (2018).
21. Sadakane M, *et al.* Preparation of nano-structured crystalline tungsten (VI) oxide and enhanced photocatalytic activity for decomposition of organic compounds under visible light irradiation. Chem. Commun. **48**, 6552-6554 (2008).
22. Zhao M, *et al.* Roles of surface-active oxygen species on 3DOM cobalt-based spinel catalysts $M_xCo_{3-x}O_4$ (M = Zn and Ni) for NO_x-assisted soot oxidation. ACS Catal. **8**, 7548-7567 (2019).
23. Xu Z, *et al.* Understanding spatial effects of tetrahedral and octahedral cobalt cations on peroxymonosulfate activation for efficient pollution degradation. Appl. Catal. B Environ. 120072 (2021).
24. Bae J, *et al.* Facet-dependent Mn doping on shaped Co_3O_4 crystals for catalytic oxidation. ACS Catal. **17**, 11066-11074 (2021).
25. Liu J, *et al.* High temperature Mn_2O_3/Mn_3O_4 and Co_3O_4/CoO systems for thermo-chemical energy storage. J. Environ. Manage. 110582 (2020).
26. Zobel C, *et al.* Evidence for a low-spin to intermediate-spin state transition in $LaCoO_3$. Phys. Rev. B **2**, 020402 (2002).
27. Wang Y, *et al.* Occurrence of both hydroxyl radical and surface oxidation pathways in N-doped layered nanocarbons for aqueous catalytic ozonation. Appl. Catal. B Environ. 283-291 (2019).
28. Meyerstein D. Re-examining Fenton and Fenton-like reactions. Nat. Rev. Chem. **9**, 595-597 (2021).
29. DeRosa M, *et al.* Photosensitized singlet oxygen and its applications. Coord. Chem. Rev. 351-371 (2002).
30. Wu T, *et al.* Iron-facilitated dynamic active-site generation on spinel $CoAl_2O_4$ with self-termination of surface reconstruction for water oxidation. Nat. Catal. **9**, 763-772 (2019).
31. Sun Y, *et al.* Covalency competition dominates the water oxidation structure–activity relationship on spinel oxides. Nat. Catal. **7**, 554-563 (2020).
32. Bhattacharjee S, *et al.* An improved d-band model of the catalytic activity of magnetic transition metal surfaces. Sci. Rep. **2**, 35916 (2016).
33. Xia B, *et al.* Optimized conductivity and spin states in N-doped $LaCoO_3$ for oxygen electrocatalysis. ACS Appl. Mater. Interfaces **2**, 2447-2454 (2021).
34. Laing M. The bonding in the ozone molecule: From a different perspective. Struct. Chem. **6**, 397-402 (1995).
35. Sun Y, *et al.* Engineering high-spin state cobalt cations in spinel zinc cobalt oxide for spin channel propagation

and active site enhancement in water oxidation. Angew. Chem. Int. Ed. **26**, 14536-14544 (2021).
36. Liu Y, *et al.* Nanochanneling and local crystallization engineering accelerate multiphase single-atom catalysis for rapid water decontamination. Angew. Chem. Int. Ed. e202504571 (2025).
