# Supplementary material for: Tailored ozone activation on geometrical-site-dependent cobalt with selective coordination
Source: Nat Commun. 2025 Jul 1;16:5921. doi: 10.1038/s41467-025-61181-7 (PMC12214642; doi:10.1038/s41467-025-61181-7)
Supplement: Supplementary file 2 — Description of Additional Supplementary Files [file 41467_2025_61181_MOESM2_ESM.pdf]

Description of Additional Supplementary Files

File Name: Supplementary Data 1
Description: Comparison of the catalytic ozonation activities using different types of heterogeneous catalysts.

File Name: Supplementary Data 2
Description: Cost assessment of catalyst synthesis.
