# Supplementary material for: Tailored ozone activation on geometrical-site-dependent cobalt with selective coordination
Source: Nat Commun. 2025 Jul 1;16:5921. doi: 10.1038/s41467-025-61181-7 (PMC12214642; doi:10.1038/s41467-025-61181-7)
Supplement: Supplementary file 3 — Supplementary Data 1 [file 41467_2025_61181_MOESM3_ESM.pdf]

Supplementary Data 1. Comparison of the catalytic ozonation activities using different types of heterogeneous catalysts.

Catalyst	Pollutants	Conditions		BET SSAs	Main ROS production	Efficiency	Ref.
δ-MnO_2	p-Chlorophenol	[Cat.] = 0.5 g/L<br>[Poll.] = 100 mg/L	$[O_3]$ = 0.3 mg/min<br>[Time] = 30 min	271.1 m^2/g	•OH, $^1O_2$, $O_2^{\bullet-}$	97% removal<br>0.227 min^{-1}	1
$CaMn_4O_8$	p-Nitrophenol	[Cat.] = 0.1 g/L<br>[Poll.] = 50 mg/L	$[O_3]$ = 5 mg/min<br>[Time] = 60 min	8.8 m^2/g	$^1O_2$, $O_2^{\bullet-}$	99% removal<br>0.125 min^{-1}	2
MWCNTs	Oxalic acid	[Cat.] = 0.1 g/L<br>[Poll.] = 96 mg/L	$[O_3]$ = 20 mg/min<br>[Time] = 40 min	115 m^2/g	•OH	80.5% removal<br>0.0845 min^{-1}	3
Fe_3O_4/MWCNTs	Sulfamethazine	[Cat.] = 0.5 g/L<br>[Poll.] = 20 mg/L	$[O_3]$ = 9 mg/min<br>[Time] = 10 min	130 m^2/g	•OH	97% removal<br>0.60 min^{-1}	4
2% Cu/SBA-15	Reactive Orange 4	[Cat.] = 0.2 g/L<br>[Poll.] = 100 mg/L	$[O_3]$ = 25 mg/min<br>[Time] = 60 min	432.3 m^2/g	•OH	86% removal<br>0.031 min^{-1}	5
Mn-CeO_x/γ-Al_2O_3	Bromaminic acid	[Cat.] = 1.0 g/L<br>[Poll.] = 50 mg/L	$[O_3]$ = 5 mg/min<br>[Time] = 120 min	264.8 m^2/g	•OH	98%<br>0.0917 min^{-1}	6
MgO[111]	Nitrobenzene	[Cat.] = 1.0 g/L<br>[Poll.] = 50 mg/L	$[O_3]$ = 2.5 mg/min<br>[Time] = 30	187.8 m^2/g	•OH	90.6% removal<br>0.0741 min^{-1}	7

			min				
$MgFe_2O_4$	Acid Orange II	[Cat.] = 0.1 g/L<br>[Poll.] = 50 mg/L	$[O_3]$ = 0.5 mg/L<br>[Time] = 40 min	84.2 m^2/g	•OH	94% removal<br>0.0668 min^{-1}	8
MnO_2/rGO	p-NP	[Cat.] = 0.1 g/L<br>[Poll.] = 50 mg/L	$[O_3]$ = 5.0 mg/L<br>[Time] = 60 min	35.2 m^2/g	$^1O_2$, $O_2^{•-}$	99% removal<br>0.123 min^{-1}	9
MWCNTs /Fe_3O_4	Bisphenol A	[Cat.] = 0.5 g/L<br>[Poll.] = 50 mg/L	$[O_3]$ = 0.9 mg/min<br>[Time] = 40 min	93.0 m^2/g	•OH	90% removal<br>0.0579 min^{-1}	10
FeMgO/CNT	phenol	[Cat.] = 1 g/L<br>[Poll.] = 400 mg/L	$[O_3]$ = 1.5 mg/min<br>[Time] = 60 min	120 m^2/g	•OH	79% removal<br>0.010 min^{-1}	11
Fe/KCC	SMT	[Cat.] = 0.3 g/L<br>[Poll.] = 20 mg/L	$[O_3]$ = 6.0 mg/min<br>[Time] = 15 min	464.5 m^2/g	•OH	100% removal<br>0.29 min^{-1}	12
rGO	p-Hydroxylbenzoic Acid<br>p-HBA	[Cat.] = 0.1 g/L<br>[Poll.] = 20 mg/L	$[O_3]$ = 2 mg/min<br>[Time] = 60 min	305 m^2/g	$^1O_2$, $O_2^{•-}$	95% removal<br>0.083 min^{-1}	13

Reference:

1. Li P, *et al.* Highly porous α-MnO_2 nanorods with enhanced defect accessibility for efficient catalytic ozonation of refractory pollutants. J. Hazard. Mater. 129235 (2022).
2. Wang Y, *et al.* Hierarchical shape-controlled mixed-valence calcium manganites for catalytic ozonation of aqueous phenolic compounds. Catal. Sci. Technol. **9**, 2918-2929 (2016).
3. Liu Z, *et al.* Influence of different heat treatments on the surface properties and catalytic performance of carbon nanotube in ozonation. Appl. Catal. B-Environ. **1**, 74-80 (2010).
4. Bai Z, *et al.* Catalytic ozonation of sulfamethazine antibiotics using Fe_3O_4/multiwalled carbon nanotubes. Environ. Prog. Sustain. Energy **2**, 678-685 (2018).
5. Ghuge S, *et al.* Ozonation of reactive Orange 4 dye aqueous solution using mesoporous Cu/SBA-15 catalytic material. J. Water Process. Eng. 217-229 (2018).
6. Wu Z, *et al.* Insights into mechanism of catalytic ozonation over practicable mesoporous Mn-CeO_x/γ-Al_2O_3 catalysts. Ind. Eng. Chem. Res. **6**, 1943-1953 (2018).
7. Chen J, *et al.* Efficient degradation of nitrobenzene by an integrated heterogeneous catalytic ozonation and membrane separation system with active MgO (111) catalyst. Water Treat. **8**, 2168-2180 (2015).
8. Lu J, *et al.* Role of Mg in mesoporous $MgFe_2O_4$ for efficient catalytic ozonation of Acid Orange II. J. Chem. Technol. Biotechnol. **4**, 985-993 (2016).
9. Wang Y, *et al.* 2D/2D nano-hybrids of γ-MnO_2 on reduced graphene oxide for catalytic ozonation and coupling peroxymonosulfate activation. J. Hazard. Mater. 56-64 (2016).
10. Huang Y, *et al.* Combined adsorption and catalytic ozonation for removal of endocrine disrupting compounds over MWCNTs/Fe_3O_4 composites. Catal. Today 143-150 (2017).
11. Nguyen T, *et al.* Novel FeMgO/CNT nano composite as efficient catalyst for phenol removal in ozonation process. Mater. Res. Express **9**, 095603 (2018).
12. Bai Z, *et al.* Iron doped fibrous-structured silica nanospheres as efficient catalyst for catalytic ozonation of sulfamethazine. Environ. Sci. Pollut. Res. 10090-10101 (2018).
13. Wang Y, *et al.* Efficient catalytic ozonation over reduced graphene oxide for p-Hydroxylbenzoic Acid (PHBA) destruction: active site and mechanism. ACS Appl. Mater. Interfaces **15**, 9710-9720 (2016).
