# Supplementary material for: Tailored ozone activation on geometrical-site-dependent cobalt with selective coordination
Source: Nat Commun. 2025 Jul 1;16:5921. doi: 10.1038/s41467-025-61181-7 (PMC12214642; doi:10.1038/s41467-025-61181-7)
Supplement: Supplementary file 4 — Supplementary Data 2 [file 41467_2025_61181_MOESM4_ESM.pdf]

Supplementary Data 2. Cost assessment of catalyst synthesis.

Catalyst	Item	Unit-price	Dosage	Cost (USD)	Productivity (g)	Price (USD/kg)
Octahedral Co_3O_4	Citric acid monohydrate	0.002 USD/g	5.674 g	0.0113	1.02	1660
	$CoCl_2{\cdot}6H_2O$	0.126 USD/g	4.283 g	0.54		
	Magnetic stirrers	0.07 USD/kW·h	0.6 kW·h	0.042		
	Oven	0.07 USD/kW·h	7.2 kW·h	0.504		
	Muffle furnace	0.07 USD/kW·h	8.5 kW·h	0.595		
$ZnCo_2O_4$	Citric acid monohydrate	0.002 USD/g	5.67 g	0.0113	1.04	1550
	$ZnCl_2$	0.117 USD/g	0.818 g	0.096		
	$CoCl_2{\cdot}6H_2O$	0.126 USD/g	2.855 g	0.36		
	Magnetic stirrers	0.07 USD/kW·h	0.6 kW·h	0.042		
	Oven	0.07 USD/kW·h	7.2 kW·h	0.504		
	Muffle furnace	0.07 USD/kW·h	8.5 kW·h	0.595		
$CoGa_2O_4$	Citric acid monohydrate	0.002 USD/g	5.67 g	0.0113	1.12	6470
	$GaCl_2$	2.8 USD/g	2.113 g	5.92		
	$CoCl_2{\cdot}6H_2O$	0.126 USD/g	1.43 g	0.18		
	Magnetic stirrers	0.07 USD/kW·h	0.6 kW·h	0.042		
	Oven	0.07 USD/kW·h	7.2 kW·h	0.504		
	Muffle furnace	0.07 USD/kW·h	8.5 kW·h	0.595		
3DOM Co_3O_4	Citric acid monohydrate	0.002 USD/g	6.3 g	0.0126	0.20	31258
	$Co(NO_3)_2{\cdot}6H_2O$	0.062 USD/g	8.73 g	0.541		
	Methanol	0.023 USD/mL	10 mL	0.23		
	Polymethyl methacrylate	2.67 USD/g	1.5 g	13.005		

	Magnetic stirrers	0.07 USD/kW·h	0.4 kW·h	0.028		
	Oven	0.07 USD/kW·h	12 kW·h	0.84		
	Muffle furnace	0.07 USD/kW·h	8.5 kW·h	0.595		
Plate-like Co_3O_4	Citric acid monohydrate	0.002 USD/g	6.3 g	0.0126	1.21	1860
	$Co(NO_3)_2{\cdot}6H_2O$	0.062 USD/g	8.73 g	0.541		
	Methanol	0.023 USD/mL	10 mL	0.23		
	Magnetic stirrers	0.07 USD/kW·h	0.4 kW·h	0.028		
	Oven	0.07 USD/kW·h	12 kW·h	0.84		
	Muffle furnace	0.07 USD/kW·h	8.5 kW·h	0.595		
Spherical Co_3O_4	NaOH	0.14 USD/g	1.2 g	0.168	1.32	6250
	$Co(NO_3)_2{\cdot}6H_2O$	0.062 USD/g	116.4 g	7.2168		
	Magnetic stirrers	0.07 USD/kW·h	0.2 kW·h	0.014		
	Oven	0.07 USD/kW·h	3.6 kW·h	0.252		
	Muffle furnace	0.07 USD/kW·h	8.5kW·h	0.595		
$CoAl_2O_4$	Citric acid monohydrate	0.002 USD/g	5.67 g	0.0113	1.02	2682
	$AlCl_3{\cdot}6H_2O$	0.44 USD/g	2.90 g	1.276		
	$CoCl_2{\cdot}6H_2O$	0.126 USD/g	0.009 g	0.18		
	Magnetic stirrers	0.07 USD/kW·h	0.6 kW·h	0.042		
	Oven	0.07 USD/kW·h	7.2 kW·h	0.504		
	Muffle furnace	0.07 USD/kW·h	9 kW·h	0.72		
